# Differential Regulation of Maize and Sorghum Orthologs in Response to the Fungal Pathogen *Exserohilum turcicum*

**DOI:** 10.3389/fpls.2021.675208

**Published:** 2021-05-25

**Authors:** Pragya Adhikari, Santiago X. Mideros, Tiffany M. Jamann

**Affiliations:** Department of Crop Sciences, University of Illinois at Urbana-Champaign, Urbana, IL, United States

**Keywords:** quantitative disease resistance, transcriptome, host resistance, maize (*Zea mays* L.), sorghum, *Exserohilum turcicum*

## Abstract

Pathogens that infect more than one host offer an opportunity to study how resistance mechanisms have evolved across different species. *Exserohilum turcicum* infects both maize and sorghum and the isolates are host-specific, offering a unique system to examine both compatible and incompatible interactions. We conducted transcriptional analysis of maize and sorghum in response to maize-specific and sorghum-specific *E. turcicum* isolates and identified functionally related co-expressed modules. Maize had a more robust transcriptional response than sorghum. *E. turcicum* responsive genes were enriched in core orthologs in both crops, but only up to 16% of core orthologs showed conserved expression patterns. Most changes in gene expression for the core orthologs, including hub genes, were lineage specific, suggesting a role for regulatory divergent evolution. We identified several defense-related shared differentially expressed (DE) orthologs with conserved expression patterns between the two crops, suggesting a role for parallel evolution of those genes in both crops. Many of the differentially expressed genes (DEGs) during the incompatible interaction were related to quantitative disease resistance (QDR). This work offers insights into how different hosts with relatively recent divergence interact with a common pathogen. Our results are important for developing resistance to this critical pathogen and understanding the evolution of host-pathogen interactions.

## Introduction

Host expansions and shifts are a major threat to global food security but offer a unique opportunity to understand host resistance (Zhong et al., 2016; Corredor-Moreno and Saunders, 2020). Understanding conserved defense mechanisms in related crop species has important implications for the management of pathogens infecting multiple crops using host resistance. The identification of conserved resistance mechanisms across crop species and translation from one species to another can help in the development of resistant cultivars in new crop species and preparing for future emergencies (Dangl et al., 2013). In the case of host-specific pathotypes that resulted from pathogen host jumps, insights into the genetic factors involved in conferring host resistance enable the manipulation of these factors in susceptible hosts. Additionally, understanding how genes and expression patterns are conserved across species in response to common biotic stressors, and how the regulation of such genes affects phenotype in the host is important to understand plant–pathogen co-evolution.

*Exserohilum turcicum* (syn. *Setosphaeria turcica*) co-evolved with maize in Mexico and subsequently jumped from maize to sorghum (Borchardt et al., 1998). Maize and sorghum have a close evolutionary relationship and diverged from a common ancestor ∼12 million years ago (Swigonova et al., 2004). Both maize and sorghum belong to the monophyletic grass tribe Andropogoneae, which is a major tribe in the subfamily Panicoideae and includes one third of all grass species (Spangler et al., 1999). Maize is an ancient tetraploid that underwent a genome duplication resulting in two distinct maize subgenomes, maize1 and maize2 (Gaut and Doebley, 1997; Schnable et al., 2011).

Although the same species of *Exserohilum* infects both maize and sorghum, the host-pathogen interaction is specific, such that isolates pathogenic on maize are generally not pathogenic on sorghum and vice-versa (Hamid and Aragaki, 1975; Nieuwoudt et al., 2018). While genetic differentiation has been observed between host-specific isolates, there is evidence of gene flow between maize-specific and sorghum-specific *E. turcicum* isolates, suggesting host specialization of the pathogen (Nieuwoudt et al., 2018). Currently, two host-specific formae speciales of *E. turcicum* are recognized; *E. turcicum* f. sp. *zeae* is only pathogenic on maize and causes northern corn leaf blight (NCLB), while *E. turcicum* f. sp. *sorghi* is only pathogenic on sorghum and causes sorghum leaf blight (SLB). A single gene has been reported for host specificity of the pathogen in sorghum (sorA ^+^) and maize (zeaA ^+^) (Hamid and Aragaki, 1975), but little is understood about the host responses to incompatible isolates.

The diseases of maize and sorghum caused by *E. turcicum* are severe. In maize, the estimated yield loss in the United States and Canada caused by NCLB was 27.9 million metric tons between 2012 and 2015, the most extensive loss among all foliar diseases (Mueller et al., 2016). Similarly, SLB can decrease grain yields up to 50%, reduce forage quantity and quality, and predispose plants to other diseases such as Anthracnose stalk rot (Frederiksen, 1986). Furthermore, the high evolutionary potential of *E. turcicum*, characterized by the incidence of sexual reproduction of the pathogen in the field and high genetic variation in terms of virulence, genetic structure, races, cultural characteristics and aggressiveness (McDonald and Linde, 2002; Galiano-Carneiro and Miedaner, 2017), pose a future threat.

In maize, both qualitative (major genes such as *Ht1*, *Ht2*, *Ht3*, *HtN*, *HtM*, and *ht4*) and quantitative resistance have been reported for NCLB (Wisser et al., 2006; Galiano-Carneiro and Miedaner, 2017). The major genes either significantly slow the onset of lesions or restrict lesions, but do not confer complete resistance. An incompatible response only occurs when an incompatible formae speciales is used to inoculate a non-host for that strain (i.e., *E. turcicum* f. sp. *sorghi* is used to inoculate maize). Few studies have been conducted in sorghum on host resistance to *E. turcicum* (Sharma et al., 2012; Beshir et al., 2016). There is some evidence for shared resistance between maize and sorghum against *E. turcicum* (Martin et al., 2011). A genome wide association study (GWAS) on the sorghum association panel identified candidate sorghum resistance genes with conserved function in maize (Zhang et al., 2020). However, no previous studies have explored transcriptional responses to *E. turcicum* in both maize and sorghum.

We adopted an RNA-sequencing approach to study differential gene expression and gene co-expression in maize and sorghum in response to both maize-specific and sorghum-specific *E. turcicum.* RNA-sequencing is a common method used to study gene expression patterns and has been implemented in several host-pathogen interaction studies (Zhu et al., 2013; Tan et al., 2015; Xiong et al., 2018; Gómez-Cano et al., 2019; Li et al., 2019; Lu et al., 2019; Sari et al., 2019). Importantly, this approach enabled us to compare and contrast the compatible and incompatible interactions. The relevance of large-scale transcriptomics to understand disease has been emphasized by the omnigenic model (Boyle et al., 2017). According to this model, every gene that is differentially expressed (DE) in relevant cells is likely to contribute to the outcome and are called peripheral genes. However, only few central genes are biologically essential, directly affecting the mechanisms that lead to the trait and are called core or hub genes (Boyle et al., 2017). Understanding these hub genes will offer insights into how the plant controls the defense response, whereas the peripheral genes will elucidate variation observed among different groups.

Our central hypothesis was that maize and sorghum have conserved defense mechanisms that restrict *E. turcicum* growth. The study was designed to test the extent of conserved and lineage-specific gene expression in response to *E. turcicum* in maize and sorghum and identify key hub genes regulating defense pathways in these crops. To examine the relationship between sorghum and maize responses to *E. turcicum*, we inoculated maize and sorghum with host-specific isolates and examined the transcriptional response at two timepoints. The objectives of this study were: (i) to compare and contrast the maize and sorghum transcriptional responses to *E. turcicum* at 24 and 72 hai (hours after inoculation), (ii) to identify candidate genes including core/hub genes involved in the reaction to *E. turcicum* in both maize and sorghum, and (iii) to examine the extent of conservation in gene expression in compatible and incompatible interactions between maize and sorghum. These findings offer insight into host resistance in maize and sorghum and the evolution of plant–pathogen interactions.

## Materials and Methods

### Greenhouse Experimental Design, Fungal Inoculation, and Sample Collection

The sorghum line BTx623 and maize line B73 were selected because reference genomes are available for these lines (Paterson et al., 2009; Schnable et al., 2009). BTx623 is a host for *E. turcicum* f. sp. *sorghi* and forms a compatible interaction upon infection by *E. turcicum* f. sp. *sorghi*; whereas B73 is a host for *E. turcicum* f. sp. *zeae* and forms a compatible interaction upon infection by *E. turcicum* f. sp. *zeae* (Table 1). Seeds for BTx623 and B73 were obtained from germplasm resource information network (GRIN). Two strains of *E. turcicum* were selected for inoculations: Et28A (maize-specific; received from B.G. Turgeon, Cornell University) (Ohm et al., 2012) and 15St008 (sorghum-specific; isolated from symptomatic sorghum in Illinois) (Zhang et al., 2020). There were three types of interactions: compatible (Et28A on maize and 15St008 on sorghum), incompatible (Et28A on sorghum and 15St008 on maize), and control (mock inoculation on maize and mock inoculation on sorghum) (Table 1).

**TABLE 1:** Interaction groups between hosts and pathogens used in this study.

Host	*Exserohilum turcicum* f. sp. *zeae* (Et28A)	*Exserohilum turcicum* f. sp. *sorghii* (15St008)
**Pathogen**		
*Zea mays* (B73)	Interaction: compatible	Interaction: incompatible
	Pathogen: adapted	Pathogen: non-adapted
	Plant: host	Plant: non-host
*Sorghum bicolor (BTx623)*	Interaction: incompatible	Interaction: compatible
	Pathogen: non-adapted	Pathogen: adapted
	Plant: non-host	Plant: host

The inoculation experiments were conducted at the Plant Care Facility at the University of Illinois at Urbana-Champaign in two sets: one for RNA-sequencing and one for quantitative reverse transcriptase polymerase chain reaction (qRT-PCR). For each set, the experiment was completely randomized with three replicates and three treatments (Et28A, 15St008, and mock), for a total of nine maize plants and nine sorghum plants. Pathogen isolates were cultured on lactose-casein hydrolysate agar (LCA) media (Tuite, 1969) for 2 weeks under 12 h/12 h light/dark environment at room temperature. Spores were collected and the concentration was adjusted to 4 × 10^3^ spores/ml in a 0.02% Tween 20 solution (Chung et al., 2010). Plants were inoculated at the V3 stage (Abendroth et al., 2011) by pipetting 0.5 ml of the spore solution into the whorl. Mock inoculations were conducted with 0.02% Tween 20. After inoculation, plants were maintained in high humidity conditions overnight to facilitate disease development.

Infected leaf tissue was collected at two time points including 24 h after inoculation (hai) and 72 hai. We chose these time points because preliminary observations indicated that both strains penetrated sorghum at 24 hai and the first microscopic symptoms developed within 72 hai in the compatible interaction. The latest time point was 72 hai because we were interested in understanding the early defense response in the host during compatible and incompatible interactions. In total, there were 64 total samples including 34 samples for RNA sequencing and 34 for qRT-PCR. All samples were collected at 4:20 pm, and samples were immediately placed in liquid nitrogen.

### RNA Extraction and Sequencing

RNA was extracted using TriZol (Thermo Fisher Scientific, Waltham, MA, United States) and an RNAeasy miniElute cleanup kit (QIAGEN, Germantown, MD, United States), as described by Fall et al. (2019). The RNA quality and integrity were checked by running the RNA on a 1.0% agarose gel. A total of 36 RNA samples (18 maize samples and 18 sorghum samples) were submitted for library preparation and sequencing at Roy J. Carver Biotechnology Center at the University of Illinois at Urbana-Champaign. The RNAseq libraries were prepared using a TruSeq Stranded mRNAseq Sample Prep kit (Illumina, San Diego, CA, United States) and quantified using qRT-PCR. The samples were individually barcoded, pooled randomly to avoid lane effects and sequenced over three lanes for 101 cycles on a NovaSeq 6000 (Illumina) using 100 bp single-end sequencing. The data are available at the NCBI GEO repository with accession number GSE156026.

### Differential Gene Expression Analysis

Adaptors were trimmed from the 3′-end of the reads by the sequencing facility. The initial quality control for the raw reads, including analysis of sequence quality, GC content, the presence of adaptors, overrepresented k-mers and duplicated reads, was performed using FastQC (Andrews, 2010). The reference genomes for maize (GCF_000005005.2_B73_RefGen_v4) and sorghum (Sbicolor_454v3) were downloaded from NCBI and Phytozome, respectively, and indexed. The maize and sorghum reads were aligned to their respective genomes using the splice-aware aligner STAR v2.7 (Dobin et al., 2013). The read counts per gene were then quantified from the alignments using “featureCounts” in the subread package (version1.6.3) (Liao et al., 2014). Multi-mapping reads and reads with ambiguous assignments were removed.

The gene counts were imported into R version 3.6.0 (R Core Team, 2015) for further processing and statistical analysis. The Bioconductor package “edgeR” was used for quality control and normalization (Robinson and Oshlack, 2010; Robinson et al., 2010), while the package “limma” was used to calculate differentially expressed genes (DEGs) (Ritchie et al., 2015). The genes with less than one count per million (CPM) in more than three samples were filtered out. The trimmed mean of M-values (TMM) normalization factors were calculated to correct for different library sizes and composition bias and to obtain normalized log_2_CPM values with a prior count of three. The log normalized count data were used for limma-trend analysis to calculate DEGs using the “limma” package (Ritchie et al., 2015). The mean-variance trend was adjusted using empirical Bayes “shrinkage” of variances, and the test statistics were calculated using the eBayes function of the “limma” package (Smyth, 2004; Ritchie et al., 2015; Phipson et al., 2016). A global false discovery rate (FDR) correction was performed to account for the differences in the number of significant DEGs in each pairwise comparison.

We were interested in four combinations of interactions and time points for each host: compatible at 24 hai, compatible at 72 hai, incompatible at 24 hai, and incompatible at 72 hai. The DEGs for each of these interactions were calculated by contrasting the expression data of the mock treatment at the corresponding time points. The genes were considered DE if the gene expression fold change was greater than two between the group and mock and if the FDR value was less than 0.05. The functional significance of the DEGs in the four interaction and time point combinations of maize and sorghum were determined using singular enrichment analysis (SEA) with agriGO v2.0 (Tian et al., 2017). The total expressed genes were used as a background for SEA analysis.

### Comparing Expression Patterns Between Maize and Sorghum

To compare expression patterns in response to *E. turcicum* between sorghum and maize, we obtained a list of syntenic orthologous gene pairs for maize and sorghum from Schnable (2019) generated using the methodology described by Zhang et al. (2017). The genes with syntenic orthologs in both maize and sorghum were considered core genes. We then compared the expression patterns of orthologous gene pairs within each interaction-time combination (i.e., compatible interaction at 24 hai, compatible interaction at 72 hai, incompatible interaction at 24 hai, and incompatible interaction at 72 hai). The syntenic orthologs that were DE in both maize and sorghum were classified as shared differentially expressed orthologs (shared DEOs). The expected number of DEGs in both species was calculated as the percentage of differentially expressed (DE) core gene pairs in maize times the percentage of DE core gene pairs in sorghum times the total number of gene pairs analyzed (Zhang et al., 2017). A chi-squared test was performed to test the null hypothesis of no conservation of gene expression between maize and sorghum. The alternative hypothesis was that gene expression is conserved between maize and sorghum and that the observed conservation of gene expression is more than would be expected by chance.

### Gene Co-expression Network Analysis

Co-expression analysis was performed for the genes that passed initial filtering in each maize and sorghum using the “Weighted Correlation Network Analysis (WGCNA)” package in R (Langfelder and Horvath, 2008). The function “blockwiseModules” from the WGCNA package was used to identify modules with a tree cut height of 0.2, signed hybrid network, biweight midcorrelation, minimum module size of 20, maximum block size of 2,500, and all other options set to default. This function initially constructed the matrix of pairwise correlations for all pairs of genes across all samples in each dataset (i.e., maize and sorghum) using biweight midcorrelations. Then, the correlation matrix was raised to the power of soft thresholding parameter β (β = 5 for maize and β = 9 for sorghum) to construct the adjacency matrix and generate a scale-free network. The topological overlap measure (TOM) was calculated from the adjacency matrix to measure the connection strength between all gene pairs. Then, the TOM dissimilarity matrix (1-TOM) was calculated and average linkage hierarchical clustering was performed to generate a clustering tree, where modules represented the branches of the tree. Using dynamic hybrid tree cutting, the branches were trimmed to a cut height of 0.2 to identify co-expressed modules. The modules were visualized using heatmaps to check for coherency and uniqueness. The heatmaps were generated using the “heatmap.2” function of the “gplots” package (Warnes et al., 2016).

The average expression pattern of all the genes in each module was summarized using the module eigengene (ME), the first principal component. The ME values were used for limma-trend analysis. The modules significantly associated (FDR < 0.05) with each interaction and time combinations, i.e., compatible interaction at 24 hai, compatible interaction at 72 hai, incompatible interaction at 24 hai, and incompatible interaction at 72 hai, were detected using the “limma” package (Ritchie et al., 2015). We annotated significant modules and then performed a gene ontology (GO) analysis using SEA with agriGO v2.0 (Tian et al., 2017) to test for significant modules with enrichment in genes and functions related to defense.

### Hub Gene Identification

We selected significant modules (FDR < 0.05) enriched in defense-related GO terms to identify hub genes. Hub genes were identified using two methods: (i) module membership (MM) and gene significance (GS), and (ii) number of connections of a node in the gene network. The MM, also known as eigengene based connectivity (kME) was calculated by measuring the correlation between gene expression and ME. An MM value close to 1 indicates high connectivity between the gene and module, whereas an MM value close to 0 indicates that the gene is not a part of the module. The GS was estimated based on the −log10 (*p*-value) obtained from contrasts between the treatment group and mock using the “edgeR” package (Robinson et al., 2010). The genes with high MM and GS values in each module were identified as hub genes.

To calculate the number of connections of a node, a gene network was constructed by extracting the unweighted network of the modules of interest using the threshold of TOM > 0.2 and importing into Cytoscape v. 3.7.2 (Shannon et al., 2003). The genes with a larger number of connections of a node in the gene network were identified as hub genes.

### Validation of DEGs by Using qRT-PCR

Three highly expressed genes, specifically *PR-5* (*GRMZM2G402631*) from maize, and *Sobic.001G020200* and *Sobic.005G101500* from sorghum, were selected to validate the RNA-seq expression data using qRT-PCR. The gene specific primers and TaqMan^®^ probes were designed for each gene using the Integrated DNA Technology (IDT) PrimerQuest Tool^1^ according to the IDT guidelines and synthesized by Thermo Fisher Scientific (Supplementary Table 1). The *PP2A* gene (Sudhakar Reddy et al., 2016) and the validated assay *Zm04040368_g1* for ubiquitin (Jamann et al., 2016) were used as the internal controls for sorghum and maize, respectively.

A total of 36 independent RNA samples (18 maize samples and 18 sorghum samples) different from that of the RNA-sequencing samples were used for qRT-PCR validation. The cDNA was synthesized from mRNA using the ProtoScript M-MuLV First Strand cDNA Synthesis Kit (New England Biolabs) following the manufacturer protocol. The qRT-PCR reactions were performed using a QuantStudio^TM^ 3 System (Applied Biosystems) with a total reaction volume of 20μl using PerfeCTa^®^ qPCR ToughMix, UNG, Low ROX^TM^ kit (QuantaBio, Beverly, MA, United States) according to the manufacturer protocol. Each reaction contained 2 μl of cDNA at a concentration of 400 ng/μl. The final concentration of primers and probe in the reaction were 450 and 125 nM, respectively. Three technical replicates were included for each sample. The amplification program consisted of one 30s cycle of thermo-start polymerase activation at 95°C and 40 cycles of denaturation at 95°C for 5s and annealing/extension at 60°C for 30s. The efficiency of all Taqman assays including internal controls were tested using a qPCR standard curve using a 10-fold serial dilution of cDNA with concentrations ranging from 2 to 20,000 pg/μl. The data were analyzed using the comparative *C*_*T*_ method (Schmittgen and Livak, 2008).

## Results

### Transcriptional Response of Maize and Sorghum to *E. turcicum*

To examine the maize and sorghum response to compatible and incompatible *E. turcicum* strains, we sequenced the RNA of 36 samples from mock and *E. turcicum* inoculated maize and sorghum at 24 and 72 hai. At these time points, we observed flecking on both maize and sorghum as infection is still early in process and lesions do not develop until 14 days post inoculation (Supplementary Figure 1). Sorghum developed red flecks during both compatible and incompatible interaction. We obtained over 1.5 billion reads with Qscores > 35. For each library, the number of single reads ranged from 34 to 65M, with an average of 43.6M reads per library. More than 90% of the total reads obtained from sorghum mapped to the sorghum reference genome uniquely, and at least 80% of the total reads mapped to exons within genes (Supplementary Figure 2). Likewise, more than 80% of the maize reads uniquely mapped to the maize genome, and at least 80% of the reads were mapped to exons within genes (Supplementary Figure 2). After filtering, a total of 22,684 expressed maize genes (50.1% of the maize genome) and 17,731 expressed sorghum genes (61.1% of the sorghum genome) were retained for further analysis. Of the 22,684 expressed maize genes, 10,575 genes (46.6%) belonged to the maize1 subgenome, 6,455 genes (28.5%) belonged to the maize2 subgenome and 5,654 (24.9%) genes were not assigned to a subgenome. The larger number of expressed genes belonging to the maize1 subgenome observed in our study is proportional to the dominance and larger genome size of the maize1 subgenome (Schnable et al., 2011). Overall, we obtained sufficient reads with high quality scores for both maize and sorghum to conduct differential gene expression analysis.

To validate the RNA-seq dataset for the DEGs, we selected three highly expressed genes and performed qRT-PCR on 36 RNA samples that were different from those used for RNA-seq. The relative expression patterns of these genes were consistent in both the RNA-seq and qRT-PCR experiments. However, there were differences in the magnitude of fold changes between the two experiments (Supplementary Figure 3).

### More DEGs Were Observed During the Compatible Interaction at 24 hai and the Incompatible Interaction at 72 hai Compared to Other Interactions and Time Points

We analyzed differential gene expression for the maize and sorghum responses to *E. turcicum* in comparisons to mock samples for four interactions and time point combinations: (i) compatible at 24 hai, (ii) compatible at 72 hai, (iii) incompatible at 24 hai, and (iv) incompatible at 72 hai (Table 1). At 24 hai in both maize and sorghum, more genes were DE during the compatible (129 in maize and 818 in sorghum) than the incompatible interaction (65 in maize and 326 in sorghum). Conversely, at the later time point (72 hai) in both maize and sorghum, more genes were DE during the incompatible interaction (460 in maize and 783 in sorghum) than during the compatible interaction (171 in maize and 171 in sorghum) (Supplementary Table 2). In summary, there were more DEGs during the incompatible interaction at the later time point in both crops.

### Conserved Function of *Exserohilum turcicum* Responsive Genes in Maize and Sorghum

An enrichment analysis was conducted to assess the functional classification of the DEGs in both maize and sorghum. In maize, 19,842 of 22,684 expressed genes (87%) were functionally annotated with GO terms. In sorghum, only 9,598 of 17,731 expressed genes (54%) were functionally annotated with GO terms. More GO terms were significantly (FDR < 0.05) enriched in maize, as compared to sorghum. The interaction-timepoint combination with the largest number of significantly enriched GO terms was the incompatible interaction at 72 hai in both maize (312 terms) and sorghum (53 terms) (Supplementary Table 3).

**FIGURE 1:**
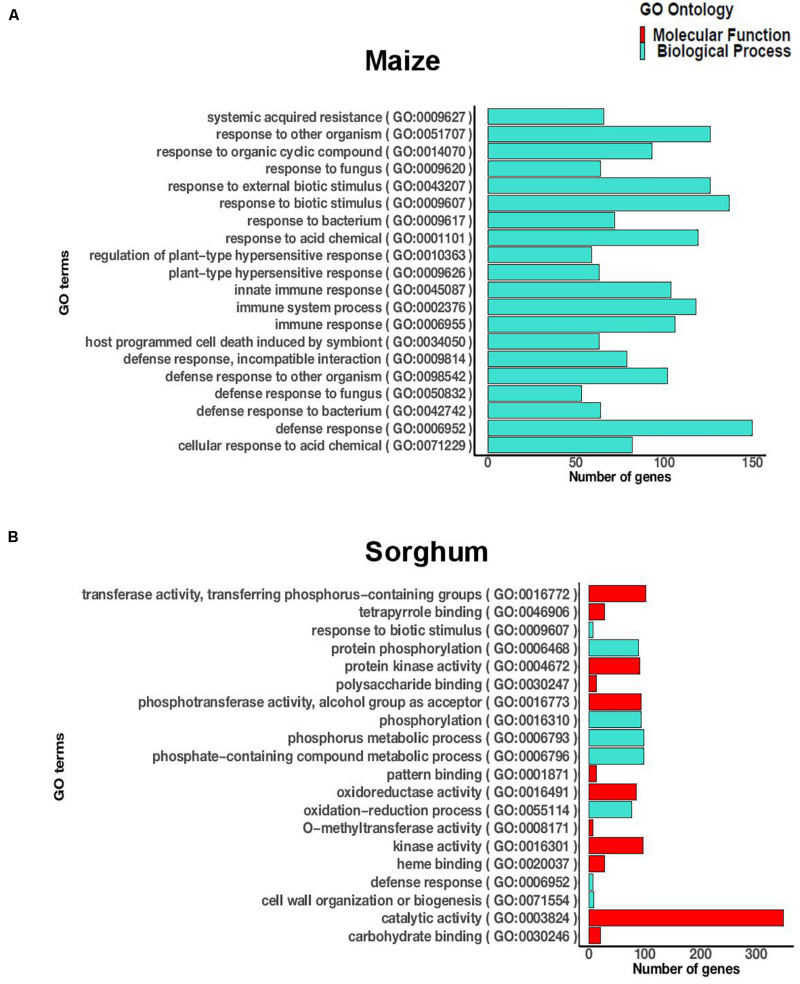
The 20 most significantly enriched GO terms (FDR < 0.05) during the incompatible interaction at 72 h after inoculation in **(A)** maize and **(B)** sorghum.

Since the incompatible interaction, characterized by complete resistance, was of particular interest and more DEGs were observed at the later time point (72 hai), we focused on the incompatible interaction at 72 hai. The significantly enriched GO terms for the incompatible interaction at 72 hai in maize included 237 terms in biological process, 54 terms in molecular function, and 21 terms in cellular process (Supplementary Table 4). The highly significant terms included defense response to a fungus, defense response to a bacterium, immune response, hypersensitive response, and systemic acquired resistance (Figure 1A). In sorghum, the significantly enriched GO terms (FDR < 0.05) for the incompatible interaction at 72 hai included 35 terms in biological process and 18 terms in molecular function (Supplementary Table 5). The significant terms included defense response, response to biotic stimulus, protein kinase activity, catalytic activity, phosphorylation, and oxidoreductase activity (Figure 1B). In both crops, biotic stress related GO terms were significantly enriched during the incompatible interaction at 72 hai.

There were 21 GO terms significantly enriched during the incompatible interaction at 72 hai that were common to maize and sorghum, including eight GO terms in biological process and 13 GO terms in molecular function. The biological process terms included protein phosphorylation, defense response, response to biotic stimulus, metabolic process, multi-organism process, and cell wall organization and biogenesis (Supplementary Table 6). The molecular function terms included protein kinase activity, pattern binding, iron binding, oxidoreductase activity, transferase activity, polysaccharide binding and nucleotide binding (Supplementary Table 6). We observed similar functions of DEGs, specifically related to defense, in both crops in response to *E. turcicum*.

### *Exserohilum turcicum* Responsive Genes Were Enriched in Core Orthologs, Yet There Was Divergent Regulation of Defense in the Two Crops

**FIGURE 2:**
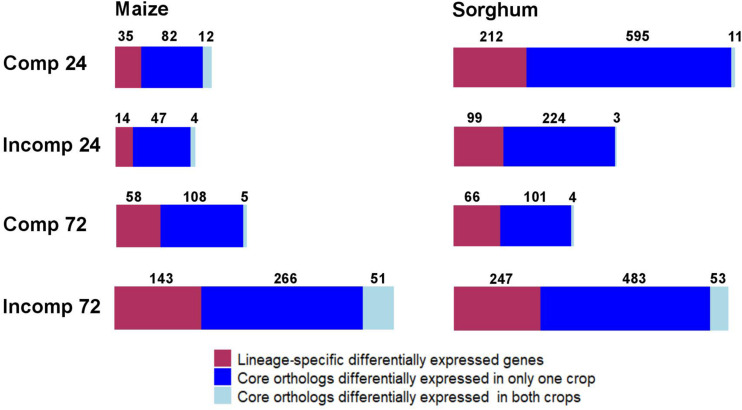
Summary of the number of lineage-specific differentially expressed (DE) genes, core orthologs that were DE in one crop, and core orthologs that were DE in both crops (a.k.a. shared differentially expressed orthologs) in maize and sorghum, in response to *Exserohilum turcicum*. “Comp 24” indicates compatible interaction at 24 h after inoculation (hai), “Incomp 24” indicates incompatible interaction at 24 hai, “Comp 72” indicates compatible interaction at 72 hai and “Incomp 72” indicates incompatible interaction at 72 hai.

To evaluate the degree to which transcriptional responses were conserved between maize and sorghum, we compared the expression of orthologous gene pairs in each crop. Of the 22,684 genes expressed in maize, 14,750 genes (65.0%) belonged to the core group (i.e., have orthologs in sorghum) and 7,934 genes (35.0%) were lineage specific (i.e., no orthologs in sorghum). Similarly, of the 17,731 total expressed genes in sorghum, 13,684 genes (77.2%) belonged to the core group (i.e., have orthologs in maize) and 4,047 genes (22.8%) were lineage specific (i.e., no orthologs in maize). More genes belonging to the core group were DE compared to lineage-specific genes in both sorghum and maize at all interaction and time point combinations (Figure 2). At least 60% of DEGs were core genes in each crop, and the pattern did not vary for the compatible and incompatible interactions (Figure 2).

Only a fraction of core-gene ortholog pairs were DE in both crops. Of the total core DEGs in maize and sorghum, only 1.30–16.0% were DE in both maize and sorghum (Figure 2). A total of 60 gene orthologs were DE in both maize and sorghum in response to *E. turcicum*, which we refer to as shared DE orthologs (shared DEOs) (Supplementary Table 7). The largest percentage of shared DEOs were expressed during the incompatible interaction at 72 hai (Figure 2). Under the null hypothesis of no conservation of gene expression between crops during the incompatible interaction at 72 hai, the expected number of shared DEOs was 12 in maize and 11 in sorghum. The observed number of shared DEOs during the incompatible interaction at 72 hai was around five times higher than the expected number (51 for maize and 53 for sorghum). Thus, we concluded that the enrichment of shared DEOs in both maize and sorghum is not due to random chance based on a chi-squared test (*p* < 0.0001).

### Orthologous Gene Pairs That Were DE in Both Maize and Sorghum (Shared DEOs) Are Enriched in Quantitative Disease Resistance Genes

The largest portion of shared DEOs (∼88%) were for the incompatible interaction at 72 hai (Supplementary Table 7). During the incompatible interaction at 72 hai, DEOs were related to pathogen reception, signal transduction, and the defense response (Supplementary Table 7). Several of the DEOs are either a part of pathogen associated molecular pattern (PAMP)-triggered immunity (PTI), or were activated in PTI-induced leaves in previous studies (Szatmari et al., 2014; Bozso et al., 2016). Additionally, an orthologous gene pair encoding a potassium transporter (*kup1/Sobic.003G413700*; involved in potassium mobilization), a serine carboxypeptidase (*LOC100281606/Sobic.003G081100*; function as acyltransferase), a lactate/malate dehydrogenase (*LOC103632470/Sobic.001G471100*; involved in carboxylic metabolic pathway and oxidation-reduction process), and several orthologous gene pairs encoding cytochrome P450s (involved in biosynthetic and detoxification pathway) were upregulated in both crops (Supplementary Table 7). In general, more than 50% of the shared DEOs in maize and sorghum during the incompatible interaction at 72 hai were related to quantitative disease resistance (QDR), suggesting that shared DEOs are enriched in quantitative resistance genes.

### Responses Are Conserved but Quantitatively Different Between the Compatible and Incompatible Interactions

**FIGURE 3:**
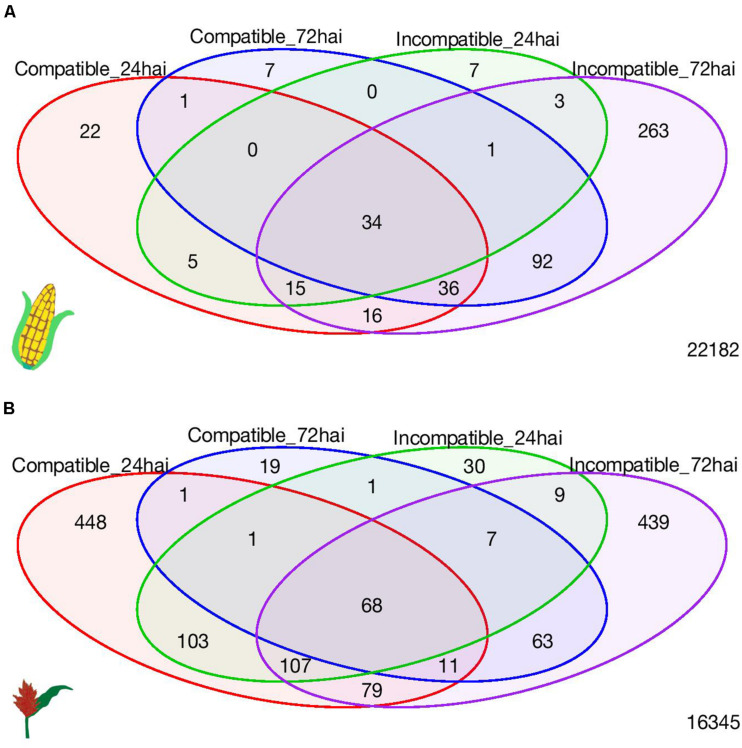
Venn diagram of number of differentially expressed genes among four interaction-time combinations in **(A)** maize and **(B)** sorghum.

A total of 34 (upregulated) maize and 68 sorghum DEGs (67 upregulated and 1 downregulated) were common to all four interaction-time combinations (Figure 3). In maize, these common genes included several defense-related genes including those encoding pathogenesis-related (PR) proteins, protein kinases, and transcription factors (Supplementary Table 8). In sorghum, the common genes among the four interaction-time combinations also included several defense-related genes including those encoding PR proteins, a chitinase, and disease-resistance-family protein with a leucine-rich repeat domain (Supplementary Table 9). In general, pathogenesis-responsive genes and genes related to cell defense, detoxification and cell metabolism were expressed during both the compatible and incompatible interactions in both hosts.

In both maize and sorghum, we observed quantitative differences among the common DEGs related to disease resistance during the compatible and incompatible interaction. There were larger fold change differences at the later time point in both crops (Supplementary Tables 8–9). For instance, the genes encoding a hevein like preprotein (antimicrobial peptides family) (*pco080661a*) and a glucan endo-1,3-beta glucosidase (PR-2 family) (*LOC103633263*, *geb1*) in maize (Supplementary Table 8) and genes encoding a thaumatin-like PR protein-4 (*Sobic.008G182700*) and a PR potein-5 in sorghum (*Sobic.008G183300*) (Supplementary Table 9) were among the most expressed genes (more than 1000 fold increase compared to mock) in response to *E. turcicum* during the incompatible interaction at 72 hai. Although these genes were DE during the compatible and incompatible interaction, their expression was significantly lower during the compatible interaction at the later time point.

### Hundreds of DEGs Were Specific to the Incompatible Interaction at 72 hai in Both Hosts

A total of 263 genes and 439 genes were unique to the incompatible interaction at 72 hai, in maize and sorghum, respectively (Figure 3). Interesting genes related to defense and unique to the incompatible interaction in each crop are highlighted in Tables 2, 3; all DEGs unique to the incompatible interaction at 72 hai in each crop are presented in Supplementary Tables 10, 11. In both crops, we observed DEGs encoding receptor protein kinases, wall-associated kinases, disease-resistance family proteins, hormone related proteins, transporters and transcription factors specific to the incompatible interaction at 72 hai.

**TABLE 2:** Differentially expressed genes related to defense and unique to the incompatible interaction at 72 hai in maize with fold change greater than 15 and FDR < 0.05.

Maize genes		Fold change	FDR	Product^*a*^
ENTREZ ID	Gramene Id			
LOC100191617	GRMZM2G372068	144.1	0.0385	Hydroquinone glucosyltransferase
LOC100274269	GRMZM2G127251	138.7	0.0058	Hydroxycinnamoyltransferase3
LOC103654549	GRMZM2G036365	114.6	0.0133	Aspartic proteinase nepenthesin-1
pco103560(319)	GRMZM2G039639	109.3	0.0122	Protein P21
umc2600	GRMZM2G170017	65.7	0.0175	(+)-Neomenthol dehydrogenase
LOC100502520	GRMZM2G093826	50.4	0.0099	Potassium high-affinity transporter
LOC103636584	GRMZM2G440003	46.5	0.0153	Salicylic acid-binding protein 2
sip1	GRMZM2G374971	44.4	0.0093	Stress-induced protein 1
LOC103654463	GRMZM2G301148	35.2	0.0028	UDP-glycosyltransferase 89B2
LOC103633335	GRMZM2G443843	33.3	0.0118	L-type lectin-domain containing receptor kinase IV.1
LOC103626437	GRMZM2G050450	33.1	0.0169	Benzyl alcohol O-benzoyltransferase
LOC103638616	GRMZM2G340177	33.0	0.018	Putative transcription factor bHLH041
LOC103651266	GRMZM2G101405	32.2	0.0419	probable WRKY transcription factor 51
LOC109943001	no_grmzm_overlap	31.0	0.0453	Putative F-box/LRR-repeat protein 23
LOC100281430	GRMZM2G169966	29.5	0.0135	WRKY70 – superfamily of TFs having WRKY and zinc finger domains
LOC103629700	AC209050.3_FG003	27.1	0.0196	WRKY transcription factor WRKY28
LOC100285450	GRMZM2G074611	26.1	0.0337	Dirigent
LOC100191720	GRMZM2G099297	25.3	0.0331	5-pentadecatrienyl resorcinol O-methyltransferase
LOC103647804	GRMZM2G079082	24.7	0.0329	Putative disease resistance RPP13-like protein 1
LOC109939432	no_grmzm_overlap	22.7	0.0239	Auxin-responsive protein SAUR40
LOC100279719	GRMZM2G073884	22.7	0.0257	Putative leucine-rich repeat receptor-like protein kinase family protein, transcript variant X1
LOC103634934	GRMZM2G333582	22.1	0.0266	Putative transcription factor bHLH041
LOC103638821	GRMZM2G111711	22.1	0.0218	WRKY transcription factor WRKY28
LOC103641905	GRMZM2G059012	19.9	0.0177	Wall-associated receptor kinase 5, transcript variant X2
LOC103641623	GRMZM2G014022	19.9	0.0266	Probable F-box protein At2g36090
LOC103629079	GRMZM5G856011	19.6	0.0331	Cysteine-rich receptor-like protein kinase 6
LOC100282558	no_grmzm_overlap	19.4	0.0068	SAUR20 – auxin-responsive SAUR family member
LOC103633265	GRMZM2G379780	15.8	0.0124	Cysteine-rich receptor-like protein kinase 10
LOC103648003	GRMZM2G106560	15.2	0.0387	Probable WRKY transcription factor 45

*^a^Annotation of maize genes are obtained from NCBI database.*

**TABLE 3:** Differentially expressed genes related to defense and unique to the incompatible interaction at 72 hai in sorghum with fold change greater than 15 and FDR < 0.05.

Sorghum genes	Fold change	FDR	Description^*a*^	Arabidopsis annotation^*b*^
Sobic.005G024100	90.32	0.002	NA^*c*^	Rhamnogalacturonate lyase family protein
Sobic.001G119100	82.35	0.001	Similar to plastocyanin-like domain, putative	Plantacyanin
Sobic.006G005500	70.75	0.026	Predicted protein	Zinc finger (C3HC4-type RING finger) family protein
Sobic.005G219000	66.82	0.034	NA	PATATIN-like protein 4
Sobic.003G164800	48.91	0.028	Similar to Glutathione S-transferase GST 8	Glutathione S-transferase family protein
Sobic.007G198000	41.13	0.021	Similar to Os08g0524400 protein	Auxin-responsive family protein
Sobic.001G381300	36.53	0.005	Weakly similar to WRKY DNA binding domain containing protein	WRKY DNA-binding protein 70
Sobic.001G318200	34.39	0.025	Similar to Putative uncharacterized protein	Glutathione S-transferase TAU 18
Sobic.002G327900	29.38	0.043	Similar to Putative uncharacterized protein	O-Glycosyl hydrolases family 17 protein
Sobic.008G055100	28.12	0.008	Similar to dirigent-like protein, expressed	Disease resistance-responsive (dirigent-like protein) family protein
Sobic.007G214300	24.59	0.007	NA	wall associated kinase 5
Sobic.005G065000	24.07	0.037	Similar to putative uncharacterized protein	Leucine-rich repeat protein kinase family protein
Sobic.008G029300	23.99	0.015	Similar to OSIGBa0147B06.5 protein	NA
Sobic.006G024200	23.14	0.017	Similar to H0512B01.12 protein	Receptor-like protein kinase 1
Sobic.008G099300	22.98	0.006	NA	Cysteine-rich RLK (RECEPTOR-like protein kinase) 41
Sobic.001G164900	22	0.012	NA	UDP-Glycosyltransferase superfamily protein
Sobic.006G113800	21.78	0.017	Similar to OSJNBa0016O02.17 protein	Heavy metal transport/detoxification superfamily protein
Sobic.010G162000	20.97	0.041	Similar to Putative uncharacterized protein	Peroxidase superfamily protein
Sobic.006G061300	19.62	0.039	Similar to High-affinity potassium transporter	High affinity K + transporter 5
Sobic.003G039700	18.21	0.037	Similar to Putative uncharacterized protein	2-oxoglutarate (2OG) and Fe(II)-dependent oxygenase superfamily protein
Sobic.010G061300	17.7	0.021	NA	Leucine-rich repeat protein kinase family protein
Sobic.006G148500	17.53	0.02	NA	Wall associated kinase 5
Sobic.003G210601	17.04	0.009	NA	Disease resistance family protein / LRR family protein
Sobic.007G215700	16.36	0.004	NA	Wall-associated kinase family protein
Sobic.010G054400	16.33	0.017	Similar to Putative uncharacterized protein OJ1606_D04.114	Leucine-rich repeat protein kinase family protein
Sobic.003G312300	16.08	0.015	NA	Homocysteine methyltransferase 2
Sobic.009G171600	15.87	0.011	Similar to WRKY transcription factor 70	WRKY DNA-binding protein 33
Sobic.002G249600	15.45	0.038	NA	Wall-associated kinase 2
Sobic.007G215500	15.12	0.018	Similar to Putative wall-associated serine/threonine kinase	Wall associated kinase 5

*^a^Sorghum annotations are obtained from phytozome database. ^b^Arabidopsis annotation represents the annotation of Arabidopsis orthologs and are used to infer about the unannotated maize and sorghum orthologs. ^c^NA indicates “not available.”*

### Identification of Functionally Related Co-expressed Modules

A gene co-expression network analysis was performed to identify groups of DEGs (modules) with related co-expression in response to *E. turcicum* in maize and sorghum. After filtering the genes with less than one CPM in more than three samples, 22,684 maize genes and 17,731 sorghum genes were retained for gene co-expression network analysis.

**FIGURE 4:**
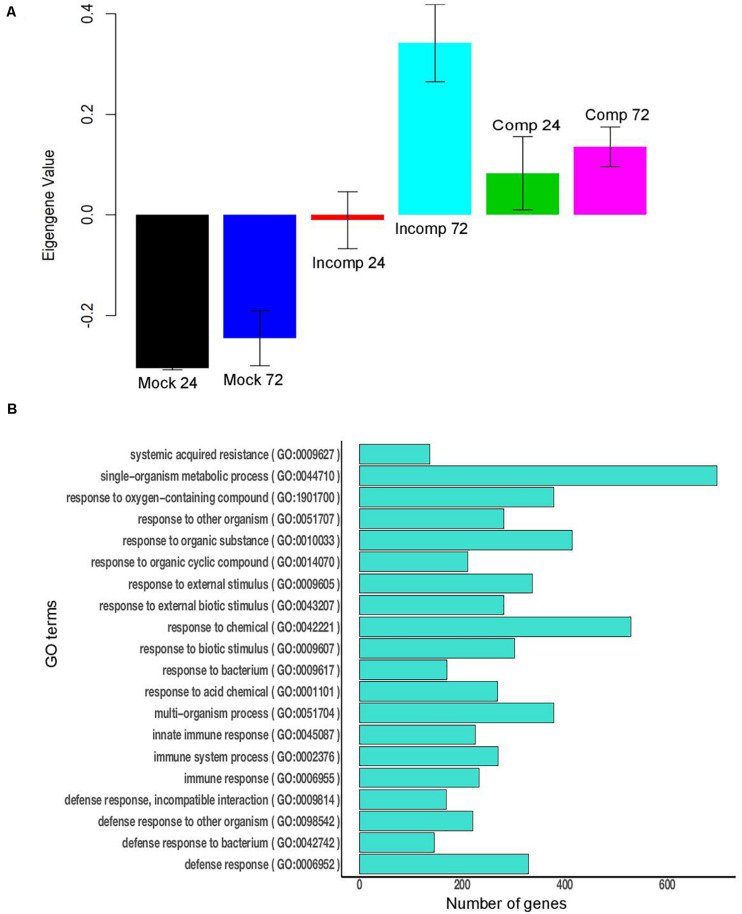
Module 6 of maize contains 1,277 genes. **(A)** Bar plot of module eigengene expression values for different groups. “Mock 24” indicates mock at 24 h after inoculation (hai), “Mock 72” indicates mock at 72 hai, “Incomp 24” indicates incompatible interaction at 24 hai, “Incomp 72” indicates incompatible interaction at 72 hai, “Comp 24” indicates compatible interaction at 24 hai, and “Comp 72” indicates compatible interaction at 72 hai. Error bars indicate standard deviations of the mean of three biological replicates. **(B)** The 20 highly significant GO terms (FDR < 0.05) enriched in module 6 of maize.

The WGCNA analysis identified 58 modules in maize and 33 modules in sorghum. Of the 58 modules in maize, nine modules were significant for the incompatible interaction at 72 hai (6 downregulated and 3 upregulated), and one module was upregulated during the compatible interaction at 24 and 72 hai (Supplementary Table 12). The upregulated modules during the incompatible interaction at 72 hai were modules 6, 40, and 50 (Supplementary Table 13). Module 6 was significantly upregulated in the compatible interaction at 24 and 72 hai. For module 6, the expression of genes, as summarized by ME, was higher for the incompatible interaction at 72 hai compared to compatible interactions (Figure 4A). Module 6 included the largest number of genes (1,277) among upregulated modules and was enriched with several defense-related GO terms that included systemic acquired resistance, response to stress, response to stimulus, response to fungus, response to chemical, response to bacterium, defense response, immune response and innate immune system process (Figure 4B). Modules 40 and 50 were specific to the incompatible interaction at 72 hai in maize, but more defense-related genes were present in module 50. Module 50 was enriched with biological processes such as response to stress, response to stimuli, and regulation of response to stimuli, cell communication, and signaling (Supplementary Figure 3). The downregulated modules in maize contained mostly uncharacterized genes and genes not related to the defense (Supplementary File 1). In summary, module 6 contained the largest number of co-expressed genes related to defense and module 50 contained co-expressed genes related to defense specific to the incompatible interaction at 72 hai in maize.

**FIGURE 5:**
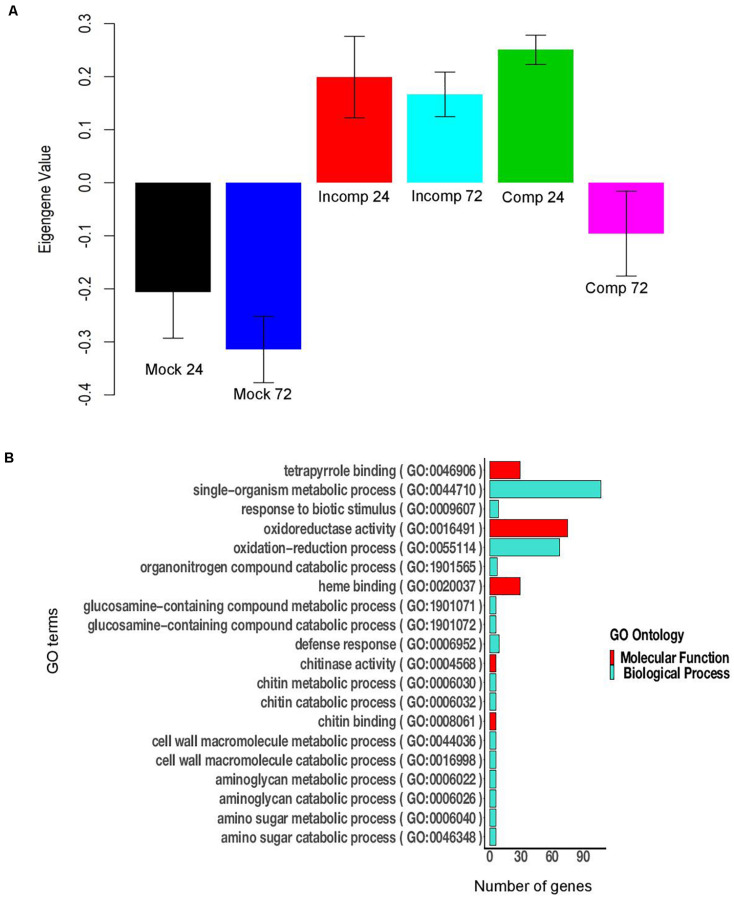
Module 11 of sorghum contains 676 genes. **(A)** Bar plot of module eigengene expression values for different groups. “Mock 24” indicates mock at 24 h after inoculation (hai), “Mock 72” indicates mock at 72 hai, “Incomp 24” indicates incompatible interaction at 24 hai, “Incomp 72” indicates incompatible interaction at 72 hai, “Comp 24” indicates compatible interaction at 24 hai, and “Comp 72” indicates compatible interaction at 72 hai. Error bars indicate standard deviations of the mean of three biological replicates. **(B)** The 20 significant GO terms (FDR < 0.05) enriched in module 11 of sorghum.

In sorghum 11 modules (5 downregulated and 6 upregulated) were associated with the compatible interaction at 24 hai and 12 modules (6 upregulated and 6 downregulated) with the incompatible interaction at 72 hai (Supplementary Table 12). Three downregulated (module 1, module 14, and module 24) and three upregulated modules (module 8, module 11, and module 19) were common between the compatible interaction at 24 hai and the incompatible interaction at 72 hai (Supplementary Table 13). For module 11, the expression of genes, as summarized by ME, was higher for the compatible interaction at 24 hai compared to incompatible interaction at 72 hai (Figure 5A). Module 11 (676 genes) was enriched with several defense related GO terms that included defense response, response to biotic stimulus, oxidoreductase activity, chitin binding, chitinase activity, o-methyltransferase activity, oxidoreductase activity, peroxidase activity, and hydrolase activity (Figure 5B). The upregulated modules 12, 31, and 32 were specific to the incompatible interaction at 72 hai (Supplementary Table 13). Module 12 was enriched in defense-related terms such as response to biotic stimulus, oxidation-reduction process, defense response, o-methyltransferase activity, peroxidase activity, chitinase activity, amino-sugar metabolic process (Supplementary Figure 4). The downregulated modules mostly contained uncharacterized genes and genes not related to the defense (Supplementary File 1). In summary, module 11 was enriched in co-expressed genes related to defense against *E. turcicum* and module 12 contained co-expressed genes related to defense specific to the incompatible interaction at 72 hai in sorghum. Overall, we identified modules containing defense-related genes in maize and sorghum that were used to identify hub genes involved in leaf blight resistance in both crops.

### Hub Genes Are Potential Core Regulators of Defense in Response to *E. turcicum* in Maize and Sorghum

**FIGURE 6:**
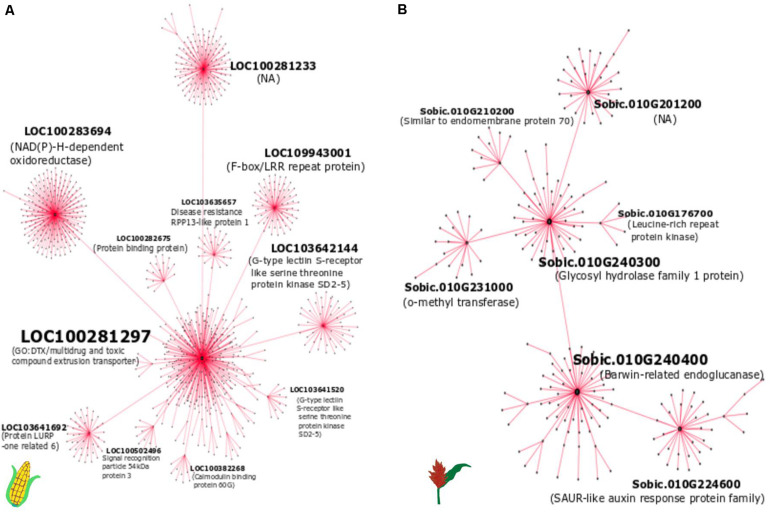
The weighted gene co-expression network of **(A)** maize module 6 and **(B)** sorghum module 11. The hub genes (present at the center) that are connected to other genes in each sub-network are labeled.

We selected module 6 of maize and module 11 of sorghum to construct the gene expression network, as these modules were enriched with defense-related genes. The hub genes in the significant modules containing defense related co-expressed genes were identified based on node degree (Figure 6) and MM (Table 4) in both crops. Although we found conserved gene expression in maize and sorghum, we did not find an overlap in hub genes between the two crops. However, some shared DEOs, including genes encoding a barwin-related endoglucanse, a basic helix-loop-helix (bHLH) DNA-binding family protein, an o-methyltransferase family protein, and a G-type lectin S-receptor-like serine threonine protein kinase, were identified as hub genes in either maize or sorghum. A hub gene in maize (*LOC103648077*) based on MM was found to encode an LRR receptor-like serine/threonine-protein kinase FLS2 (Table 4). The hub genes (Table 4 and Figure 6) are related to defense and likely play a key role in the interaction with *E. turcicum*.

**TABLE 4:** Hub genes in maize module 6 and sorghum module 11 based on module membership (MM) and gene significance.

Crop	Gene^*a*^	Description^*b*^	Module	MM	Gene significance	
					Incompatible 72 hai	Compatible 24 hai
Maize	LOC100382268 (GRMZM2G176472)	Calmodulin-binding protein 60 G, transcript variant X1	Module 6	0.97	5.98	4.45
Maize	bHLH94 (GRMZM5G849600)	Putative HLH DNA-binding domain superfamily protein	Module 6	0.94	6.82	6.82
Maize	LOC103640383 (GRMZM2G081458)	Putative disease resistance protein RGA3	Module 6	0.97	6.29	1.29
Maize	LOC103648077 (GRMZM2G372058)	LRR receptor-like serine/threonine-protein kinase FLS2	Module 6	0.93	5.5	5.6
Maize	LOC103627130 (GRMZM2G325023)	UDP-glycosyltransferase 73D1-like	Module 6	0.9	6.18	4.94
Maize	geb1 (GRMZM2G065585)	Glucan endo-1,3-beta-glucosidase homolog 1	Module 6	0.89	6.01	5.36
Sorghum	Sobic.005G126200	Similar to leucine-rich repeat-containing extracellular glycoprotein precursor	Module 11	0.9	7.9	9
Sorghum	Sobic.008G182700	Similar to thaumatin-like pathogenesis-related protein 4 precursor	Module 11	0.9	7.5	8.1
Sorghum	Sobic.008G182600	Similar to pathogenesis related protein-5	Module 11	0.9	7.2	9
Sorghum	Sobic.008G182400	Similar to thaumatin-like protein, putative, expressed	Module 11	0.9	5.6	7.4

*^a^Maize genes are based on NCBI Id and the gramene Id (Id inside the bracket). Sorghum genes are based on phytozome v12 Id. ^b^Description of maize genes are based on NCBI database and description of sorghum genes are based on phytozome database.*

## Discussion

This is the first systematic comparison of the defense transcriptome in maize and sorghum and was used to understand the relationship between host resistance in the two crops. We explored compatible and incompatible transcriptional responses in maize and sorghum and found strong similarities in the host resistance responses of both crops, particularly in the incompatible interactions. Genes related to QDR were critical for resistance, including for the incompatible response. The defense-related genes with conserved expression in the two grasses may be evolutionarily important and involved in disease resistance to other pathogens and other crops in the Andropogoneae tribe. The lineage-specific transcriptional response provides insights into the evolution of maize and sorghum immunity to a common pathogen. Regulatory divergent evolution, shaped by both *cis*- and *trans*-regulatory variance, has played a role in host resistance in maize and sorghum to this pathogen. Our results are important for developing resistance to this critical pathogen and understanding the evolution of host-pathogen interactions.

Based on pathogen diversity, it has been hypothesized that *E. turcicum* co-evolved with maize in the Americas and subsequently moved to Africa (Borchardt et al., 1998). We hypothesized that because maize has been a host of *E. turcicum* longer, that the transcriptional response of maize would involve more genes. Over many selection cycles, the host response becomes more diversified and fine-tuned, and thus more genes become involved in the response to the pathogen (Dangl and Jones, 2001; Jones and Dangl, 2006). Our data support this hypothesis, as there were both a greater total number of genes expressed in maize and more co-expression modules in maize. It is important to note that only one maize and sorghum line were used in this study and to examine this hypothesis further, more lines would need to be examined. The large number of genes involved in the pathogen response in both species is supportive of the infinitesimal model of plant QDR, where resistance is controlled by a large number of loci and each makes a very small contribution to the phenotype (Fisher, 1919; Nelson et al., 2013).

The expressed genes and DEGs in each maize and sorghum were enriched in core orthologs, but only a fraction of core gene ortholog pairs were DE in both crops, and there was no overlap in hub genes between the two crops. The enrichment of expressed core genes is consistent with that of abiotic stress responses, where more than 50% of total coding genes were identified as syntenic orthologs in each maize and sorghum (Zhang et al., 2017). This indicates that while the coding sequences have been conserved, their function, and likely transcriptional regulation have not. *Trans*-regulatory differences often contribute greater variation within species and *cis*-regulatory differences contribute greater divergence between species, which might be due to large deleterious pleiotropic effects associated with *trans*-factors (Signor and Nuzhdin, 2018). In *Drosophila*, *cis*-regulatory variations accounted for larger gene expression differences between species compared to within species (Wittkopp et al., 2008). We hypothesize that *cis*-regulation is responsible for the greater proportion of lineage-specific gene expression in our study, however, the possibility of *trans*-regulatory variations cannot be ruled out, and further investigation is required to address this hypothesis. The neofunctionalization model predicts that after gene duplication one duplicate copy undergoes faster and positive evolution with potentially novel functions (Zou et al., 2009). A transcriptional analysis of six species of the Pentapetalae with quantitative resistance to *Sclerotinia sclerotiorum* indicated that regulatory divergence contributed to the evolution of QDR in these species (Sucher et al., 2020). In our study, the lack of overlap in DEGs and shared hub genes between the two crops indicate that regulatory mutations and neofunctionalization may be playing a role in the evolution of host resistance; whereas, the genes with conserved expression in both crops might be the result of parallel evolution (Pickersgill, 2018).

Maize is an ancient tetraploid and the duplicated genes in the maize genome have likely undergone neofunctionalization developing new functions, including those relevant to stress responses. We observed more contributions from the maize1 subgenome to the total transcriptional response to *E. turcicum* compared to the maize2 subgenome. This is consistent with previous reports of higher gene expression in the maize1 subgenome over a range of experiments quantifying RNA abundance in different tissues, such as shoots, roots, leaf tip, leaf base, mesophyll, bundle sheath, and shoot apical meristem (Schnable et al., 2011), and in response to abiotic stress (Zhang et al., 2017). The dominance of one subgenome in maize, along with other paleo-allopolyploids such as *Arabidopsis thaliana* and *Brassica* sp., has been associated with the lower transposon element densities near genes in the dominant subgenome (Alger and Edger, 2020). Our data confirms that the genes from maize1 subgenome have higher expression in response to biotic stress as well.

We observed more DEGs for the incompatible interaction at 72 hai and compatible interaction at 24 hai. One possible explanation for this is that the pathogen takes a longer time to penetrate and trigger a defense response in the incompatible interaction compared to that of compatible interaction, and so fewer transcriptional changes are observed at 24 hai in the incompatible interaction. In the hemibiotrophic fungal *Magnaporthe oryzae-*rice pathosystem (rice blast), fungal spores of *M. oryzae* germinated, formed infection-specific appressoria, and invaded epidermal cells within 24 hai, suggesting 24 hai as a critical time point for pathogen invasion in the compatible interaction (Talbot, 2003; Wei et al., 2013). In the compatible interaction at 72 hai the pathogen suppressed host defenses, but in the incompatible interaction the defense response was heightened as compared to the compatible interaction. Also, expression was more conserved during the incompatible interaction at 72 hai (∼16% of the core genes) between the two crops, indicating common defense mechanisms to combat the pathogen during the incompatible interaction at the later time point. These results suggest that genes expressed at 72 hai are important for incompatible interaction defense mechanisms in both maize and sorghum.

We observed a more robust transcriptional response in the incompatible interactions with larger fold change differences at the later time point in both crops. Previously, similar transcriptomic profiles with quantitative differences were reported between the compatible and incompatible interaction in the *Arabidopsis thaliana*–*Pseudomonas syringae* interaction (Tao et al., 2003) and the rice–*M. oryzae* interaction (Wei et al., 2013). The similar expression profile but higher fold changes of genes in the incompatible compared to the compatible interaction at 72 hai in our study suggests that common QDR genes are involved in both the compatible and incompatible interaction, but with stronger induction in the incompatible interaction.

Quantitative resistance is the most common form of resistance to *E. turcicum* (Wisser et al., 2006; Galiano-Carneiro and Miedaner, 2017), and the genes we identified as being DE and conserved, including in the incompatible interaction, are consistent with QDR-related mechanisms. Genes related to quantitative resistance can be broadly categorized into perception, signal transduction, and defense response (Corwin and Kliebenstein, 2017). Many of the DEGs, including the conserved genes, were involved in the defense response, which is consistent with previous studies where the defense response is largely responsible for QDR (Chung et al., 2010; Kump et al., 2011). Signal transduction is the second most prevalent underlying mechanism of QDR (Corwin and Kliebenstein, 2017), and we identified several transcription factors and other signaling-related genes in our study.

Several gene families conserved between the two crops in our study are involved in signal perception and transduction, including wall-associated receptor-like kinases (WAK) and S-domain receptor kinases (SRK). WAKs have been implicated in major gene resistance in maize to several pathogens including *E. turcicum* (Hurni et al., 2015; Zuo et al., 2015). The gene *Sobic.002G249600*, encoding the wall-associated kinase 2, was significantly associated with SLB resistance (Zhang et al., 2020) and significantly upregulated in the incompatible interaction at 72 hai in the current study. SRK are involved in self-incompatibility (Zou et al., 2015), biotic stress (Pastuglia et al., 1997) and abiotic stress (Zou et al., 2015). An S-locus lectin protein kinase encoding gene (*Sobic.006G228200*) was significant in a GWAS for SLB resistance in sorghum (Zhang et al., 2020) and also upregulated in response to *E. turcicum* in the current study. Some disease resistance genes, such as *Pto* in tomato (Martin et al., 1993) and *Xa-21* in rice (Song et al., 1995), encode serine/threonine protein kinases. The *Lr10* resistance gene in wheat (Feuillet et al., 1997) belongs to the same gene family as the S-locus receptor kinase. Together these results suggest that perception and signal transduction are an important components of QDR to *E. turcicum*, and QDR is implicated in the incompatible reaction.

Perception of microbes, either directly or indirectly, is a first line of defense against pathogens and a component of QDR (Cole and Diener, 2013; Roux et al., 2014; Corwin et al., 2016; Corwin and Kliebenstein, 2017). A wide range of receptor-like kinases were conserved between species during the incompatible interaction at 72 hai, indicating that perception is conserved and is important for resistance in the incompatible interaction.

PAMP-triggered immunity has been hypothesized to be related to QDR and non-host resistance (Lipka et al., 2008; Poland et al., 2009), so we examined whether there was any relationship with PTI in the *E. turcicum* interactions. PTI-related mechanisms were implicated. We identified a gene *LOC103648077* encoding an LRR receptor-like serine/threonine-protein kinase FLS2 as a hub gene in maize module 6 (Supplementary Table 8), suggesting a role for PTI. PTI has been implicated in QDR (Bhattarai et al., 2016). In sorghum, pretreatment with the PAMPs flg22 and chitooctaose activated PTI and reduced lesion sizes following *Herbaspirillum rubrisubalbicans* inoculation, and genes encoding receptor kinases and PR proteins were DE after *H. rubrisubalbicans* inoculation (Tuleski et al., 2020). Overall, these data highlight PTI as a common defense mechanism in maize and sorghum during both compatible and incompatible interactions.

It has been hypothesized that there are many possible molecular mechanisms underlying QDR (Poland et al., 2009). The DEGs specific to the incompatible interaction at 72 hai, such as hydroxycinnamoyltransferase (HCT) genes in maize and potassium transporters in both maize and sorghum reveal potential resistance mechanisms. In maize, HCT genes are involved in the biosynthesis of lignin and two HCT genes (HCT1806 and HCT4918) were identified as NLR cofactors that negatively regulate Rp1-D21 induced HR through physical interaction between the proteins (Wang et al., 2015). In sorghum, a gene encoding HCT1 was repressed in response to bacterial infection (Tuleski et al., 2020). In transgenic tomato, overexpressing the genes encoding tyramine HCT showed enhanced resistance in response to *P. syringae* (Campos et al., 2014). Likewise, a potassium transporter was implicated in rice blast disease resistance, where an increased level of potassium enhanced resistance to the hemibiotrophic fungal pathogen *M. oryzae* (Shi et al., 2018). Genes specific to the incompatible interaction are strong candidates for conferring resistance to *E. turcicum* and may provide the basis for an incompatible response.

Sugar transporters are an important class of genes that have a conserved role in plant–pathogen interactions in multiple species. Sugar transporters have been reported both as targets for pathogen manipulation to access carbohydrates, such as the SWEET transporter *OsSWEET14* in rice (Li et al., 2012) and *Lr67* in wheat (Moore et al., 2015), and a layer of plant defense, such as *AtSTP13* in Arabidopsis (Yamada et al., 2016). Consistent with our previous GWAS study (Zhang et al., 2020), the gene *Sobic.001G469500*, which encodes a putative sugar transporter, was also upregulated in response to *E. turcicum* during the compatible interaction in the current study. Therefore, sugar transporters might be conferring susceptibility to *E. turcicum*.

## Conclusion

For this pathosystem there are key components of the resistance response that are shared across both hosts. There were more DEGs during the incompatible interaction at 72 hai and the compatible interaction at 24 hai in both crops. The gene expression patterns were similar but differed quantitatively between compatible and incompatible interactions. Of the DEGs with conserved expression during the incompatible interaction at 72 hai, several were related to QDR and to a lesser extent PTI, highlighting their importance in conferring a robust and complete resistance response to *E. turcicum*. We identified several candidate genes that could be useful moving forward, including genes for which natural variation exists and that are DE. This study also provides an enhanced understanding of how different host species deploy their defense system in response to a common pathogen.

## Data Availability Statement

The datasets presented in this study can be found in online repositories. The names of the repository/repositories and accession number(s) can be found below: https://www.ncbi.nlm.nih.gov/geo/query/acc.cgi?acc=GSE156026.

## Author Contributions

TJ and SM conceived the idea. PA designed the experiment, generated the data, and analyzed the data. PA and TJ interpreted the data and wrote the manuscript. PA, TJ, and SM revised the manuscript. All authors read and approved the final manuscript.

## Conflict of Interest

The authors declare that the research was conducted in the absence of any commercial or financial relationships that could be construed as a potential conflict of interest.

## Footnotes

1 https://www.idtdna.com/PrimerQuest/Home/Index

## Supplementary Material

Supplementary Figure 1 The compatible and incompatible interaction of maize and sorghum with *Exserohilum turcicum* at 72 h after inoculation.

Supplementary Figure 2 Read fates per sample of maize (A) and sorghum (B). “M” and “S” indicate maize and sorghum, respectively. “Mo,” “Mst,” and “Sst” indicate mock, maize-specific isolate, and sorghum specific isolate, respectively. T1 and T2 represent sample collected 24 h after inoculation (hai) and 72 hai, respectively. The number 1, 2, and 3 represent three biological replicates.

Supplementary Figure 3 Comparisons of fold change between mock and treatment groups obtained from RNA-sequencing and qRT-PCR for maize gene PR-5 (A), sorghum genes Sobic.005G101500 (B), and Sobic.001G020200 (C).

Supplementary Figure 4 The 20 highly significant GO terms (FDR < 0.05) enriched in (A) maize module 50 and (B) sorghum module 12. Both modules are only significant on the incompatible interaction at 72 hai.

Supplementary Table 1 Primers and probes information used in qRT-PCR.

Supplementary Table 2 Number of differentially expressed genes (FDR < 0.05 and FC > 2) in maize and sorghum in response to *Exserohilum turcicum.*

Supplementary Table 3 Summary of significant GO terms enriched during four interaction-time combinations in maize and sorghum.

Supplementary Table 4 The GO terms significantly enriched during the incompatible interaction at 72 hai in maize.

Supplementary Table 5 The GO terms significantly enriched during incompatible interaction at 72 hai in sorghum.

Supplementary Table 6 Significantly enriched GO terms common to maize and sorghum during the incompatible interaction at 72 hai.

Supplementary Table 7 Conserved orthologs differentially regulated in both maize and sorghum (shared DEOs).

Supplementary Table 8 The common genes that are differentially expressed in all interaction-time combinations with their respective fold change compared to mock in maize.

Supplementary Table 9 The common genes that are differentially expressed in all interaction-time combinations with their respective fold change compared to mock in sorghum.

Supplementary Table 10 The DEGs that are unique to incompatible interaction at 72 hai in maize.

Supplementary Table 11 The DEGs that are unique to incompatible interaction 72 hai in sorghum.

Supplementary Table 12 Number of differentially expressed modules identified by weighted correlation network analysis (WGCNA) in maize and sorghum in response to *Exserohilum turcicum.*

Supplementary Table 13 Summary of modules that are significantly associated (FDR < 0.05) with at least one of the four interaction-time combinations in each maize and sorghum.

Supplementary File 1 Summary of downregulated modules in each maize and sorghum.

## Abbreviations

NCLB: northern corn leaf blight
SLB: sorghum leaf blight
GWAS: genome wide association study
DEGs: differentially expressed genes
GO: gene ontology
DEOs: differentially expressed orthologs
PAMP: pathogen associated molecular pattern
PTI: PAMP-triggered immunity
QDR: quantitative disease resistance
PR: pathogenesis-related
CPM: count per million
WGCNA: weighted correlation network analysis
WAK: wall-associated receptor-like kinases
SRK: S-domain receptor kinases
FDR: false discovery rate
SEA: singular enrichment analysis
TMM: trimmed mean of M-values
ME: module eigengene
MM: module membership
GS: gene significance
kME: eigengene based connectivity.
