# Supplementary material for: Differential Regulation of Maize and Sorghum Orthologs in Response to the Fungal Pathogen Exserohilum turcicum
Source: Front Plant Sci. 2021 May 25;12:675208. doi: 10.3389/fpls.2021.675208 (PMC8185347; doi:10.3389/fpls.2021.675208)
Supplement: Supplementary Figure 1 — The compatible and incompatible interaction of maize and sorghum with Exserohilum turcicum at 72 h after inoculation. [file Data_Sheet_1.zip › Supplementary Figures and Tables.docx]

Supplementary Material

# Supplementary Figures

##
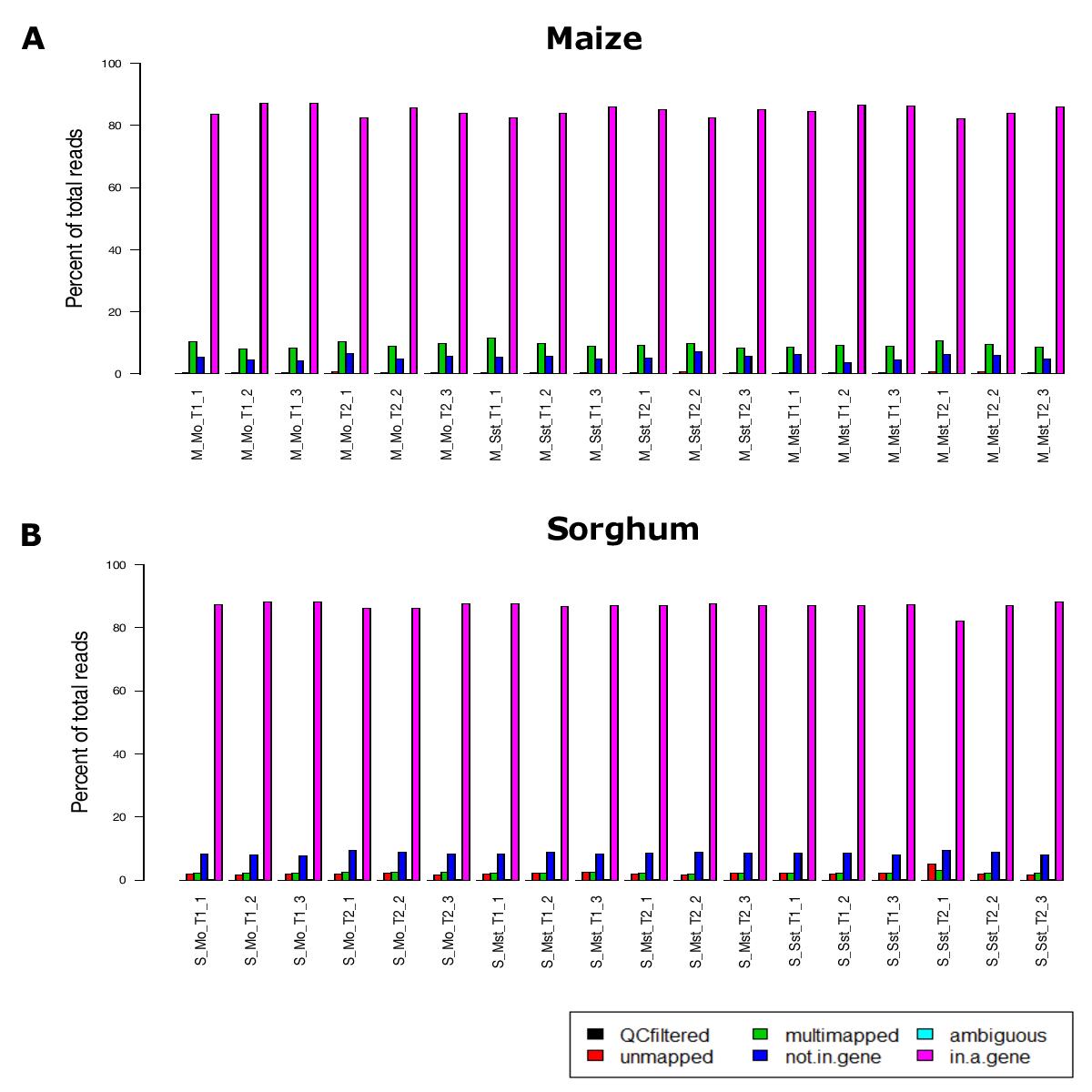


## Supplementary Figure 1. Read fates per sample of maize (A) and sorghum (B). ‘M’ and ‘S’ indicate maize and sorghum respectively. ‘Mo’, ‘Mst’, and ‘Sst’ indicate mock, maize-specific isolate, and sorghum specific isolate respectively. T1 and T2 represent sample collected 24 hours after inoculation (hai) and 72 hai respectively. The number 1, 2, and 3 represent three biological replicates.

##
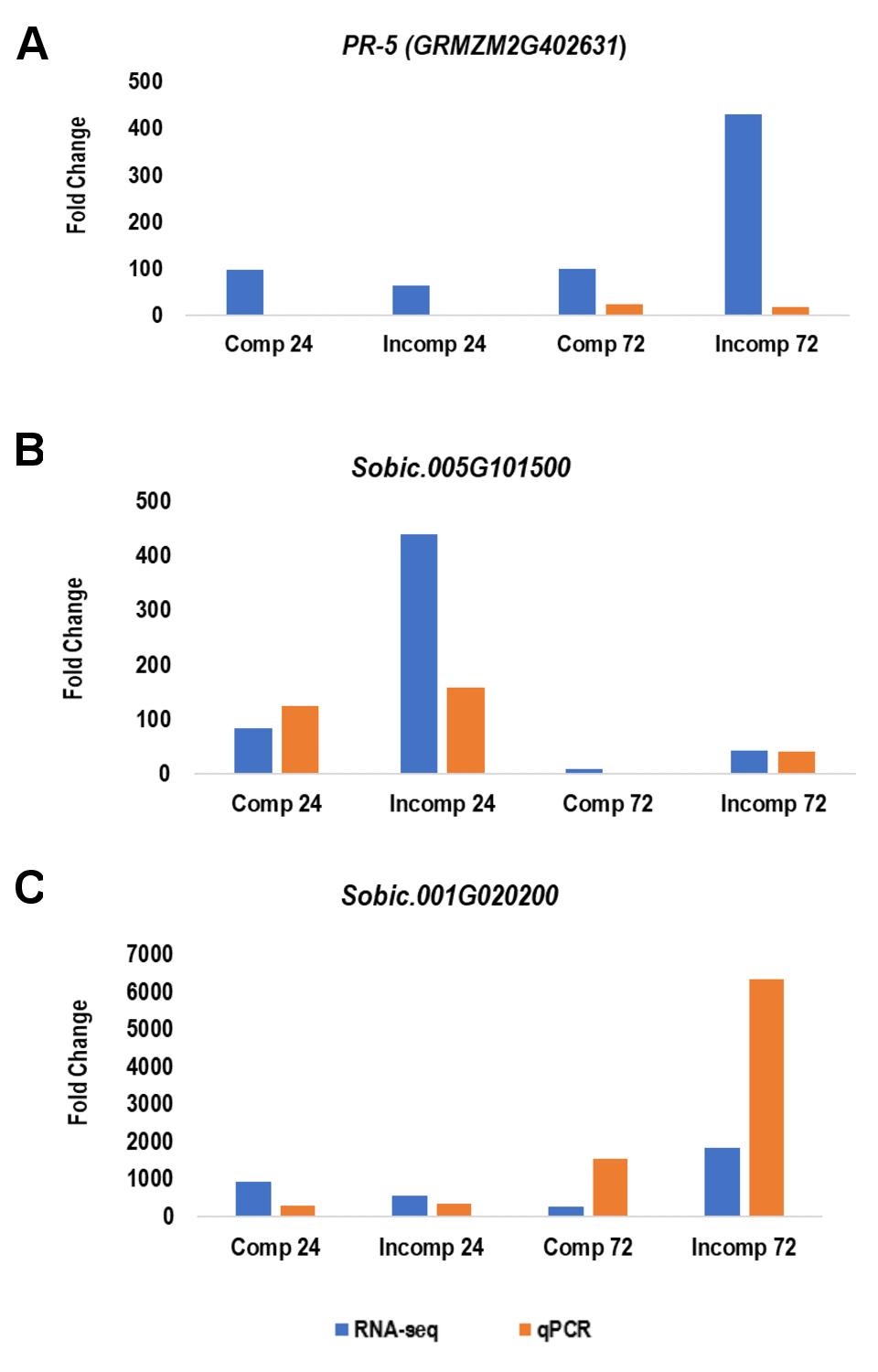


## Supplementary Figure 2. Comparisons of fold change between mock and treatment groups obtained from RNA-sequencing and qRT-PCR for maize gene *PR-5* (A), sorghum genes *Sobic.005G101500* (B) and *Sobic.001G020200* (C).

##
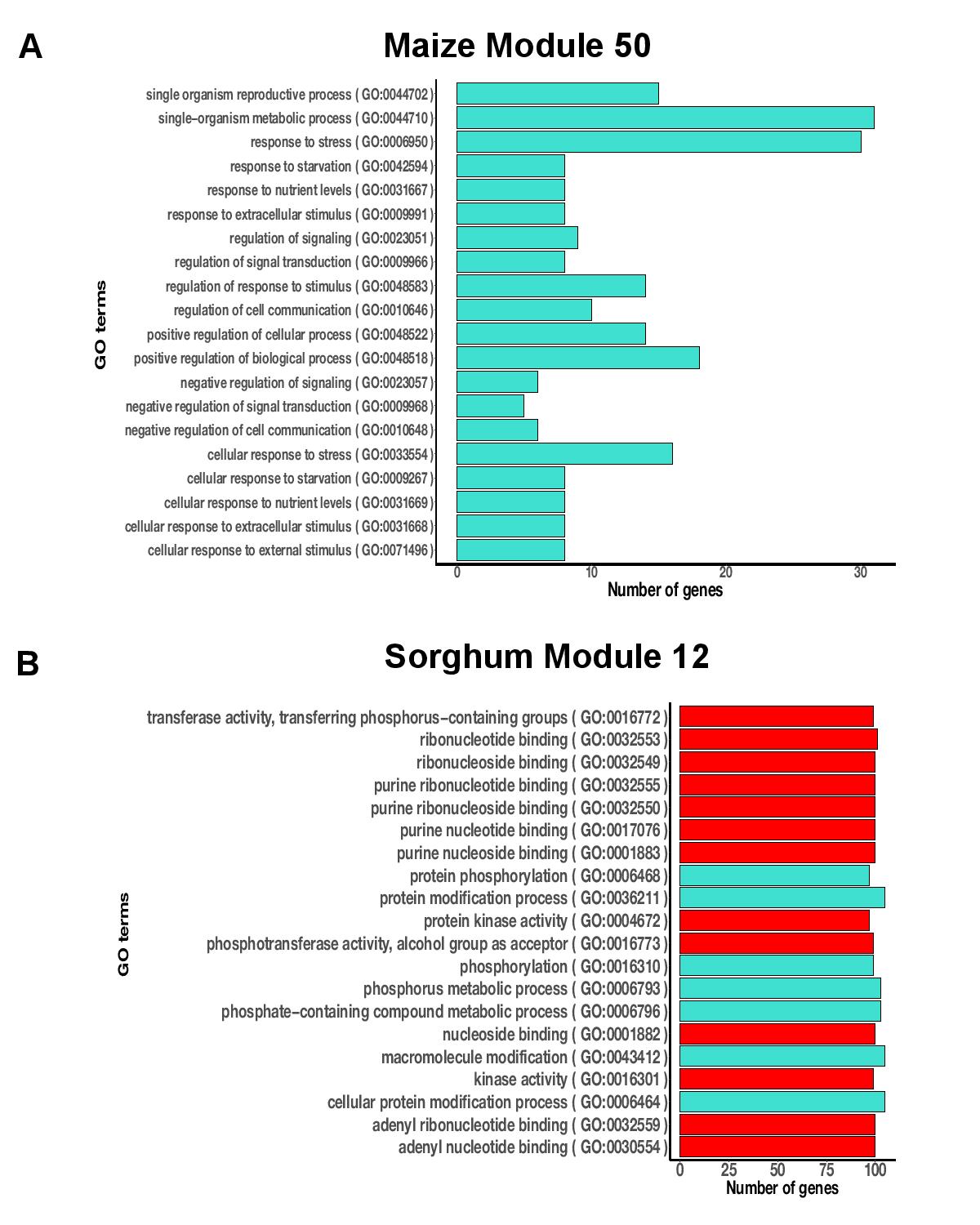


## Supplementary Figure 3. The 20 highly significant GO terms (FDR< 0.05) enriched in A) maize module 50 and B) sorghum module 12. Both modules are only significant on the incompatible interaction at 72 hai.

# Supplementary Tables

# Supplementary Table 1. Primers and probes information used in qRT-PCR.

| **Gene** | **Crop** | **Probe Sequence** | **Forward Primer Sequence** | **Reverse Primer Sequence** |
| --- | --- | --- | --- | --- |
| PR-5  (GRMZM2G402631) | Maize | TGGCGGCAACAGCAACTACCAA | CGACATGAAGACCCATGC | TCACTAGCCCATGCAAATG |
| betatubulin7 | Maize | TCATCACCACTGACCTGCTTGCCC | GAACACCAGGGACATCATC | GACCTGCTGGACATCAATAC |
| Sobic.001G020200 | Sorghum | TTGTGTCCCACGATGTTGCCGC | CGTCTTCATCATCTGCAACTA | GACGTACGTGTCTGTGTAAG |
| Sobic.005G101500 | Sorghum | TTGAACGCAACCGACGGCAACG | ACCGGGTATAGCGAGTTC | GGCTCAGGTACATGTCAATC |
| PP2A | Sorghum | ACCCTGATGTTGCGGATGAGAGGAGC | AACCCGCAAAACCCCAGACTA | TACAGGTCGGGCTCATGGAAC |

# **Supplementary Table 2**. Number of differentially expressed genes (FDR <0.05 and FC > 2) in maize and sorghum in response to *S. turcica.*

| **Crop** | **Interaction** | **Time** | **No. of differentially expressed genes** | | |
| --- | --- | --- | --- | --- | --- |
|  |  |  | **Down** | **Up** | **Sum** |
| Maize | Compatible | 24 hai | 2 | 127 | 129 |
| Maize | Incompatible | 24 hai | 6 | 59 | 65 |
| Maize | Compatible | 72 hai | 1 | 170 | 171 |
| Maize | Incompatible | 72 hai | 51 | 409 | 460 |
| Sorghum | Compatible | 24 hai | 322 | 496 | 818 |
| Sorghum | Incompatible | 24 hai | 47 | 279 | 326 |
| Sorghum | Compatible | 72 hai | 26 | 145 | 171 |
| Sorghum | Incompatible | 72 hai | 171 | 612 | 783 |

# Supplementary Table 3. Summary of significant GO terms enriched during four interaction-time combinations in maize and sorghum.

| **Crop** | **Interaction** | **No. of input genes^a^** | **Annotated number in the input list^b^** | **No. of significant GO terms (FDR <0.05)^c^** | | | |
| --- | --- | --- | --- | --- | --- | --- | --- |
|  |  |  |  | **Total** | **Biological Process** | **Molecular Function** | **Cellular Process** |
| Maize | Compatible 24 | 131 | 120 | 87 | 67 | 5 | 15 |
| Maize | Incompatible 24 | 65 | 63 | 9 | 9 | 0 | 0 |
| Maize | Compatible 72 | 171 | 158 | 164 | 127 | 27 | 10 |
| Maize | Incompatible 72 | 476 | 436 | 312 | 237 | 54 | 21 |
| Sorghum | Compatible 24 | 1096 | 632 | 28 | 17 | 7 | 4 |
| Sorghum | Incompatible 24 | 375 | 242 | 42 | 28 | 10 | 4 |
| Sorghum | Compatible 72 | 205 | 132 | 5 | 2 | 3 | 0 |
| Sorghum | Incompatible 72 | 946 | 576 | 53 | 35 | 18 | 0 |

^a^Number of input DEGs used for GO annotation.

^b^Number of DEGs that were annotated with GO terms among the total input DEGs.

^c^Number of annotated DEGs with significant GO terms.

# Supplementary Table 4. The GO terms significantly enriched during the incompatible interaction at 72 hai in maize.

| **GO term** | **Ontology** | **Description** | **Number in input list** | **Number in BG/Ref** | **FDR** |
| --- | --- | --- | --- | --- | --- |
| GO:0006952 | BP | defense response | 150 | 2453 | 5.90E-20 |
| GO:0009814 | BP | defense response, incompatible interaction | 79 | 998 | 9.40E-17 |
| GO:0009607 | BP | response to biotic stimulus | 137 | 2422 | 3.50E-16 |
| GO:0045087 | BP | innate immune response | 104 | 1599 | 3.50E-16 |
| GO:0009627 | BP | systemic acquired resistance | 66 | 812 | 7.60E-15 |
| GO:0098542 | BP | defense response to other organism | 102 | 1657 | 1.30E-14 |
| GO:0043207 | BP | response to external biotic stimulus | 126 | 2278 | 1.30E-14 |
| GO:0051707 | BP | response to other organism | 126 | 2278 | 1.30E-14 |
| GO:0042742 | BP | defense response to bacterium | 64 | 787 | 1.30E-14 |
| GO:0050832 | BP | defense response to fungus | 53 | 575 | 2.50E-14 |
| GO:0009620 | BP | response to fungus | 64 | 803 | 2.60E-14 |
| GO:0006955 | BP | immune response | 106 | 1799 | 3.10E-14 |
| GO:0009617 | BP | response to bacterium | 72 | 1035 | 3.70E-13 |
| GO:0002376 | BP | immune system process | 118 | 2196 | 4.40E-13 |
| GO:0014070 | BP | response to organic cyclic compound | 93 | 1619 | 4.70E-12 |
| GO:0034050 | BP | host programmed cell death induced by symbiont | 63 | 886 | 4.70E-12 |
| GO:0071229 | BP | cellular response to acid chemical | 82 | 1342 | 4.70E-12 |
| GO:0009626 | BP | plant-type hypersensitive response | 63 | 886 | 4.70E-12 |
| GO:0010363 | BP | regulation of plant-type hypersensitive response | 59 | 809 | 9.20E-12 |
| GO:0001101 | BP | response to acid chemical | 119 | 2365 | 1.70E-11 |
| GO:0009751 | BP | response to salicylic acid | 63 | 923 | 2.40E-11 |
| GO:0050776 | BP | regulation of immune response | 63 | 952 | 8.70E-11 |
| GO:0045088 | BP | regulation of innate immune response | 61 | 906 | 9.00E-11 |
| GO:1901700 | BP | response to oxygen-containing compound | 157 | 3561 | 1.60E-10 |
| GO:0051704 | BP | multi-organism process | 158 | 3610 | 2.30E-10 |
| GO:0009863 | BP | salicylic acid mediated signaling pathway | 51 | 698 | 2.70E-10 |
| GO:0009862 | BP | systemic acquired resistance, salicylic acid mediated signaling pathway | 40 | 463 | 3.40E-10 |
| GO:0080135 | BP | regulation of cellular response to stress | 59 | 902 | 5.30E-10 |
| GO:0071446 | BP | cellular response to salicylic acid stimulus | 51 | 715 | 5.80E-10 |
| GO:0019748 | BP | secondary metabolic process | 78 | 1389 | 6.10E-10 |
| GO:0009719 | BP | response to endogenous stimulus | 142 | 3218 | 9.40E-10 |
| GO:0031347 | BP | regulation of defense response | 64 | 1048 | 1.20E-09 |
| GO:0002682 | BP | regulation of immune system process | 64 | 1056 | 1.60E-09 |
| GO:1901698 | BP | response to nitrogen compound | 81 | 1517 | 2.50E-09 |
| GO:0006468 | BP | protein phosphorylation | 85 | 1633 | 2.90E-09 |
| GO:1901701 | BP | cellular response to oxygen-containing compound | 91 | 1799 | 2.90E-09 |
| GO:0071395 | BP | cellular response to jasmonic acid stimulus | 44 | 599 | 4.50E-09 |
| GO:0010243 | BP | response to organonitrogen compound | 61 | 1011 | 4.50E-09 |
| GO:0009605 | BP | response to external stimulus | 142 | 3311 | 4.60E-09 |
| GO:0080134 | BP | regulation of response to stress | 67 | 1177 | 6.40E-09 |
| GO:0010310 | BP | regulation of hydrogen peroxide metabolic process | 31 | 332 | 8.60E-09 |
| GO:0009725 | BP | response to hormone | 125 | 2841 | 9.40E-09 |
| GO:0009867 | BP | jasmonic acid mediated signaling pathway | 43 | 597 | 1.20E-08 |
| GO:0010200 | BP | response to chitin | 51 | 795 | 1.60E-08 |
| GO:0009755 | BP | hormone-mediated signaling pathway | 81 | 1593 | 1.80E-08 |
| GO:0002679 | BP | respiratory burst involved in defense response | 26 | 257 | 4.30E-08 |
| GO:0009696 | BP | salicylic acid metabolic process | 35 | 443 | 4.70E-08 |
| GO:2000377 | BP | regulation of reactive oxygen species metabolic process | 33 | 405 | 6.30E-08 |
| GO:0010033 | BP | response to organic substance | 171 | 4381 | 6.30E-08 |
| GO:0045730 | BP | respiratory burst | 26 | 263 | 6.60E-08 |
| GO:0043067 | BP | regulation of programmed cell death | 64 | 1177 | 7.40E-08 |
| GO:0006995 | BP | cellular response to nitrogen starvation | 18 | 128 | 9.70E-08 |
| GO:0010941 | BP | regulation of cell death | 65 | 1215 | 1.00E-07 |
| GO:0042743 | BP | hydrogen peroxide metabolic process | 39 | 553 | 1.20E-07 |
| GO:0071495 | BP | cellular response to endogenous stimulus | 87 | 1849 | 1.30E-07 |
| GO:0032870 | BP | cellular response to hormone stimulus | 82 | 1709 | 1.50E-07 |
| GO:0071407 | BP | cellular response to organic cyclic compound | 57 | 1013 | 1.50E-07 |
| GO:0012501 | BP | programmed cell death | 70 | 1399 | 3.50E-07 |
| GO:0072593 | BP | reactive oxygen species metabolic process | 42 | 659 | 5.00E-07 |
| GO:0071310 | BP | cellular response to organic substance | 104 | 2422 | 5.00E-07 |
| GO:0009753 | BP | response to jasmonic acid | 50 | 865 | 5.10E-07 |
| GO:0008219 | BP | cell death | 72 | 1477 | 5.70E-07 |
| GO:0009698 | BP | phenylpropanoid metabolic process | 45 | 743 | 6.50E-07 |
| GO:0031348 | BP | negative regulation of defense response | 24 | 256 | 6.50E-07 |
| GO:0010583 | BP | response to cyclopentenone | 23 | 238 | 7.20E-07 |
| GO:0043562 | BP | cellular response to nitrogen levels | 18 | 148 | 8.10E-07 |
| GO:0070887 | BP | cellular response to chemical stimulus | 119 | 2919 | 8.10E-07 |
| GO:0009697 | BP | salicylic acid biosynthetic process | 30 | 388 | 8.50E-07 |
| GO:0009816 | BP | defense response to bacterium, incompatible interaction | 17 | 137 | 1.40E-06 |
| GO:0009723 | BP | response to ethylene | 43 | 714 | 1.40E-06 |
| GO:0009817 | BP | defense response to fungus, incompatible interaction | 15 | 106 | 1.60E-06 |
| GO:0006820 | BP | anion transport | 46 | 796 | 1.60E-06 |
| GO:0009404 | BP | toxin metabolic process | 26 | 319 | 2.50E-06 |
| GO:0044550 | BP | secondary metabolite biosynthetic process | 52 | 984 | 3.70E-06 |
| GO:0048583 | BP | regulation of response to stimulus | 89 | 2087 | 4.80E-06 |
| GO:0009812 | BP | flavonoid metabolic process | 37 | 595 | 5.30E-06 |
| GO:0009813 | BP | flavonoid biosynthetic process | 35 | 552 | 7.10E-06 |
| GO:0033993 | BP | response to lipid | 74 | 1650 | 7.20E-06 |
| GO:0010167 | BP | response to nitrate | 30 | 437 | 9.90E-06 |
| GO:0009651 | BP | response to salt stress | 61 | 1302 | 1.80E-05 |
| GO:0023014 | BP | signal transduction by protein phosphorylation | 31 | 481 | 2.40E-05 |
| GO:2000762 | BP | regulation of phenylpropanoid metabolic process | 26 | 365 | 3.00E-05 |
| GO:0042221 | BP | response to chemical | 201 | 5941 | 3.20E-05 |
| GO:0016310 | BP | phosphorylation | 91 | 2260 | 3.30E-05 |
| GO:0000165 | BP | MAPK cascade | 28 | 416 | 3.40E-05 |
| GO:0044036 | BP | cell wall macromolecule metabolic process | 32 | 517 | 3.60E-05 |
| GO:0009737 | BP | response to abscisic acid | 56 | 1188 | 3.90E-05 |
| GO:0009699 | BP | phenylpropanoid biosynthetic process | 29 | 446 | 4.20E-05 |
| GO:0032787 | BP | monocarboxylic acid metabolic process | 78 | 1866 | 4.40E-05 |
| GO:0009962 | BP | regulation of flavonoid biosynthetic process | 24 | 328 | 4.60E-05 |
| GO:0010106 | BP | cellular response to iron ion starvation | 21 | 261 | 4.80E-05 |
| GO:0071396 | BP | cellular response to lipid | 45 | 881 | 5.10E-05 |
| GO:0009407 | BP | toxin catabolic process | 22 | 285 | 5.20E-05 |
| GO:0090487 | BP | secondary metabolite catabolic process | 22 | 285 | 5.20E-05 |
| GO:1900376 | BP | regulation of secondary metabolite biosynthetic process | 24 | 335 | 6.40E-05 |
| GO:0010383 | BP | cell wall polysaccharide metabolic process | 28 | 435 | 7.20E-05 |
| GO:1900378 | BP | positive regulation of secondary metabolite biosynthetic process | 21 | 269 | 7.30E-05 |
| GO:0015711 | BP | organic anion transport | 25 | 362 | 7.30E-05 |
| GO:0009963 | BP | positive regulation of flavonoid biosynthetic process | 21 | 271 | 8.00E-05 |
| GO:0043455 | BP | regulation of secondary metabolic process | 26 | 389 | 8.20E-05 |
| GO:0006970 | BP | response to osmotic stress | 61 | 1379 | 8.80E-05 |
| GO:0097305 | BP | response to alcohol | 62 | 1420 | 0.00011 |
| GO:0007165 | BP | signal transduction | 149 | 4307 | 0.00013 |
| GO:0034976 | BP | response to endoplasmic reticulum stress | 34 | 612 | 0.00015 |
| GO:0046942 | BP | carboxylic acid transport | 24 | 356 | 0.00016 |
| GO:0006984 | BP | ER-nucleus signaling pathway | 27 | 431 | 0.00017 |
| GO:0030968 | BP | endoplasmic reticulum unfolded protein response | 26 | 407 | 0.00017 |
| GO:0034620 | BP | cellular response to unfolded protein | 26 | 411 | 0.0002 |
| GO:0048585 | BP | negative regulation of response to stimulus | 34 | 623 | 0.00021 |
| GO:0071705 | BP | nitrogen compound transport | 46 | 967 | 0.00021 |
| GO:0031640 | BP | killing of cells of other organism | 5 | 12 | 0.00023 |
| GO:0044364 | BP | disruption of cells of other organism | 5 | 12 | 0.00023 |
| GO:0072330 | BP | monocarboxylic acid biosynthetic process | 47 | 1002 | 0.00023 |
| GO:0035967 | BP | cellular response to topologically incorrect protein | 26 | 416 | 0.00024 |
| GO:0019752 | BP | carboxylic acid metabolic process | 97 | 2624 | 0.00034 |
| GO:0006950 | BP | response to stress | 214 | 6710 | 0.00036 |
| GO:0044700 | BP | single organism signaling | 150 | 4449 | 0.00036 |
| GO:0060548 | BP | negative regulation of cell death | 21 | 302 | 0.00037 |
| GO:0070592 | BP | cell wall polysaccharide biosynthetic process | 21 | 303 | 0.00038 |
| GO:0043069 | BP | negative regulation of programmed cell death | 20 | 282 | 0.00043 |
| GO:0071215 | BP | cellular response to abscisic acid stimulus | 32 | 593 | 0.00043 |
| GO:0009738 | BP | abscisic acid-activated signaling pathway | 30 | 542 | 0.00049 |
| GO:0006986 | BP | response to unfolded protein | 26 | 435 | 0.00049 |
| GO:0046394 | BP | carboxylic acid biosynthetic process | 54 | 1252 | 0.0005 |
| GO:0007154 | BP | cell communication | 165 | 5016 | 0.0005 |
| GO:0043436 | BP | oxoacid metabolic process | 102 | 2828 | 0.0005 |
| GO:0009743 | BP | response to carbohydrate | 51 | 1165 | 0.00056 |
| GO:0044038 | BP | cell wall macromolecule biosynthetic process | 21 | 315 | 0.00063 |
| GO:0070589 | BP | cellular component macromolecule biosynthetic process | 21 | 315 | 0.00063 |
| GO:0015809 | BP | arginine transport | 6 | 25 | 0.00078 |
| GO:0006082 | BP | organic acid metabolic process | 108 | 3071 | 0.00078 |
| GO:0034329 | BP | cell junction assembly | 7 | 37 | 0.0008 |
| GO:0031668 | BP | cellular response to extracellular stimulus | 38 | 790 | 0.00083 |
| GO:0016053 | BP | organic acid biosynthetic process | 61 | 1509 | 0.00093 |
| GO:0033554 | BP | cellular response to stress | 103 | 2918 | 0.00096 |
| GO:0042343 | BP | indole glucosinolate metabolic process | 7 | 39 | 0.0011 |
| GO:0071496 | BP | cellular response to external stimulus | 38 | 802 | 0.0011 |
| GO:0006612 | BP | protein targeting to membrane | 19 | 279 | 0.0011 |
| GO:0016143 | BP | S-glycoside metabolic process | 7 | 39 | 0.0011 |
| GO:0042430 | BP | indole-containing compound metabolic process | 15 | 190 | 0.0014 |
| GO:0023052 | BP | signaling | 157 | 4867 | 0.0015 |
| GO:0043090 | BP | amino acid import | 16 | 216 | 0.0017 |
| GO:0009835 | BP | fruit ripening | 8 | 57 | 0.0018 |
| GO:0010035 | BP | response to inorganic substance | 78 | 2120 | 0.0019 |
| GO:0097306 | BP | cellular response to alcohol | 34 | 705 | 0.0019 |
| GO:0045216 | BP | cell-cell junction organization | 7 | 43 | 0.002 |
| GO:0002237 | BP | response to molecule of bacterial origin | 15 | 197 | 0.002 |
| GO:0034333 | BP | adherens junction assembly | 6 | 30 | 0.0021 |
| GO:0007044 | BP | cell-substrate junction assembly | 6 | 30 | 0.0021 |
| GO:0048041 | BP | focal adhesion assembly | 6 | 30 | 0.0021 |
| GO:0007045 | BP | cell-substrate adherens junction assembly | 6 | 30 | 0.0021 |
| GO:0006865 | BP | amino acid transport | 17 | 246 | 0.0022 |
| GO:0002252 | BP | immune effector process | 27 | 510 | 0.0022 |
| GO:0009624 | BP | response to nematode | 19 | 297 | 0.0023 |
| GO:0015800 | BP | acidic amino acid transport | 6 | 31 | 0.0024 |
| GO:0044283 | BP | small molecule biosynthetic process | 80 | 2213 | 0.0025 |
| GO:0015804 | BP | neutral amino acid transport | 11 | 115 | 0.0025 |
| GO:0016137 | BP | glycoside metabolic process | 17 | 253 | 0.0029 |
| GO:0015849 | BP | organic acid transport | 18 | 279 | 0.0031 |
| GO:0044710 | BP | single-organism metabolic process | 266 | 8974 | 0.0031 |
| GO:0034330 | BP | cell junction organization | 7 | 47 | 0.0033 |
| GO:0090150 | BP | establishment of protein localization to membrane | 20 | 333 | 0.0034 |
| GO:0001906 | BP | cell killing | 5 | 21 | 0.0034 |
| GO:0071369 | BP | cellular response to ethylene stimulus | 20 | 335 | 0.0036 |
| GO:0051716 | BP | cellular response to stimulus | 188 | 6126 | 0.0038 |
| GO:0042445 | BP | hormone metabolic process | 31 | 651 | 0.0042 |
| GO:0071554 | BP | cell wall organization or biogenesis | 53 | 1348 | 0.0044 |
| GO:0015706 | BP | nitrate transport | 16 | 241 | 0.0051 |
| GO:0002831 | BP | regulation of response to biotic stimulus | 11 | 126 | 0.0052 |
| GO:0034332 | BP | adherens junction organization | 6 | 36 | 0.0053 |
| GO:0009991 | BP | response to extracellular stimulus | 38 | 878 | 0.0056 |
| GO:0009415 | BP | response to water | 35 | 786 | 0.0057 |
| GO:0072657 | BP | protein localization to membrane | 20 | 348 | 0.0057 |
| GO:0035304 | BP | regulation of protein dephosphorylation | 14 | 197 | 0.0062 |
| GO:0046283 | BP | anthocyanin-containing compound metabolic process | 16 | 246 | 0.0062 |
| GO:0042435 | BP | indole-containing compound biosynthetic process | 8 | 70 | 0.0065 |
| GO:0044711 | BP | single-organism biosynthetic process | 131 | 4128 | 0.0071 |
| GO:0045491 | BP | xylan metabolic process | 16 | 250 | 0.0073 |
| GO:0009759 | BP | indole glucosinolate biosynthetic process | 5 | 25 | 0.0076 |
| GO:0016144 | BP | S-glycoside biosynthetic process | 5 | 25 | 0.0076 |
| GO:0009267 | BP | cellular response to starvation | 30 | 647 | 0.0076 |
| GO:0015802 | BP | basic amino acid transport | 9 | 91 | 0.0076 |
| GO:0009808 | BP | lignin metabolic process | 12 | 156 | 0.0081 |
| GO:0035303 | BP | regulation of dephosphorylation | 14 | 204 | 0.0084 |
| GO:0035966 | BP | response to topologically incorrect protein | 30 | 652 | 0.0085 |
| GO:0009414 | BP | response to water deprivation | 33 | 744 | 0.0085 |
| GO:0048878 | BP | chemical homeostasis | 32 | 714 | 0.0086 |
| GO:0009628 | BP | response to abiotic stimulus | 124 | 3902 | 0.0086 |
| GO:0009625 | BP | response to insect | 10 | 115 | 0.0096 |
| GO:0042546 | BP | cell wall biogenesis | 32 | 721 | 0.01 |
| GO:0006826 | BP | iron ion transport | 10 | 116 | 0.01 |
| GO:1905039 | BP | carboxylic acid transmembrane transport | 8 | 76 | 0.01 |
| GO:1903825 | BP | organic acid transmembrane transport | 8 | 79 | 0.014 |
| GO:0009636 | BP | response to toxic substance | 12 | 168 | 0.015 |
| GO:0031669 | BP | cellular response to nutrient levels | 30 | 677 | 0.015 |
| GO:0016138 | BP | glycoside biosynthetic process | 12 | 168 | 0.015 |
| GO:0010410 | BP | hemicellulose metabolic process | 16 | 272 | 0.017 |
| GO:0010413 | BP | glucuronoxylan metabolic process | 14 | 220 | 0.017 |
| GO:0052482 | BP | defense response by cell wall thickening | 8 | 82 | 0.017 |
| GO:0052544 | BP | defense response by callose deposition in cell wall | 8 | 82 | 0.017 |
| GO:0009692 | BP | ethylene metabolic process | 12 | 172 | 0.017 |
| GO:0009693 | BP | ethylene biosynthetic process | 12 | 172 | 0.017 |
| GO:0007160 | BP | cell-matrix adhesion | 6 | 46 | 0.017 |
| GO:0043449 | BP | cellular alkene metabolic process | 12 | 172 | 0.017 |
| GO:0006499 | BP | N-terminal protein myristoylation | 15 | 248 | 0.017 |
| GO:0006498 | BP | N-terminal protein lipidation | 15 | 248 | 0.017 |
| GO:0009809 | BP | lignin biosynthetic process | 10 | 125 | 0.017 |
| GO:1900674 | BP | olefin biosynthetic process | 12 | 172 | 0.017 |
| GO:1900673 | BP | olefin metabolic process | 12 | 172 | 0.017 |
| GO:0043450 | BP | alkene biosynthetic process | 12 | 172 | 0.017 |
| GO:0042594 | BP | response to starvation | 30 | 688 | 0.018 |
| GO:0071462 | BP | cellular response to water stimulus | 12 | 173 | 0.018 |
| GO:0018377 | BP | protein myristoylation | 15 | 249 | 0.018 |
| GO:0042631 | BP | cellular response to water deprivation | 12 | 173 | 0.018 |
| GO:0009718 | BP | anthocyanin-containing compound biosynthetic process | 13 | 201 | 0.021 |
| GO:0009873 | BP | ethylene-activated signaling pathway | 16 | 283 | 0.023 |
| GO:0045492 | BP | xylan biosynthetic process | 14 | 230 | 0.023 |
| GO:0010817 | BP | regulation of hormone levels | 38 | 961 | 0.023 |
| GO:0046777 | BP | protein autophosphorylation | 18 | 340 | 0.024 |
| GO:0009805 | BP | coumarin biosynthetic process | 11 | 155 | 0.024 |
| GO:0009804 | BP | coumarin metabolic process | 11 | 155 | 0.024 |
| GO:0010043 | BP | response to zinc ion | 12 | 180 | 0.024 |
| GO:0071472 | BP | cellular response to salt stress | 8 | 89 | 0.026 |
| GO:0005976 | BP | polysaccharide metabolic process | 39 | 1002 | 0.026 |
| GO:0010252 | BP | auxin homeostasis | 6 | 51 | 0.028 |
| GO:0009611 | BP | response to wounding | 25 | 559 | 0.031 |
| GO:0052543 | BP | callose deposition in cell wall | 8 | 92 | 0.032 |
| GO:0008202 | BP | steroid metabolic process | 21 | 439 | 0.033 |
| GO:0050896 | BP | response to stimulus | 329 | 11973 | 0.036 |
| GO:0044765 | BP | single-organism transport | 76 | 2326 | 0.037 |
| GO:0031589 | BP | cell-substrate adhesion | 6 | 54 | 0.037 |
| GO:0045488 | BP | pectin metabolic process | 12 | 193 | 0.042 |
| GO:0052546 | BP | cell wall pectin metabolic process | 11 | 168 | 0.043 |
| GO:0010393 | BP | galacturonan metabolic process | 12 | 194 | 0.044 |
| GO:0000041 | BP | transition metal ion transport | 14 | 248 | 0.044 |
| GO:0031365 | BP | N-terminal protein amino acid modification | 15 | 277 | 0.046 |
| GO:0052386 | BP | cell wall thickening | 8 | 99 | 0.049 |
| GO:0004674 | MF | protein serine/threonine kinase activity | 57 | 876 | 3.10E-09 |
| GO:0004672 | MF | protein kinase activity | 74 | 1316 | 3.10E-09 |
| GO:0030247 | MF | polysaccharide binding | 17 | 99 | 1.70E-08 |
| GO:0001871 | MF | pattern binding | 17 | 99 | 1.70E-08 |
| GO:0030246 | MF | carbohydrate binding | 26 | 251 | 2.70E-08 |
| GO:0016773 | MF | phosphotransferase activity, alcohol group as acceptor | 78 | 1538 | 3.40E-08 |
| GO:0030554 | MF | adenyl nucleotide binding | 93 | 2046 | 1.90E-07 |
| GO:0032559 | MF | adenyl ribonucleotide binding | 90 | 2012 | 5.70E-07 |
| GO:0097367 | MF | carbohydrate derivative binding | 101 | 2361 | 6.90E-07 |
| GO:0016301 | MF | kinase activity | 84 | 1893 | 1.70E-06 |
| GO:0016772 | MF | transferase activity, transferring phosphorus-containing groups | 107 | 2631 | 2.60E-06 |
| GO:0005524 | MF | ATP binding | 83 | 1905 | 3.60E-06 |
| GO:0017076 | MF | purine nucleotide binding | 95 | 2290 | 4.30E-06 |
| GO:0032550 | MF | purine ribonucleoside binding | 92 | 2242 | 8.70E-06 |
| GO:0001883 | MF | purine nucleoside binding | 92 | 2242 | 8.70E-06 |
| GO:0032555 | MF | purine ribonucleotide binding | 92 | 2250 | 8.90E-06 |
| GO:0032549 | MF | ribonucleoside binding | 92 | 2249 | 8.90E-06 |
| GO:0001882 | MF | nucleoside binding | 92 | 2254 | 9.00E-06 |
| GO:0032553 | MF | ribonucleotide binding | 93 | 2293 | 9.70E-06 |
| GO:0050266 | MF | rosmarinate synthase activity | 8 | 32 | 1.50E-05 |
| GO:0020037 | MF | heme binding | 21 | 267 | 3.40E-05 |
| GO:0016705 | MF | oxidoreductase activity, acting on paired donors, with incorporation or reduction of molecular oxygen | 24 | 340 | 4.00E-05 |
| GO:0035639 | MF | purine ribonucleoside triphosphate binding | 85 | 2143 | 5.10E-05 |
| GO:0016709 | MF | oxidoreductase activity, acting on paired donors, with incorporation or reduction of molecular oxygen, NAD(P)H as one donor, and incorporation of one atom of oxygen | 17 | 193 | 6.60E-05 |
| GO:0008061 | MF | chitin binding | 7 | 29 | 8.40E-05 |
| GO:0046906 | MF | tetrapyrrole binding | 21 | 305 | 0.00022 |
| GO:0047893 | MF | flavonol 3-O-glucosyltransferase activity | 9 | 65 | 0.0004 |
| GO:0050734 | MF | hydroxycinnamoyltransferase activity | 8 | 53 | 0.0006 |
| GO:0080043 | MF | quercetin 3-O-glucosyltransferase activity | 10 | 87 | 0.00067 |
| GO:0008353 | MF | RNA polymerase II carboxy-terminal domain kinase activity | 6 | 28 | 0.00077 |
| GO:0004497 | MF | monooxygenase activity | 17 | 241 | 0.00093 |
| GO:0047172 | MF | shikimate O-hydroxycinnamoyltransferase activity | 7 | 45 | 0.0014 |
| GO:0050737 | MF | O-hydroxycinnamoyltransferase activity | 7 | 50 | 0.0027 |
| GO:0035251 | MF | UDP-glucosyltransferase activity | 14 | 199 | 0.004 |
| GO:0050366 | MF | tyramine N-feruloyltransferase activity | 6 | 41 | 0.006 |
| GO:0004675 | MF | transmembrane receptor protein serine/threonine kinase activity | 6 | 41 | 0.006 |
| GO:0005506 | MF | iron ion binding | 16 | 264 | 0.0072 |
| GO:0046527 | MF | glucosyltransferase activity | 14 | 220 | 0.0098 |
| GO:0001071 | MF | nucleic acid binding transcription factor activity | 32 | 753 | 0.01 |
| GO:0050505 | MF | hydroquinone glucosyltransferase activity | 5 | 31 | 0.01 |
| GO:0047205 | MF | quinate O-hydroxycinnamoyltransferase activity | 6 | 46 | 0.01 |
| GO:0080044 | MF | quercetin 7-O-glucosyltransferase activity | 8 | 84 | 0.01 |
| GO:0003700 | MF | transcription factor activity, sequence-specific DNA binding | 32 | 753 | 0.01 |
| GO:0047218 | MF | hydroxycinnamate 4-beta-glucosyltransferase activity | 5 | 32 | 0.012 |
| GO:0043565 | MF | sequence-specific DNA binding | 22 | 456 | 0.013 |
| GO:0050403 | MF | trans-zeatin O-beta-D-glucosyltransferase activity | 6 | 50 | 0.014 |
| GO:0015179 | MF | L-amino acid transmembrane transporter activity | 7 | 69 | 0.014 |
| GO:1901265 | MF | nucleoside phosphate binding | 108 | 3479 | 0.016 |
| GO:0000166 | MF | nucleotide binding | 108 | 3479 | 0.016 |
| GO:0005275 | MF | amine transmembrane transporter activity | 7 | 75 | 0.022 |
| GO:0036094 | MF | small molecule binding | 108 | 3579 | 0.034 |
| GO:0015171 | MF | amino acid transmembrane transporter activity | 7 | 85 | 0.043 |
| GO:0050502 | MF | cis-zeatin O-beta-D-glucosyltransferase activity | 5 | 46 | 0.05 |
| GO:0004364 | MF | glutathione transferase activity | 5 | 46 | 0.05 |
| GO:0042729 | CP | DASH complex | 14 | 57 | 1.90E-08 |
| GO:0000942 | CP | condensed nuclear chromosome outer kinetochore | 14 | 59 | 1.90E-08 |
| GO:0000940 | CP | condensed chromosome outer kinetochore | 14 | 63 | 3.20E-08 |
| GO:0000778 | CP | condensed nuclear chromosome kinetochore | 14 | 74 | 2.30E-07 |
| GO:0012505 | CP | endomembrane system | 240 | 6907 | 2.00E-06 |
| GO:0000780 | CP | condensed nuclear chromosome, centromeric region | 14 | 93 | 3.40E-06 |
| GO:0000777 | CP | condensed chromosome kinetochore | 14 | 96 | 4.40E-06 |
| GO:0000779 | CP | condensed chromosome, centromeric region | 14 | 112 | 2.70E-05 |
| GO:0005783 | CP | endoplasmic reticulum | 111 | 3060 | 0.00036 |
| GO:0000776 | CP | kinetochore | 14 | 146 | 0.00054 |
| GO:0000794 | CP | condensed nuclear chromosome | 14 | 179 | 0.0048 |
| GO:0005576 | CP | extracellular region | 58 | 1515 | 0.0085 |
| GO:0016021 | CP | integral component of membrane | 193 | 6392 | 0.011 |
| GO:0031224 | CP | intrinsic component of membrane | 195 | 6532 | 0.016 |
| GO:0030312 | CP | external encapsulating structure | 48 | 1242 | 0.019 |
| GO:0044425 | CP | membrane part | 215 | 7355 | 0.022 |
| GO:0005618 | CP | cell wall | 44 | 1126 | 0.022 |
| GO:0000793 | CP | condensed chromosome | 15 | 243 | 0.022 |
| GO:0009506 | CP | plasmodesma | 52 | 1418 | 0.027 |
| GO:0055044 | CP | symplast | 52 | 1418 | 0.027 |
| GO:0005911 | CP | cell-cell junction | 53 | 1484 | 0.041 |

BP: Biological Process; MF: Molecular Function; CP: Cellular Process

# Supplementary Table 5. The GO terms significantly enriched during incompatible interaction at 72 hai in sorghum.

| GO term | Ontology | Description | Number in input list | Number in BG/Ref | FDR |
| --- | --- | --- | --- | --- | --- |
| GO:0006468 | BP | protein phosphorylation | 89 | 743 | 3.70E-06 |
| GO:0016310 | BP | phosphorylation | 94 | 814 | 4.40E-06 |
| GO:0009607 | BP | response to biotic stimulus | 7 | 12 | 0.00053 |
| GO:0055114 | BP | oxidation-reduction process | 76 | 723 | 0.0013 |
| GO:0071554 | BP | cell wall organization or biogenesis | 9 | 29 | 0.0041 |
| GO:0006952 | BP | defense response | 8 | 22 | 0.0041 |
| GO:0006796 | BP | phosphate-containing compound metabolic process | 98 | 1044 | 0.0041 |
| GO:0006793 | BP | phosphorus metabolic process | 98 | 1046 | 0.0041 |
| GO:0046348 | BP | amino sugar catabolic process | 5 | 11 | 0.011 |
| GO:0044703 | BP | multi-organism reproductive process | 8 | 30 | 0.011 |
| GO:0044706 | BP | multi-multicellular organism process | 8 | 30 | 0.011 |
| GO:0006040 | BP | amino sugar metabolic process | 5 | 11 | 0.011 |
| GO:1901072 | BP | glucosamine-containing compound catabolic process | 5 | 11 | 0.011 |
| GO:1901071 | BP | glucosamine-containing compound metabolic process | 5 | 11 | 0.011 |
| GO:0009067 | BP | aspartate family amino acid biosynthetic process | 5 | 11 | 0.011 |
| GO:0009875 | BP | pollen-pistil interaction | 8 | 30 | 0.011 |
| GO:0043412 | BP | macromolecule modification | 96 | 1070 | 0.011 |
| GO:0036211 | BP | protein modification process | 94 | 1031 | 0.011 |
| GO:0006026 | BP | aminoglycan catabolic process | 5 | 11 | 0.011 |
| GO:0006030 | BP | chitin metabolic process | 5 | 11 | 0.011 |
| GO:0006032 | BP | chitin catabolic process | 5 | 11 | 0.011 |
| GO:0008037 | BP | cell recognition | 8 | 30 | 0.011 |
| GO:0048544 | BP | recognition of pollen | 8 | 30 | 0.011 |
| GO:0051704 | BP | multi-organism process | 9 | 34 | 0.011 |
| GO:0006464 | BP | cellular protein modification process | 94 | 1031 | 0.011 |
| GO:0009856 | BP | pollination | 8 | 30 | 0.011 |
| GO:0044710 | BP | single-organism metabolic process | 119 | 1401 | 0.011 |
| GO:0016998 | BP | cell wall macromolecule catabolic process | 5 | 12 | 0.013 |
| GO:0009066 | BP | aspartate family amino acid metabolic process | 5 | 12 | 0.013 |
| GO:0006022 | BP | aminoglycan metabolic process | 5 | 12 | 0.013 |
| GO:0000003 | BP | reproduction | 8 | 33 | 0.017 |
| GO:0044702 | BP | single organism reproductive process | 8 | 33 | 0.017 |
| GO:0022414 | BP | reproductive process | 8 | 33 | 0.017 |
| GO:0044036 | BP | cell wall macromolecule metabolic process | 5 | 13 | 0.018 |
| GO:0044699 | BP | single-organism process | 162 | 2074 | 0.041 |
| GO:0004672 | MF | protein kinase activity | 91 | 739 | 4.00E-07 |
| GO:0016301 | MF | kinase activity | 97 | 855 | 3.70E-06 |
| GO:0016773 | MF | phosphotransferase activity, alcohol group as acceptor | 93 | 847 | 1.90E-05 |
| GO:0030246 | MF | carbohydrate binding | 21 | 94 | 2.40E-05 |
| GO:0001871 | MF | pattern binding | 13 | 40 | 3.30E-05 |
| GO:0030247 | MF | polysaccharide binding | 13 | 40 | 3.30E-05 |
| GO:0016772 | MF | transferase activity, transferring phosphorus-containing groups | 102 | 1010 | 0.0001 |
| GO:0016491 | MF | oxidoreductase activity | 85 | 828 | 0.00029 |
| GO:0003824 | MF | catalytic activity | 349 | 4420 | 0.0012 |
| GO:0008171 | MF | O-methyltransferase activity | 8 | 23 | 0.002 |
| GO:0020037 | MF | heme binding | 28 | 206 | 0.0029 |
| GO:0046906 | MF | tetrapyrrole binding | 28 | 211 | 0.0041 |
| GO:0016740 | MF | transferase activity | 149 | 1802 | 0.01 |
| GO:0004568 | MF | chitinase activity | 5 | 11 | 0.01 |
| GO:0032559 | MF | adenyl ribonucleotide binding | 98 | 1142 | 0.022 |
| GO:0016705 | MF | oxidoreductase activity, acting on paired donors, with incorporation or reduction of molecular oxygen | 22 | 170 | 0.022 |
| GO:0030554 | MF | adenyl nucleotide binding | 98 | 1146 | 0.023 |
| GO:0005506 | MF | iron ion binding | 22 | 177 | 0.034 |

BP: Biological Process; MF: Molecular Function

# Supplementary Table 6. Significantly enriched GO terms common to maize and sorghum during the incompatible interaction at 72 hai.

| **GO term** | **Ontology** | **Description** |
| --- | --- | --- |
| GO:0006468 | BP | protein phosphorylation |
| GO:0006952 | BP | defense response |
| GO:0009607 | BP | response to biotic stimulus |
| GO:0016310 | BP | phosphorylation |
| GO:0044036 | BP | cell wall macromolecule metabolic process |
| GO:0044710 | BP | single-organism metabolic process |
| GO:0051704 | BP | multi-organism process |
| GO:0071554 | BP | cell wall organization or biogenesis |
| GO:0001871 | MF | pattern binding |
| GO:0004672 | MF | protein kinase activity |
| GO:0005506 | MF | iron ion binding |
| GO:0016301 | MF | kinase activity |
| GO:0016705 | MF | oxidoreductase activity, acting on paired donors, with incorporation or reduction of molecular oxygen |
| GO:0016772 | MF | transferase activity, transferring phosphorus-containing groups |
| GO:0016773 | MF | phosphotransferase activity, alcohol group as acceptor |
| GO:0020037 | MF | heme binding |
| GO:0030246 | MF | carbohydrate binding |
| GO:0030247 | MF | polysaccharide binding |
| GO:0030554 | MF | adenyl nucleotide binding |
| GO:0032559 | MF | adenyl ribonucleotide binding |
| GO:0046906 | MF | tetrapyrrole binding |

BP: Biological Process; MF: Molecular Function

# Supplementary Table 7. Conserved orthologs differentially regulated in both maize and sorghum (shared DEOs).

| **Category based on annotation** | **Maize ortholog** | | **Sorghum ortholog ^a^** | **Interaction^b^** | **Maize annotation^c^** | **Sorghum annotation^d^** |
| --- | --- | --- | --- | --- | --- | --- |
|  | **Gramene Id** | **NCBI Id** |  |  |  |  |
| Transcription factor | GRMZM2G054125 | LOC103652912 | Sobic.005G117400 | Comp 24 hai | probable WRKY transcription factor 24 | NA |
| Transcription factor | GRMZM2G057116 | LOC100193498 | Sobic.009G068900 | Comp 24 hai | putative WRKY transcription factor 50 | similar to WRKY transcription factor 67 |
| Transcription factor | GRMZM5G863420 | LOC103630663 | Sobic.009G212800 | Comp 24 hai | probable WRKY transcription factor 50 | similar to WRKY transcription factor 7 |
| Transcription factor | GRMZM2G175480 | LOC103635561 | Sobic.009G073500 | Comp 24 hai; Incomp 24 hai | uncharacterized LOC103635561, transcript variant X1 | Predicted protein |
| Transcription factor | GRMZM2G069325 | LOC100272412 | Sobic.009G224500 | Incomp 72 hai | putative MYB DNA-binding domain superfamily protein | similar to Putative uncharacterized protein |
| Transcription factor | GRMZM2G169966 | LOC100281430 | Sobic.009G171600 | Incomp 72 hai | WRKY70 - superfamily of TFs having WRKY and zinc finger domains | similar to WRKY transcription factor 70 |
| Protease Inhibitor | GRMZM2G116520 | LOC103641458 | Sobic.005G215900 | Comp 72 hai; Incomp 72 hai | Bowman-Birk type trypsin inhibitor | similar to Putative Bowman-Birk serine protease inhibitor |
| Pathogenesis-related protein | GRMZM2G402631 | PR-5 | Sobic.008G182900 | Comp 24 hai | pathogenesis related protein-5 | similar to Pathogenesis related protein-5 |
| Pathogenesis-related protein | GRMZM2G117942 | LOC103652814 | Sobic.005G169200 | Comp 24 hai; Incomp 24 hai | barwin | similar to Pathogenesis-related protein 4 |
| Pathogenesis-related protein | GRMZM2G117971 | pco080661a | Sobic.005G169200 | Comp 24 hai; Incomp 24 hai | Hevein-like preproprotein | similar to Pathogenesis-related protein 4 |
| Pathogenesis-related protein | AC205274.3_FG001 | TIDP2793 | Sobic.010G020200 | Comp 72 hai; Incomp 72 hai | pathogenesis-related protein PRMS | similar to Pathogenesis-related protein PRMS precursor |
| Pathogenesis-related protein | GRMZM2G039639 | pco103560(319) | Sobic.001G145700 | Incomp 72 hai | Protein P21 | similar to Pathogenesis-related thaumatin-like protein |
| Pathogenesis-related protein | GRMZM2G099092 | LOC103629617 | Sobic.010G240400 | Incomp 72 hai | EG45-like domain containing protein | similar to Putative uncharacterized protein |
| Pathogenesis-related protein | GRMZM2G112524 | pco091453a | Sobic.001G400800 | Incomp 72 hai | uncharacterized LOC100284195 | NA |
| Pathogenesis-related protein | GRMZM2G112524 | pco091453a | Sobic.001G400700 | Incomp 72 hai | uncharacterized LOC100284195 | NA |
| Pathogenesis-related protein | GRMZM2G112524 | pco091453a | Sobic.001G401300 | Incomp 72 hai | uncharacterized LOC100284195 | similar to Pathogenesis-related protein 10a |
| Pathogenesis-related protein | GRMZM2G112524 | pco091453a | Sobic.001G401200 | Incomp 72 hai | uncharacterized LOC100284195 | similar to Pathogenesis-related protein 10a |
| Pathogenesis-related protein | GRMZM2G112524 | pco091453a | Sobic.001G400900 | Incomp 72 hai | uncharacterized LOC100284195 | similar to Pathogenesis-related protein 10a |
| Pathogenesis-related protein | GRMZM2G112538 | IDP2565 | Sobic.001G400800 | Incomp 72 hai | uncharacterized LOC100192117 | NA |
| Pathogenesis-related protein | GRMZM2G112538 | IDP2565 | Sobic.001G400700 | Incomp 72 hai | uncharacterized LOC100192117 | NA |
| Pathogenesis-related protein | GRMZM2G112538 | IDP2565 | Sobic.001G401300 | Incomp 72 hai | uncharacterized LOC100192117 | similar to Pathogenesis-related protein 10a |
| Pathogenesis-related protein | GRMZM2G112538 | IDP2565 | Sobic.001G400900 | Incomp 72 hai | uncharacterized LOC100192117 | similar to Pathogenesis-related protein 10a |
| Pathogenesis-related protein | GRMZM2G136372 | LOC103640644 | Sobic.008G182600 | Incomp 72 hai | thaumatin-like protein | similar to Pathogenesis related protein-5 |
| Pathogenesis-related protein | GRMZM2G374971 | sip1 | Sobic.002G351400 | Incomp 72 hai | stress-induced protein 1 | similar to Zeamatin precursor |
| Pathogenesis-related protein | GRMZM2G465226 | prp4 | Sobic.002G023300 | Incomp 72 hai | pathogenesis related protein 4 | similar to Pathogenesis related protein-1 |
| Hormone related | GRMZM2G052100 | LOC100127507 | Sobic.006G078400 | Incomp 72 hai | Asr protein | similar to Putative uncharacterized protein |
| Disease-resistance protein | GRMZM2G059012 | LOC103641905 | Sobic.007G214300 | Incomp 72 hai | wall-associated receptor kinase 5, transcript variant X2 | NA |
| Disease-resistance protein | GRMZM2G076943 | LOC100279957 | Sobic.009G082200 | Incomp 72 hai | putative DUF26-domain receptor-like protein kinase family protein | similar to Putative serine/threonine kinase protein |
| Disease-resistance protein | GRMZM2G104655 | LOC100191493 | Sobic.008G013800 | Incomp 72 hai | putative lectin-domain receptor-like protein kinase family protein | weakly similar to Os09g0334800 protein |
| Disease-resistance protein | GRMZM2G137861 | LOC103641603 | Sobic.007G214300 | Incomp 72 hai | wall-associated receptor kinase 2, transcript variant X2 | NA |
| Disease-resistance protein | GRMZM2G174128 | LOC103627026 | Sobic.004G103400 | Incomp 72 hai | receptor like protein 42, transcript variant X1 | NA |
| Disease-resistance protein | GRMZM2G176206 | LOC103627070 | Sobic.004G099700 | Incomp 72 hai | receptor-like protein kinase 5 | similar to Putative uncharacterized protein |
| Disease-resistance protein | GRMZM2G177883 | LOC100382682 | Sobic.003G291600 | Incomp 72 hai | Protein kinase family protein with leucine-rich repeat domain | NA |
| Disease-resistance protein | GRMZM2G340654 | LOC103641520 | Sobic.001G159200 | Incomp 72 hai | G-type lectin S-receptor-like serine/threonine-protein kinase SD2-5 | NA |
| Disease-resistance protein | GRMZM2G426917 | LOC103649884 | Sobic.003G097100 | Incomp 72 hai | rust resistance kinase Lr10, transcript variant X2 | similar to Putative rust resistance kinase Lr10 |
| Disease-resistance protein | GRMZM2G436448 | LOC103641459 | Sobic.003G192600 | Incomp 72 hai | rust resistance kinase Lr10 | similar to Receptor-like kinase |
| Disease-resistance protein | GRMZM5G832149 | LOC100281606 | Sobic.003G081100 | Incomp 72 hai | WAK2 - OsWAK receptor-like cytoplasmic kinase (OsWAK-RLCK) | similar to Os01g0136800 protein |
| Defense-related | GRMZM2G156632 | wip1 | Sobic.003G085300 | Comp 24 hai | wound induced protein 1 | NA |
| defense-related | GRMZM2G119150 | LOC103632470 | Sobic.001G471100 | Comp 24 hai; Incomp 72 hai | aminotransferase ALD1 homolog | similar to Putative uncharacterized protein |
| Defense-related | GRMZM2G173192 | LOC100193279 | Sobic.004G004500 | Comp 24 hai; Incomp 24 hai; Incomp 72 hai | Lactate/malate dehydrogenase family protein | similar to L-lactate dehydrogenase |
| Defense-related | GRMZM2G093092 | LOC103638999 | Sobic.001G354400 | Comp 72 hai; Incomp 72 hai | 5-pentadecatrienyl resorcinol O-methyltransferase | NA |
| Defense-related | GRMZM2G339091 | LOC103630156 | Sobic.002G024400 | Incomp 72 hai | serine carboxypeptidase 2, transcript variant X1 | similar to Putative serine carboxypeptidase II |
| Cytochrome P450 | GRMZM2G399530 | LOC103641574 | Sobic.010G230900 | Comp 72 hai; Incomp 72 hai | indolin-2-one monooxygenase-like | similar to Putative uncharacterized protein |
| Cytochrome P450 | GRMZM2G102662 | LOC103640866 | Sobic.009G064400 | Incomp 72 hai | cytochrome P450 71A1 | similar to Cytochrome P450 family protein, expressed |
| Cytochrome P450 | GRMZM2G122654 | LOC100274356 | Sobic.001G235500 | Incomp 72 hai | putative cytochrome P450 superfamily protein | similar to Cytochrome P450 family protein, expressed |
| Chitinase | GRMZM2G358153 | LOC100192522 | Sobic.005G084300 | Comp 24 hai | chitinase 1 | similar to Chitinase 1, putative, expressed |
| Chitinase | GRMZM2G099454 | LOC100191592 | Sobic.001G516000 | Incomp 72 hai | basic endochitinase | similar to Basic endochitinase, putative, expressed |
| Transporter | GRMZM2G084779 | kup1 | Sobic.003G413700 | Incomp 72 hai | potasium ion uptake permease 1 | similar to Putative potassium transporter |
| miscellaneous | GRMZM5G863076 | LOC100285307 | Sobic.002G113100 | Comp 24 hai; Incomp 72 hai | plant integral membrane protein TIGR01569 containing protein | similar to Integral membrane protein-like |
| miscellaneous | GRMZM2G116629 | si707033a12 | Sobic.007G001100 | Comp 72 hai; Incomp 72 hai | lipopolysaccharide-modifying protein | similar to Putative uncharacterized protein |
| miscellaneous | GRMZM2G018254 | LOC100279519 | Sobic.005G230000 | Incomp 72 hai | uncharacterized LOC100279519 | weakly similar to GRAS family transcription factor, putative |
| miscellaneous | GRMZM2G026143 | LOC103638673 | Sobic.010G178000 | Incomp 72 hai | uncharacterized LOC103638673 | NA |
| miscellaneous | GRMZM2G032047 | AY110632 | Sobic.001G403600 | Incomp 72 hai | uncharacterized LOC100283541 | similar to Expressed protein |
| miscellaneous | GRMZM2G074743 | aox3 | Sobic.006G203000 | Incomp 72 hai | alternative oxidase AOX3 precursor | similar to Alternative oxidase |
| miscellaneous | GRMZM2G082257 | LOC100284635 | Sobic.007G136700 | Incomp 72 hai | YGL010w-like protein, transcript variant X1 | similar to Putative uncharacterized protein P0431A03.22 |
| miscellaneous | GRMZM2G089506 | LOC100382179 | Sobic.010G128000 | Incomp 72 hai | Aspartyl protease AED1 | similar to Putative uncharacterized protein |
| miscellaneous | GRMZM2G092146 | LOC100275352 | Sobic.010G201200 | Incomp 72 hai | uncharacterized LOC100275352 | NA |
| miscellaneous | GRMZM2G115504 | LOC100284064 | Sobic.004G165200 | Incomp 72 hai | catalytic/ hydrolase, transcript variant X1 | similar to Hydrolase, alpha/beta fold protein-like |
| miscellaneous | GRMZM2G124815 | LOC103629360 | Sobic.010G001300 | Incomp 72 hai | HXXXD-type acyl-transferase family protein | similar to Putative uncharacterized protein |
| miscellaneous | GRMZM2G136508 | LOC100281764 | Sobic.006G084600 | Incomp 72 hai | amino acid permease | similar to OSIGBa0102D10.6 protein |
| miscellaneous | GRMZM2G151230 | CNR02 | Sobic.004G192100 | Incomp 72 hai | cell number regulator 2 | similar to Putative ORFX |
| miscellaneous | GRMZM2G153208 | IDP820 | Sobic.001G209300 | Incomp 72 hai | Secretory protein | similar to Putative uncharacterized protein precursor |
| miscellaneous | GRMZM2G174449 | LOC100273307 | Sobic.007G115500 | Incomp 72 hai | uncharacterized LOC100273307 | weakly similar to Putative uncharacterized protein |
| miscellaneous | GRMZM2G374827 | LOC103628955 | Sobic.007G011700 | Incomp 72 hai | non-lysosomal glucosylceramidase | similar to Putative Bile acid beta-glucosidase |
| miscellaneous | GRMZM2G407044 | LOC100284799 | Sobic.004G210000 | Incomp 72 hai | acetolactate synthase/ amino acid binding protein | similar to Acetolactate synthase, small subunit, putative |
| miscellaneous | GRMZM2G423472 | LOC103641011 | Sobic.008G191600 | Incomp 72 hai | vesicle-associated membrane protein 721, transcript variant X2 | similar to CDS_VAMP |
| miscellaneous | GRMZM2G449094 | LOC100384448 | Sobic.006G267000 | Incomp 72 hai | uncharacterized LOC100384448 | similar to H0402C08.7 protein |

^a^The sorghum orthologs id is based on Phytozome v12 id.

^b^Interaction at which the shared DEOs were observed: Comp 24 indicates compatible interaction at 24 hours after inoculation (hai), comp 72 indicates compatible interaction at 72 hai, incompatible interaction at 24 hai, and incompatible interaction at 72 hai

^c^Maize annotations are obtained from NCBI database

^d^Sorghum annotations are obtained from phytozome database

# Supplementary Table 8. The common genes that are differentially expressed in all interaction-time combinations with their respective fold change compared to mock in maize.

| **Maize Genes ^a^** | **Gramene Id** | | **Fold change compared to mock** | | | | **Product^b^** |
| --- | --- | --- | --- | --- | --- | --- | --- |
|  |  |  | **Compatible at 24 hai** | **Incompatible at 24 hai** | **Compatible at 72 hai** | **Incompatible at 72 hai** |  |
| LOC103633263 | | GRMZM2G431039 | 132 | 123 | 285 | 2400 | glucan endo-1,3-beta-glucosidase 13, transcript variant X1 |
| geb1 | GRMZM2G065585 | | 694 | 435 | 402 | 1902 | glucan endo-1,3-beta-glucosidase homolog 1 |
| pco080661a | GRMZM2G117971 | | 839 | 395 | 294 | 1296 | Hevein-like preproprotein |
| LOC103652814 | GRMZM2G117942 | | 496 | 244 | 99 | 450 | barwin |
| PR-5 | GRMZM2G402631 | | 98 | 65 | 101 | 430 | pathogenesis related protein-5 |
| LOC103648077 | GRMZM2G372058 | | 72 | 42 | 36 | 87 | LRR receptor-like serine/threonine-protein kinase FLS2 |
| LOC103641459 | GRMZM2G436448 | | 34 | 21 | 72 | 154 | rust resistance kinase Lr10 |
| LOC103631918 | GRMZM2G456997 | | 28 | 40 | 15 | 27 | pathogenesis-related protein PRB1-3 |
| LOC103646336 | GRMZM2G119975 | | 17 | 16 | 20 | 79 | heavy metal-associated isoprenylated plant protein 4 |
| LOC100193498 | GRMZM2G057116 | | 17 | 17 | 9 | 28 | putative WRKY transcription factor 50 |
| LOC100281558 | GRMZM2G012724 | | 10 | 8 | 5 | 11 | WRKY53 - superfamily of TFs having WRKY and zinc finger domains, transcript variant X1 |
| LOC100281947 | GRMZM2G137802 | | 22 | 14 | 11 | 33 | WRKY7 - superfamily of TFs having WRKY and zinc finger domains, transcript variant X1 |
| LOC103635353 | GRMZM2G063880 | | 9 | 8 | 6 | 14 | probable WRKY transcription factor 41 |
| bHLH94 | GRMZM5G849600 | | 17 | 17 | 29 | 76 | putative HLH DNA-binding domain superfamily protein |
| LOC100193279 | GRMZM2G173192 | | 51 | 36 | 26 | 68 | Lactate/malate dehydrogenase family protein |
| LOC103627130 | GRMZM2G325014 | | 7 | 5 | 8 | 15 | UDP-glycosyltransferase 73D1-like |
| LOC100382268 | GRMZM2G176472 | | 17 | 12 | 18 | 59 | Calmodulin-binding protein 60 G, transcript variant X1 |
| LOC100382651 | no_grmzm_overlap | | 19 | 11 | 15 | 24 | cytochrome P450 family 72 subfamily A polypeptide 8 |
| LOC100281118 | GRMZM2G459663 | | 50 | 28 | 13 | 42 | EF hand family protein |
| LOC103627498 | GRMZM2G466044 | | 9 | 8 | 9 | 21 | ethylene-responsive transcription factor 2 |
| LOC100281643 | GRMZM2G450273 | | 7 | 8 | 7 | 22 | flowering promoting factor-like 1 |
| LOC100383595 | GRMZM5G893912 | | 46 | 28 | 17 | 25 | AAA-ATPase ASD mitochondrial |
| LOC100285307 | GRMZM5G863076 | | 16 | 11 | 31 | 64 | plant integral membrane protein TIGR01569 containing protein |
| LOC100281943 | GRMZM2G053833 | | 40 | 19 | 12 | 58 | probable calcium-binding protein CML45 |
| LOC103626249 | AC197764.4_FG003 | | 29 | 21 | 31 | 78 | protein TIFY 10b |
| LOC100273307 | GRMZM2G174449 | | 15 | 9 | 8 | 9 | uncharacterized LOC100273307 |
| LOC100274400 | GRMZM2G145461 | | 185 | 96 | 130 | 664 | uncharacterized LOC100274400 |
| si829009d02d | GRMZM2G131099 | | 40 | 22 | 35 | 79 | uncharacterized LOC100275282 |
| LOC100279471 | GRMZM2G374309 | | 12 | 10 | 54 | 115 | uncharacterized LOC100279471 |
| TIDP3648 | GRMZM2G130728 | | 32 | 18 | 83 | 188 | uncharacterized LOC100285022 |
| LOC100501753 | GRMZM2G147752 | | 17 | 13 | 19 | 32 | uncharacterized LOC100501753 |
| LOC103635561 | GRMZM2G175480 | | 13 | 14 | 70 | 45 | uncharacterized LOC103635561, transcript variant X1 |
| LOC103638673 | GRMZM2G026143 | | 36 | 18 | 24 | 79 | uncharacterized LOC103638673 |
| LOC103629419 | GRMZM2G069335 | | 20 | 12 | 17 | 45 | hypothetical protein |

**^a^**Maize genes are based on NCBI Id.

**^b^**Annotation of maize genes are obtained from NCBI database**.**

## Supplementary Table 9. The common genes that are differentially expressed in all interaction-time combinations with their

## respective fold change compared to mock in sorghum.

| **Sorghum Genes^a^** | **Fold change compared to mock** | | | |  | **Arabidopsis annotation^c^** |
| --- | --- | --- | --- | --- | --- | --- |
|  | **Compatible at 24 hai** | **Incompatible at 24 hai** | **Compatible at 72 hai** | **Incompatible at 72 hai** | **Product^b^** |  |
| Sobic.008G182700 | 2954 | 2194 | 725 | 2392 | similar to Thaumatin-like pathogenesis-related protein 4 precursor | osmotin 34 |
| Sobic.010G020200 | 925 | 569 | 266 | 1846 | similar to Pathogenesis-related protein PRMS precursor | pathogenesis-related gene 1 |
| Sobic.008G183300 | 1085 | 680 | 292 | 1367 | similar to Pathogenesis related protein-5 | osmotin 34 |
| Sobic.008G182600 | 3758 | 2626 | 153 | 1143 | similar to Pathogenesis related protein-5 | osmotin 34 |
| Sobic.002G023300 | 1288 | 574 | 57 | 305 | similar to Pathogenesis related protein-1 | pathogenesis-related gene 1 |
| Sobic.005G169400 | 263 | 160 | 33 | 180 | similar to Pathogenesis-related protein | pathogenesis-related 4 |
| Sobic.001G145700 | 332 | 137 | 20 | 170 | similar to Pathogenesis-related thaumatin-like protein | osmotin 34 |
| Sobic.002G351400 | 315 | 156 | 23 | 134 | similar to Zeamatin precursor | osmotin 34 |
| Sobic.008G182300 | 398 | 139 | 7 | 71 | similar to Thaumatin-like pathogenesis-related protein 4 precursor | osmotin 34 |
| Sobic.008G182800 | 111 | 60 | 18 | 64 | similar to Thaumatin-like pathogenesis-related protein 4 precursor | osmotin 34 |
| Sobic.008G182400 | 61 | 31 | 9 | 45 | similar to Thaumatin-like protein, putative, expressed | osmotin 34 |
| Sobic.008G182500 | 27 | 16 | 8 | 30 | similar to Thaumatin-like protein, putative, expressed | osmotin 34 |
| Sobic.001G401300 | 82 | 62 | 51 | 255 | similar to Pathogenesis-related protein 10a | PYR1-like 12 |
| Sobic.001G400900 | 210 | 141 | 8 | 22 | similar to Pathogenesis-related protein 10a | PYR1-like 6 |
| Sobic.001G400700 | 87 | 69 | 48 | 265 | NA**^d^** | PYR1-like 6 |
| Sobic.001G401200 | 55 | 47 | 17 | 65 | similar to Pathogenesis-related protein 10a | PYR1-like 6 |
| Sobic.001G400800 | 55 | 48 | 21 | 125 | NA | PYR1-like 6 |
| Sobic.005G101600 | 67 | 38 | 12 | 51 | NA | Disease resistance-responsive (dirigent-like protein) family protein |
| Sobic.005G101800 | 97 | 55 | 18 | 53 | NA | Disease resistance-responsive (dirigent-like protein) family protein |
| Sobic.009G012900 | 16 | 8 | 8 | 26 | weakly similar to HcrVf1 protein-like | disease resistance family protein / LRR family protein |
| Sobic.004G028600 | 8 | 7 | 10 | 54 | weakly similar to Putative LRR receptor-like kinase | Protein kinase family protein with leucine-rich repeat domain |
| Sobic.003G215800 | 9 | 8 | 5 | 15 | similar to Pleiotropic drug resistance protein 3 | pleiotropic drug resistance 12 |
| Sobic.003G216232 | 29 | 25 | 10 | 38 | similar to Pleiotropic drug resistance protein 3 | pleiotropic drug resistance 12 |
| Sobic.001G280000 | 3 | 4 | 3 | 8 | similar to Serine carboxypeptidase family protein, expressed | serine carboxypeptidase-like 6 |
| Sobic.003G111200 | 25 | 15 | 17 | 28 | NA | serine-type endopeptidase inhibitors |
| Sobic.004G191000 | 201 | 192 | 4 | 14 | similar to Os02g0578100 protein | UDP-glucosyl transferase 85A2 |
| Sobic.005G071300 | 4 | 3 | 3 | 5 | NA | UDP-Glycosyltransferase superfamily protein |
| Sobic.007G059100 | 52 | 20 | 46 | 121 | similar to Os12g0240900 protein | O-methyltransferase 1 |
| Sobic.007G058800 | 171 | 126 | 79 | 376 | similar to Os12g0240900 protein | O-methyltransferase 1 |
| Sobic.010G231000 | 131 | 70 | 31 | 138 | NA | O-methyltransferase family protein |
| Sobic.001G354400 | 270 | 123 | 32 | 38 | NA | O-methyltransferase family protein |
| Sobic.010G230800 | 219 | 82 | 42 | 178 | weakly similar to O-methyltransferase ZRP4 | O-methyltransferase family protein |
| Sobic.005G137000 | 269 | 210 | 8 | 64 | similar to Chalcone synthase WHP1 | Chalcone and stilbene synthase family protein |
| Sobic.007G058900 | 98 | 35 | 101 | 146 | similar to Chalcone synthase 8 | Chalcone and stilbene synthase family protein |
| Sobic.005G136300 | 83 | 50 | 15 | 109 | similar to Chalcone synthase WHP1 | Chalcone and stilbene synthase family protein |
| Sobic.005G137200 | 216 | 159 | 20 | 146 | similar to Chalcone synthase WHP1 | Chalcone and stilbene synthase family protein |
| Sobic.008G030100 | 9 | 7 | 4 | 13 | similar to Chalcone-flavanone isomerase family protein, expressed | Chalcone-flavanone isomerase family protein |
| Sobic.008G146700 | 26 | 11 | 11 | 45 | similar to Endo-beta-1,3-glucanase | beta-1,3-glucanase 2 |
| Sobic.003G422200 | 65 | 29 | 7 | 76 | NA | beta-1,3-glucanase 2 |
| Sobic.007G011700 | 13 | 10 | 6 | 23 | similar to Putative Bile acid beta-glucosidase | Beta-glucosidase, GBA2 type family protein |
| Sobic.006G132500 | 71 | 37 | 21 | 111 | similar to Endochitinase A precursor | homolog of carrot EP3-3 chitinase |
| Sobic.006G132700 | 27 | 20 | 7 | 20 | similar to Endochitinase A precursor | homolog of carrot EP3-3 chitinase |
| Sobic.006G132400 | 70 | 58 | 57 | 517 | similar to Chitinase-B1 | homolog of carrot EP3-3 chitinase |
| Sobic.003G244600 | 59 | 32 | 18 | 105 | NA | chitinase A |
| Sobic.010G230900 | 112 | 57 | 24 | 46 | similar to Putative uncharacterized protein | cytochrome P450, family 71, subfamily A, polypeptide 25 |
| Sobic.002G000400 | 18 | 16 | 5 | 12 | similar to Putative uncharacterized protein | cytochrome P450, family 93, subfamily D, polypeptide 1 |
| Sobic.003G031100 | 31 | 33 | 12 | 57 | weakly similar to Basic helix-loop-helix protein-like | basic helix-loop-helix (bHLH) DNA-binding family protein |
| Sobic.006G041700 | 7 | 5 | 4 | 18 | similar to H0502B11.10 protein | NAD(P)-binding Rossmann-fold superfamily protein |
| Sobic.006G226800 | 135 | 71 | 48 | 100 | similar to OSJNBb0015D13.3 protein | NAD(P)-binding Rossmann-fold superfamily protein |
| Sobic.003G422000 | 6 | 4 | 5 | 29 | similar to Endo-1,3-beta-glucanase | Glycosyl hydrolase superfamily protein |
| Sobic.002G420800 | 11 | 14 | 7 | 17 | NA | hydroxyproline-rich glycoprotein family protein |
| Sobic.001G416800 | 9 | 8 | 7 | 13 | similar to Histone H2A | histone H2A 12 |
| Sobic.003G411200 | 6 | 3 | 4 | 6 | similar to Sec14 like protein | Sec14p-like phosphatidylinositol transfer family protein |
| Sobic.005G126200 | 642 | 678 | 204 | 657 | similar to Leucine-rich repeat-containing extracellular glycoprotein precursor | somatic embryogenesis receptor-like kinase 2 |
| Sobic.008G191600 | 6 | 4 | 5 | 17 | similar to CDS_VAMP | vesicle-associated membrane protein 726 |
| Sobic.002G021132 | 4 | 5 | 3 | 12 | NA | phytosulfokine 4 precursor |
| Sobic.001G119000 | 4 | 5 | 7 | 32 | similar to Plastocyanin-like domain, putative | plantacyanin |
| Sobic.009G241700 | 3 | 3 | 3 | 11 | similar to Putative uncharacterized protein | 2-oxoglutarate (2OG) and Fe(II)-dependent oxygenase superfamily protein |
| Sobic.007G145600 | 30 | 24 | 8 | 25 | similar to Putative uncharacterized protein | 4-coumarate:CoA ligase 2 |
| Sobic.004G165200 | 12 | 10 | 5 | 10 | similar to Hydrolase, alpha/beta fold protein-like | alpha/beta-Hydrolases superfamily protein |
| Sobic.007G001100 | 6 | 4 | 4 | 14 | similar to Putative uncharacterized protein | Arabidopsis thaliana protein of unknown function (DUF821) |
| Sobic.010G128000 | 45 | 17 | 15 | 65 | similar to Putative uncharacterized protein | Eukaryotic aspartyl protease family protein |
| Sobic.010G177800 | 40 | 40 | 66 | 85 | NA | NA |
| Sobic.006G132600 | 4 | 3 | 3 | 6 | NA | NA |
| Sobic.006G078400 | 15 | 7 | 5 | 16 | similar to Putative uncharacterized protein | NA |
| Sobic.007G068500 | 49 | 48 | 22 | 52 | similar to Polyphenol oxidase | NA |
| Sobic.005G215900 | 101 | 69 | 24 | 78 | similar to Putative Bowman-Birk serine protease inhibitor | NA |
| Sobic.005G131400 | 39 | 26 | 13 | 73 | NA | NA |

^a^The sorghum orthologs id is based on Phytozome v12 id.

^b^Sorghum annotations are obtained from phytozome database

^c^Arabidopsis annotation represents the annotation of Arabidopsis orthologs and are used to infer about the unannotated maize and sorghum orthologs.

^d^NA indicates ‘not available’.

# Supplementary Table 10. The DEGs that are unique to incompatible interaction at 72 hai in maize.

| **Maize Genes** | | **Fold Change** | **FDR** | **Product^a^** |
| --- | --- | --- | --- | --- |
| **ENTREZ ID** | **Gramene Id** |  |  |  |
| LOC100191617 | GRMZM2G372068 | 144.128 | 0.0385 | hydroquinone glucosyltransferase |
| LOC100274269 | GRMZM2G127251 | 138.707 | 0.0058 | hydroxycinnamoyltransferase3 |
| LOC103654549 | GRMZM2G036365 | 114.571 | 0.0133 | aspartic proteinase nepenthesin-1 |
| pco103560(319) | GRMZM2G039639 | 109.283 | 0.0122 | Protein P21 |
| umc2600 | GRMZM2G170017 | 65.727 | 0.0175 | (+)-neomenthol dehydrogenase |
| IDP2565 | GRMZM2G112538 | 61.461 | 0.0116 | uncharacterized LOC100192117 |
| LOC100502520 | GRMZM2G093826 | 50.398 | 0.0099 | potassium high-affinity transporter |
| LOC541815 | GRMZM2G067402 | 50.227 | 0.0448 | hemoglobin |
| LOC103636584 | GRMZM2G440003 | 46.466 | 0.0153 | salicylic acid-binding protein 2 |
| aox3 | GRMZM2G074743 | 44.472 | 0.0104 | alternative oxidase AOX3 precursor |
| sip1 | GRMZM2G374971 | 44.358 | 0.0093 | stress-induced protein 1 |
| LOC103628972 | GRMZM2G476040 | 42.616 | 0.0149 | uncharacterized LOC103628972 |
| LOC103627644 | GRMZM2G028397 | 42.429 | 0.0489 | AT-hook motif nuclear-localized protein 25, transcript variant X2 |
| LOC103654463 | GRMZM2G301148 | 35.247 | 0.0028 | UDP-glycosyltransferase 89B2 |
| LOC103633335 | GRMZM2G443843 | 33.271 | 0.0118 | L-type lectin-domain containing receptor kinase IV.1 |
| LOC103626437 | GRMZM2G050450 | 33.076 | 0.0169 | benzyl alcohol O-benzoyltransferase |
| LOC103638616 | GRMZM2G340177 | 32.996 | 0.0180 | putative transcription factor bHLH041 |
| LOC103646447 | GRMZM2G131177 | 32.559 | 0.0148 | subtilisin-like protease SBT1.7 |
| LOC103651266 | GRMZM2G101405 | 32.176 | 0.0419 | probable WRKY transcription factor 51 |
| LOC103628961 | GRMZM2G015793 | 31.804 | 0.0122 | uncharacterized LOC103628961 |
| LOC100278342 | no_grmzm_overlap | 31.117 | 0.0140 | uncharacterized LOC100278342 |
| LOC109943001 | no_grmzm_overlap | 30.989 | 0.0453 | putative F-box/LRR-repeat protein 23 |
| pco091453a | GRMZM2G112524 | 29.751 | 0.0095 | uncharacterized LOC100284195 |
| LOC100281430 | GRMZM2G169966 | 29.493 | 0.0135 | WRKY70 - superfamily of TFs having WRKY and zinc finger domains |
| LOC100275543 | AC214817.3_FG007 | 28.860 | 0.0140 | uncharacterized LOC100275543, transcript variant X1 |
| LOC103629700 | AC209050.3_FG003 | 27.076 | 0.0196 | WRKY transcription factor WRKY28 |
| LOC103643717 | no_grmzm_overlap | 26.607 | 0.0218 | uncharacterized LOC103643717 |
| LOC100280265 | GRMZM2G179792 | 26.441 | 0.0301 | phospholipase D family protein, transcript variant X1 |
| LOC100285450 | GRMZM2G074611 | 26.080 | 0.0337 | dirigent |
| LOC100191720 | GRMZM2G099297 | 25.316 | 0.0331 | 5-pentadecatrienyl resorcinol O-methyltransferase |
| LOC103647804 | GRMZM2G079082 | 24.708 | 0.0329 | putative disease resistance RPP13-like protein 1 |
| LOC100384448 | GRMZM2G449094 | 23.747 | 0.0110 | uncharacterized LOC100384448 |
| LOC109939432 | no_grmzm_overlap | 22.704 | 0.0239 | auxin-responsive protein SAUR40 |
| LOC100279719 | GRMZM2G073884 | 22.668 | 0.0257 | Putative leucine-rich repeat receptor-like protein kinase family protein, transcript variant X1 |
| LOC103634934 | GRMZM2G333582 | 22.148 | 0.0266 | putative transcription factor bHLH041 |
| LOC103638821 | GRMZM2G111711 | 22.098 | 0.0218 | WRKY transcription factor WRKY28 |
| LOC100277767 | GRMZM2G039362 | 21.702 | 0.0099 | uncharacterized LOC100277767 |
| pco093477 | no_grmzm_overlap | 20.294 | 0.0104 | Protein MID1-COMPLEMENTING ACTIVITY 1 |
| LOC103641905 | GRMZM2G059012 | 19.922 | 0.0177 | wall-associated receptor kinase 5, transcript variant X2 |
| LOC103641623 | GRMZM2G014022 | 19.855 | 0.0266 | probable F-box protein At2g36090 |
| LOC103629079 | GRMZM5G856011 | 19.557 | 0.0331 | cysteine-rich receptor-like protein kinase 6 |
| LOC107548101 | GRMZM2G029243 | 19.455 | 0.0410 | uncharacterized LOC107548101 |
| LOC100272926 | GRMZM2G003409 | 19.453 | 0.0275 | hydroxyproline-rich glycoprotein family protein, transcript variant X1 |
| LOC100282558 | no_grmzm_overlap | 19.370 | 0.0068 | SAUR20 - auxin-responsive SAUR family member |
| LOC100192463 | no_grmzm_overlap | 19.259 | 0.0235 | uncharacterized LOC100192463 |
| TIDP2910 | GRMZM2G041068 | 17.917 | 0.0420 | uncharacterized LOC100275684 |
| TIDP2786 | GRMZM2G402977 | 17.812 | 0.0222 | uncharacterized LOC100278114, transcript variant X1 |
| TIDP3317 | GRMZM2G176307 | 16.563 | 0.0311 | uncharacterized LOC100037774 |
| LOC103635704 | GRMZM2G382035 | 16.561 | 0.0122 | hypothetical protein |
| LOC103631816 | GRMZM2G061806 | 16.247 | 0.0379 | tryptamine benzoyltransferase 1 |
| LOC100273169 | GRMZM2G141376 | 16.100 | 0.0233 | Plant L-ascorbate oxidase |
| LOC103633265 | GRMZM2G379780 | 15.797 | 0.0124 | cysteine-rich receptor-like protein kinase 10 |
| LOC103630785 | GRMZM2G163883 | 15.595 | 0.0370 | PLASMODESMATA CALLOSE-BINDING PROTEIN 2, transcript variant X2 |
| LOC100278150 | GRMZM2G078667 | 15.196 | 0.0242 | uncharacterized LOC100278150 |
| LOC103648003 | GRMZM2G106560 | 15.161 | 0.0387 | probable WRKY transcription factor 45 |
| LOC103650969 | GRMZM2G074248 | 14.893 | 0.0421 | NDR1/HIN1-like protein 10 |
| mfsd1 | GRMZM2G161310 | 14.785 | 0.0240 | major facilitator superfamily defense 1, transcript variant X2 |
| LOC100274125 | GRMZM2G361256 | 14.235 | 0.0201 | uncharacterized LOC100274125, transcript variant X2 |
| LOC103630405 | GRMZM2G000836 | 13.855 | 0.0104 | protein TRANSPARENT TESTA 1 |
| IDP820 | GRMZM2G153208 | 13.777 | 0.0162 | Secretory protein |
| LOC103634550 | GRMZM2G475971 | 13.776 | 0.0430 | Multidrug resistance protein ABC transporter family protein |
| LOC100384165 | GRMZM2G164182 | 13.697 | 0.0113 | Alpha carbonic anhydrase 1 chloroplastic |
| LOC542630 | GRMZM2G150474 | 13.686 | 0.0157 | glutathione S-transferase GST 15, transcript variant X1 |
| LOC103654516 | GRMZM2G327659 | 13.486 | 0.0183 | putative disease resistance protein RGA3 |
| LOC103632613 | GRMZM2G011526 | 13.431 | 0.0366 | probable LRR receptor-like serine/threonine-protein kinase At1g07650, transcript variant X3 |
| LOC103649549 | GRMZM2G170047 | 13.414 | 0.0414 | cytochrome P450 71A1 |
| LOC100281688 | no_grmzm_overlap | 13.317 | 0.0179 | 6-phosphofructokinase 2 |
| LOC103654518 | GRMZM2G079436 | 13.269 | 0.0383 | OSJNBb0045P24.5-like protein |
| LOC103632803 | AC208897.3_FG004 | 13.190 | 0.0337 | sugar transport protein 13 |
| LOC100382329 | GRMZM2G320023 | 13.167 | 0.0217 | disease resistance response protein 206 |
| LOC103651474 | GRMZM2G011347 | 12.746 | 0.0261 | S-type anion channel SLAH2, transcript variant X3 |
| LOC109940736 | no_grmzm_overlap | 12.611 | 0.0155 | uncharacterized LOC109940736 |
| LOC100284471 | GRMZM2G322661 | 12.510 | 0.0369 | DNA-directed RNA polymerases I, II, and III 7.3 kDa polypeptide |
| LOC103655092 | GRMZM2G419675 | 12.391 | 0.0224 | barwin |
| LOC103649888 | GRMZM2G103748 | 12.331 | 0.0153 | uncharacterized LOC103649888 |
| LOC109939245 | GRMZM2G131442 | 12.073 | 0.0329 | transcription factor MYB108-like |
| LOC100382682 | GRMZM2G177883 | 11.928 | 0.0218 | Protein kinase family protein with leucine-rich repeat domain |
| LOC103646881 | GRMZM2G076394 | 11.858 | 0.0213 | protein REDUCED WALL ACETYLATION 4, transcript variant X1 |
| cl244_1 | GRMZM2G093125 | 11.647 | 0.0350 | uncharacterized LOC100285936 |
| LOC103637622 | GRMZM2G046848 | 11.501 | 0.0372 | U-box domain-containing protein 70, transcript variant X2 |
| LOC109943524 | no_grmzm_overlap | 11.383 | 0.0301 | WAT1-related protein At5g64700, transcript variant X1 |
| LOC100501931 | GRMZM2G405662 | 11.285 | 0.0335 | Heavy metal transport/detoxification superfamily protein |
| LOC100286051 | GRMZM2G160710 | 11.262 | 0.0312 | ATFP4 |
| LOC103627472 | GRMZM2G170016 | 11.078 | 0.0122 | membrane steroid-binding protein 1 |
| LOC100277616 | GRMZM2G178852 | 11.061 | 0.0431 | uncharacterized LOC100277616 |
| LOC541731 | GRMZM2G160840 | 11.019 | 0.0303 | transcription factor JAMYB |
| LOC100127507 | GRMZM2G052100 | 11.000 | 0.0076 | Asr protein |
| pco153521(653) | GRMZM2G029087 | 10.995 | 0.0317 | uncharacterized LOC100272936 |
| LOC100272903 | GRMZM2G175140 | 10.923 | 0.0212 | ammonium transporter1 |
| LOC100501883 | GRMZM2G465999 | 10.877 | 0.0211 | putative S-locus receptor-like protein kinase family protein, transcript variant X1 |
| LOC103642144 | GRMZM2G007477 | 10.598 | 0.0157 | G-type lectin S-receptor-like serine/threonine-protein kinase SD2-5, transcript variant X6 |
| LOC100284733 | GRMZM2G138370 | 10.570 | 0.0485 | VQ motif family protein |
| LOC103630625 | AC206165.3_FG007 | 10.565 | 0.0212 | heat stress transcription factor A-4d, transcript variant X2 |
| TIDP2766 | no_grmzm_overlap | 10.269 | 0.0438 | uncharacterized LOC100272682 |
| LOC103630227 | GRMZM2G447480 | 10.261 | 0.0272 | uncharacterized LOC103630227 |
| LOC103630536 | GRMZM2G140752 | 10.256 | 0.0430 | putative receptor-like protein kinase |
| LOC103637786 | GRMZM5G855994 | 10.129 | 0.0120 | U-box domain-containing protein 21 |
| LOC103647555 | GRMZM2G008374 | 10.084 | 0.0248 | protein CUP-SHAPED COTYLEDON 1 |
| LOC100191997 | GRMZM2G048522 | 10.061 | 0.0422 | putative AMP-dependent synthetase and ligase superfamily protein, transcript variant X1 |
| LOC103640852 | GRMZM2G076593 | 9.399 | 0.0257 | amino acid permease 3 |
| LOC103630640 | GRMZM2G132212 | 9.394 | 0.0120 | receptor-like protein kinase HSL1 |
| LOC110117500 | NA | 9.180 | 0.0331 | uncharacterized LOC110117500 |
| LOC103650987 | GRMZM2G154958 | 9.175 | 0.0355 | GABA transporter 1 |
| gpm263 | GRMZM2G066067 | 9.077 | 0.0422 | UDP-glycosyltransferase 91A1, transcript variant X1 |
| LOC103636404 | GRMZM2G470442 | 9.047 | 0.0431 | cytochrome P450 78A5 |
| LOC100281825 | GRMZM2G099454 | 9.017 | 0.0054 | basic endochitinase C |
| LOC103634498 | GRMZM2G459824 | 8.966 | 0.0412 | mitogen-activated protein kinase kinase kinase 18 |
| LOC100285572 | GRMZM2G056467 | 8.916 | 0.0309 | calmodulin binding protein |
| LOC103651322 | GRMZM2G448710 | 8.845 | 0.0080 | LEAF RUST 10 DISEASE-RESISTANCE LOCUS RECEPTOR-LIKE PROTEIN KINASE-like 2.1 |
| LOC103631582 | GRMZM2G116335 | 8.793 | 0.0370 | putative disease resistance protein RGA3 |
| LOC103647857 | GRMZM2G125004 | 8.788 | 0.0097 | F-box only protein 13 |
| LOC100273946 | GRMZM2G029566 | 8.747 | 0.0166 | hypothetical protein |
| cl31612_1 | GRMZM2G178875 | 8.431 | 0.0435 | uncharacterized LOC100284129 |
| LOB14 | GRMZM2G044902 | 8.328 | 0.0118 | LOB transcription factor |
| LOC103627642 | GRMZM2G466298 | 8.328 | 0.0460 | probable L-type lectin-domain containing receptor kinase S.5, transcript variant X2 |
| mstr1 | GRMZM2G135739 | 8.256 | 0.0484 | monosaccharide transporter 1 |
| umc2526 | GRMZM2G132489 | 8.173 | 0.0468 | Yucca5, transcript variant X1 |
| LOC100281606 | GRMZM5G832149 | 8.093 | 0.0124 | WAK2 - OsWAK receptor-like cytoplasmic kinase (OsWAK-RLCK) |
| LOC103641692 | GRMZM2G092747 | 8.090 | 0.0070 | protein LURP-one-related 6, transcript variant X2 |
| LOC100282611 | GRMZM5G834303 | 8.061 | 0.0276 | cytokinin-O-glucosyltransferase 2 |
| LOC103626938 | GRMZM2G449019 | 7.806 | 0.0052 | anthocyanidin 5,3-O-glucosyltransferase |
| LOC100273396 | GRMZM2G053284 | 7.660 | 0.0355 | Actin-7, transcript variant X1 |
| LOC100275790 | GRMZM2G116640 | 7.569 | 0.0158 | uncharacterized LOC100275790 |
| LOC103649885 | GRMZM2G177991 | 7.540 | 0.0346 | hypothetical protein |
| LOC103628663 | GRMZM2G016434 | 7.517 | 0.0274 | ethylene-responsive transcription factor ABI4, transcript variant X1 |
| LOC103635071 | GRMZM2G004519 | 7.498 | 0.0098 | E3 ubiquitin-protein ligase EL5 |
| LOC100279755 | GRMZM2G172396 | 7.349 | 0.0157 | PR5-like receptor kinase, transcript variant X2 |
| LOC100281560 | GRMZM2G355381 | 7.254 | 0.0192 | type I inositol-1,4,5-trisphosphate 5-phosphatase CVP2 |
| LOC103627656 | GRMZM2G459110 | 7.073 | 0.0422 | hypothetical protein |
| LOC100281069 | GRMZM2G159587 | 7.057 | 0.0433 | glyoxylate reductase |
| AUX36 | GRMZM2G359924 | 6.978 | 0.0472 | IAA16-auxin-responsive Aux/IAA family member |
| LOC100273835 | GRMZM2G018108 | 6.976 | 0.0266 | Aspartyl protease AED1 |
| LOC103636604 | GRMZM2G132978 | 6.925 | 0.0377 | putative cysteine-rich receptor-like protein kinase 35 |
| LOC100273458 | GRMZM2G166639 | 6.887 | 0.0088 | uncharacterized LOC100273458 |
| LOC103650557 | GRMZM2G169562 | 6.695 | 0.0262 | Mono-/di-acylglycerol lipase N-terminal |
| LOC103640562 | GRMZM2G350793 | 6.675 | 0.0425 | probable LRR receptor-like serine/threonine-protein kinase At3g47570 |
| LOC100279462 | GRMZM2G032766 | 6.595 | 0.0045 | Calcium-dependent lipid-binding (CaLB domain) family protein |
| LOC103652212 | GRMZM2G163507 | 6.500 | 0.0349 | putative disease resistance RPP13-like protein 1, transcript variant X1 |
| LOC103633617 | GRMZM2G086628 | 6.469 | 0.0361 | mavicyanin |
| LOC103625710 | AC190636.3_FG005 | 6.389 | 0.0235 | S-norcoclaurine synthase 1 |
| LOC100274029 | GRMZM2G020098 | 6.360 | 0.0097 | hypothetical protein, transcript variant X1 |
| LOC100191493 | GRMZM2G104655 | 6.166 | 0.0198 | putative lectin-domain receptor-like protein kinase family protein |
| WRKY29 | GRMZM2G040298 | 6.144 | 0.0076 | putative WRKY DNA-binding domain superfamily protein |
| LOC103651594 | GRMZM2G143139 | 6.135 | 0.0077 | ABC transporter G family member 37, transcript variant X1 |
| IDP2562 | GRMZM2G321239 | 6.092 | 0.0349 | calcium dependent protein kinase4 |
| LOC100217128 | GRMZM2G033515 | 5.931 | 0.0439 | seed maturation protein PM41 |
| LOC103627122 | GRMZM2G011160 | 5.674 | 0.0448 | ABC transporter A family member 10, transcript variant X1 |
| LOC100283948 | GRMZM2G139535 | 5.596 | 0.0247 | heat shock factor protein 4 |
| LOC103634995 | no_grmzm_overlap | 5.594 | 0.0100 | pathogenesis-related genes transcriptional activator PTI6 |
| LOC100382469 | GRMZM2G004858 | 5.576 | 0.0475 | UDP-glycosyltransferase 87A1, transcript variant X2 |
| LOC107305676 | AC233853.1_FG003 | 5.560 | 0.0218 | Cytochrome P450 710A1 |
| LOC103650192 | no_grmzm_overlap | 5.537 | 0.0266 | DELLA protein SLN1-like |
| AY110632 | GRMZM2G032047 | 5.521 | 0.0068 | uncharacterized LOC100283541 |
| LOC103642112 | GRMZM2G085236 | 5.512 | 0.0351 | ABC transporter B family member 19-like |
| LOC100285445 | GRMZM2G043799 | 5.304 | 0.0369 | nodulation receptor kinase, transcript variant X1 |
| LOC103630568 | GRMZM2G001332 | 5.290 | 0.0380 | uncharacterized LOC103630568 |
| LOC100275164 | no_grmzm_overlap | 5.261 | 0.0341 | dof zinc finger protein 5, transcript variant X3 |
| LOC100275806 | GRMZM2G035248 | 5.038 | 0.0422 | hypothetical protein |
| LOC103638817 | GRMZM2G452896 | 5.015 | 0.0231 | lysosomal Pro-X carboxypeptidase |
| LOC100501408 | GRMZM2G502350 | 4.947 | 0.0350 | uncharacterized LOC100501408 |
| LOC103639007 | GRMZM2G431243 | 4.672 | 0.0313 | calmodulin-binding transcription activator 1, transcript variant X2 |
| IDP692 | GRMZM2G349187 | 4.669 | 0.0155 | uncharacterized LOC100384864 |
| LOC100281202 | GRMZM2G304897 | 4.603 | 0.0162 | receptor-like protein kinase |
| LOC103641011 | GRMZM2G423472 | 4.575 | 0.0168 | vesicle-associated membrane protein 721, transcript variant X2 |
| LOC103628955 | GRMZM2G374827 | 4.528 | 0.0151 | non-lysosomal glucosylceramidase |
| LOC100280301 | GRMZM2G120587 | 4.390 | 0.0309 | Serine carboxypeptidase-like 51 |
| LOC100273562 | GRMZM2G003642 | 4.342 | 0.0013 | hypothetical protein |
| LOC100384255 | GRMZM2G021378 | 4.288 | 0.0257 | Syntaxin-132 |
| LOC100280886 | GRMZM2G439668 | 4.238 | 0.0168 | chaperone protein DnaJ |
| LOC542138 | GRMZM2G106548 | 4.182 | 0.0297 | scarecrow-like protein 23 |
| WPK1 | AC235547.1_FG005 | 4.155 | 0.0165 | wound and phytochrome signaling involved receptor like kinase |
| LOC103645450 | GRMZM2G532387 | 4.130 | 0.0073 | uncharacterized LOC103645450 |
| LOC100217133 | GRMZM2G073860 | 3.933 | 0.0068 | purple acid phosphatase |
| LOC100272302 | GRMZM2G026470 | 3.857 | 0.0430 | soluble inorganic pyrophosphatase |
| LOC100277555 | GRMZM2G076499 | 3.704 | 0.0218 | hypothetical protein |
| LOC100280259 | GRMZM2G364172 | 3.682 | 0.0226 | putative protein kinase superfamily protein, transcript variant X4 |
| LOC100275154 | GRMZM2G129019 | 3.675 | 0.0283 | uncharacterized LOC100275154 |
| si614068e02 | no_grmzm_overlap | 3.629 | 0.0298 | uncharacterized LOC100282272 |
| LOC103627070 | GRMZM2G176206 | 3.609 | 0.0196 | receptor-like protein kinase 5 |
| LOC100278276 | GRMZM2G049990 | 3.591 | 0.0421 | hypothetical protein |
| LOC100285801 | no_grmzm_overlap | 3.529 | 0.0157 | CDA1 |
| pco129120b | GRMZM2G006219 | 3.529 | 0.0040 | uncharacterized LOC100281331 |
| LOC100382321 | GRMZM2G025109 | 3.499 | 0.0266 | Cysteine/Histidine-rich C1 domain family protein |
| LOC100274255 | GRMZM2G051079 | 3.481 | 0.0266 | uncharacterized LOC100274255 |
| LOC100277671 | GRMZM2G132958 | 3.404 | 0.0340 | Regulator of Vps4 activity in the MVB pathway protein |
| LOC100283628 | GRMZM2G173882 | 3.344 | 0.0115 | myb-like DNA-binding domain, SHAQKYF class family protein |
| AY107726 | GRMZM2G433162 | 3.274 | 0.0329 | uncharacterized LOC100382310 |
| LOC103647314 | GRMZM2G074687 | 3.222 | 0.0209 | calreticulin-like |
| LOC100382449 | GRMZM2G118800 | 3.220 | 0.0330 | Aldehyde dehydrogenase |
| LOC100191951 | GRMZM2G113512 | 3.173 | 0.0336 | putative protein kinase superfamily protein, transcript variant X2 |
| TIDP3022 | GRMZM2G412986 | 3.115 | 0.0295 | Glycosyltransferase family 61 protein, transcript variant X1 |
| LOC100285108 | GRMZM2G141931 | 3.067 | 0.0351 | endoplasmin |
| LOC100281774 | GRMZM2G180082 | 3.066 | 0.0303 | taxane 13-alpha-hydroxylase |
| LOC100285697 | GRMZM2G076435 | 3.030 | 0.0438 | plant viral-response family protein |
| pco137126 | GRMZM2G053004 | 3.007 | 0.0165 | Vacuolar cation/proton exchanger 2, transcript variant X1 |
| LOC100275496 | GRMZM2G014441 | 2.960 | 0.0377 | hypothetical protein, transcript variant X1 |
| pco064611a | GRMZM2G080375 | 2.937 | 0.0218 | ATP-dependent 6-phosphofructokinase 4 chloroplastic, transcript variant 2 |
| LOC100280599 | GRMZM2G039725 | 2.839 | 0.0026 | S-ribonuclease binding protein SBP1, transcript variant X1 |
| LOC103641901 | GRMZM2G404367 | 2.794 | 0.0274 | GTPase activating protein 1 |
| LOC103625861 | GRMZM2G035103 | 2.773 | 0.0410 | zinc finger protein 1 |
| LOC100502435 | GRMZM2G029323 | 2.596 | 0.0412 | putative AP2/EREBP transcription factor superfamily protein |
| LOC103645768 | GRMZM2G068059 | 2.557 | 0.0158 | anamorsin homolog |
| LOC103634138 | GRMZM2G073275 | 2.545 | 0.0392 | uncharacterized LOC103634138 |
| LOC103642232 | GRMZM2G178025 | 2.535 | 0.0248 | endoglucanase 12-like |
| LOC100285658 | GRMZM2G012229 | 2.507 | 0.0466 | nitrate-induced NOI protein, transcript variant X3 |
| TIDP3699 | GRMZM2G065800 | 2.486 | 0.0134 | PLAC8 family protein, transcript variant X1 |
| EREB109 | GRMZM2G141679 | 2.408 | 0.0448 | ethylene-responsive transcription factor RAP2-13-like |
| LOC103648828 | GRMZM2G365957 | 2.397 | 0.0058 | ABC transporter C family member 4 |
| LOC100280415 | GRMZM2G164426 | 2.342 | 0.0266 | uncharacterized LOC100280415 |
| LOC100383884 | GRMZM2G071307 | 2.269 | 0.0422 | hypothetical protein |
| LOC100216722 | GRMZM2G036708 | 2.257 | 0.0212 | cysteine synthase, transcript variant X1 |
| LOC103634763 | GRMZM2G473511 | 2.156 | 0.0193 | rust resistance kinase Lr10, transcript variant X4 |
| LOC100282967 | GRMZM2G029863 | 2.140 | 0.0226 | protein binding protein, transcript variant 2 |
| LOC103640673 | GRMZM2G180254 | 2.004 | 0.0422 | putative disease resistance RPP13-like protein 3, transcript variant X2 |
| LOC100285772 | GRMZM2G111697 | -2.028 | 0.0462 | ACR4 |
| LOC100285566 | GRMZM2G166759 | -2.044 | 0.0268 | ubiquitin domain containing 1 |
| LOC103649905 | GRMZM2G051577 | -2.045 | 0.0215 | deoxynucleoside triphosphate triphosphohydrolase SAMHD1 homolog |
| LOC103630796 | GRMZM2G102255 | -2.101 | 0.0252 | probable protein phosphatase 2C 53, transcript variant X2 |
| LOC103651339 | GRMZM2G106028 | -2.110 | 0.0202 | 65-kDa microtubule-associated protein 3, transcript variant X1 |
| LOC103652969 | GRMZM2G080387 | -2.248 | 0.0100 | nucleobase-ascorbate transporter 2 |
| LOC103646008 | NA | -2.249 | 0.0346 | WEB family protein At5g16730, chloroplastic |
| LOC103626409 | GRMZM2G431573 | -2.368 | 0.0379 | ARM repeat superfamily protein, transcript variant X2 |
| LOC100383618 | GRMZM2G080270 | -2.484 | 0.0122 | putative leucine-rich repeat receptor-like protein kinase family protein |
| LOC103642170 | GRMZM2G423861 | -2.485 | 0.0181 | kinesin-like protein KIN-14I, transcript variant X1 |
| THX3 | GRMZM2G415229 | -2.527 | 0.0170 | trihelix transcription factor |
| umc1285 | GRMZM2G050861 | -2.547 | 0.0327 | Protein kinase superfamily protein |
| LOC100284635 | GRMZM2G082257 | -2.595 | 0.0013 | YGL010w-like protein, transcript variant X1 |
| LOC103653907 | GRMZM2G301585 | -2.598 | 0.0077 | RING-H2 finger protein ATL72 |
| LOC100193630 | GRMZM2G314692 | -2.716 | 0.0483 | uncharacterized LOC100193630, transcript variant X2 |
| umc1460 | GRMZM2G035465 | -2.944 | 0.0079 | Auxin-responsive protein IAA27, transcript variant X2 |
| LOC103648084 | GRMZM2G433731 | -3.006 | 0.0218 | uncharacterized LOC103648084, transcript variant X1 |
| LOC100273750 | GRMZM2G150266 | -3.035 | 0.0345 | uncharacterized LOC100273750 |
| LOC103643310 | GRMZM2G169198 | -3.074 | 0.0470 | Retinol dehydrogenase 13 |
| LOC100381516 | GRMZM2G419290 | -3.123 | 0.0281 | uncharacterized LOC100381516, transcript variant 2 |
| LOC100280003 | GRMZM2G034534 | -3.173 | 0.0453 | uncharacterized LOC100280003 |
| LOC100274751 | GRMZM2G110558 | -3.174 | 0.0290 | UDP-glucuronate 4-epimerase 1 |
| LOC100383455 | GRMZM5G821267 | -3.475 | 0.0218 | U-box domain-containing protein 7, transcript variant X1 |
| LOC100281123 | GRMZM2G363520 | -3.550 | 0.0351 | RING-H2 finger protein ATL2L |
| LOC100279240 | GRMZM2G061734 | -3.622 | 0.0350 | Squamosa promoter-binding-like protein 6, transcript variant X1 |
| LOC100281337 | GRMZM2G384528 | -3.680 | 0.0128 | nuclear transcription factor Y subunit B-3 |
| LOC103636515 | GRMZM2G112836 | -3.690 | 0.0492 | protein UPSTREAM OF FLC, transcript variant X2 |
| LOC109945603 | no_grmzm_overlap | -3.706 | 0.0068 | PAN domain-containing protein At5g03700 |
| LOC100284799 | GRMZM2G407044 | -3.755 | 0.0223 | acetolactate synthase/ amino acid binding protein |
| LOC100275189 | no_grmzm_overlap | -3.905 | 0.0495 | uncharacterized LOC100275189 |
| LOC100217198 | GRMZM2G160013 | -4.677 | 0.0297 | ELMO/CED-12 family protein, transcript variant X2 |
| LOC100281823 | GRMZM2G073359 | -5.721 | 0.0218 | protein kinase APK1B |
| LOC103633554 | GRMZM2G025182 | -5.959 | 0.0266 | pectinesterase |
| LOC100280813 | GRMZM2G062673 | -6.136 | 0.0082 | calmodulin |
| LOC100381304 | NA | -6.553 | 0.0113 | uncharacterized LOC100381304 |
| LOC103632254 | GRMZM2G100828 | -7.270 | 0.0262 | alpha-glucosidase 2 |
| magi113703 | GRMZM2G115635 | -7.546 | 0.0149 | uncharacterized LOC100273531 |
| LOC100194073 | GRMZM2G173926 | -7.786 | 0.0157 | uncharacterized LOC100194073 |
| LOC100192077 | GRMZM2G321041 | -8.198 | 0.0012 | putative RING zinc finger domain superfamily protein |
| LOC111591320 | NA | -9.534 | 0.0011 | uncharacterized LOC111591320, transcript variant X2 |
| LOC100277241 | GRMZM2G456422 | -11.196 | 0.0079 | OSJNBb0048E02.16-like protein |
| saf1 | GRMZM2G042639 | -11.793 | 0.0337 | safener induced 1, transcript variant X2 |
| LOC100384085 | GRMZM2G122873 | -11.998 | 0.0262 | uncharacterized LOC100384085 |
| LOC103631691 | GRMZM2G172751 | -12.217 | 0.0194 | hypothetical protein |
| LOC100384311 | GRMZM2G076584 | -15.484 | 0.0466 | uncharacterized LOC100384311, transcript variant X1 |
| LOC103633203 | GRMZM2G395508 | -17.547 | 0.0369 | anthocyanidin 3-O-glucosyltransferase 2 |
| LOC100277221 | GRMZM2G058208 | -21.912 | 0.0024 | Os07g0585250-like protein |
| LOC100383045 | GRMZM2G103197 | -27.115 | 0.0302 | uncharacterized LOC100383045 |
| LOC100285459 | GRMZM2G058173 | -47.257 | 0.0099 | undecaprenyl pyrophosphate synthetase |

**^a^**Annotation of maize genes are obtained from NCBI database**.**

# Supplementary Table 11. The DEGs that are unique to incompatible interaction 72 hai in sorghum.

| **Sorghum Genes** | **Fold Change** | **FDR** | **Arabidopsis annotation** |
| --- | --- | --- | --- |
| Sobic.005G024100 | 90.32 | 0.00155 | Rhamnogalacturonate lyase family protein |
| Sobic.001G119100 | 82.35 | 0.00083 | plantacyanin |
| Sobic.006G005500 | 70.75 | 0.02588 | Zinc finger (C3HC4-type RING finger) family protein |
| Sobic.005G219000 | 66.82 | 0.0341 | PATATIN-like protein 4 |
| Sobic.003G164800 | 48.91 | 0.02759 | Glutathione S-transferase family protein |
| Sobic.007G198000 | 41.13 | 0.02057 | auxin-responsive family protein |
| Sobic.005G217700 | 38.52 | 0.00521 | Protein of Unknown Function (DUF239) |
| Sobic.001G410450 | 37.2 | 0.00622 | 0 |
| Sobic.001G381300 | 36.53 | 0.00542 | WRKY DNA-binding protein 70 |
| Sobic.003G097800 | 34.95 | 0.02114 | 0 |
| Sobic.001G318200 | 34.39 | 0.02473 | glutathione S-transferase TAU 18 |
| Sobic.002G195200 | 30.58 | 0.00159 | 0 |
| Sobic.002G327900 | 29.38 | 0.04303 | O-Glycosyl hydrolases family 17 protein |
| Sobic.008G055100 | 28.12 | 0.00848 | Disease resistance-responsive (dirigent-like protein) family protein |
| Sobic.007G214300 | 24.59 | 0.00712 | wall associated kinase 5 |
| Sobic.007G115500 | 24.39 | 0.01661 | Plant protein of unknown function (DUF247) |
| Sobic.001G123700 | 24.2 | 0.00446 | 0 |
| Sobic.005G065000 | 24.07 | 0.03668 | Leucine-rich repeat protein kinase family protein |
| Sobic.008G029300 | 23.99 | 0.01525 | 0 |
| Sobic.006G024200 | 23.14 | 0.01684 | receptor-like protein kinase 1 |
| Sobic.008G099300 | 22.98 | 0.0062 | cysteine-rich RLK (RECEPTOR-like protein kinase) 41 |
| Sobic.010G193100 | 22.34 | 0.00626 | 0 |
| Sobic.001G164900 | 22 | 0.0115 | UDP-Glycosyltransferase superfamily protein |
| Sobic.006G113800 | 21.78 | 0.01672 | Heavy metal transport/detoxification superfamily protein |
| Sobic.004G245200 | 21.29 | 0.00088 | 0 |
| Sobic.010G162000 | 20.97 | 0.04078 | Peroxidase superfamily protein |
| Sobic.010G265600 | 20.79 | 0.03104 | Protein of unknown function (DUF581) |
| Sobic.004G088551 | 20.25 | 0.04539 | 0 |
| Sobic.006G061300 | 19.62 | 0.03872 | high affinity K+ transporter 5 |
| Sobic.006G148900 | 19.54 | 0.02589 | PHE ammonia lyase 1 |
| Sobic.001G231600 | 19.1 | 0.01278 | NAD(P)-binding Rossmann-fold superfamily protein |
| Sobic.004G258500 | 18.98 | 0.01387 | Protein of unknown function (DUF506) |
| Sobic.003G039700 | 18.21 | 0.03695 | 2-oxoglutarate (2OG) and Fe(II)-dependent oxygenase superfamily protein |
| Sobic.010G061300 | 17.7 | 0.02096 | Leucine-rich repeat protein kinase family protein |
| Sobic.006G148500 | 17.53 | 0.02049 | wall associated kinase 5 |
| Sobic.004G112400 | 17.48 | 0.01684 | Plant protein of unknown function (DUF247) |
| Sobic.003G210601 | 17.04 | 0.00868 | disease resistance family protein / LRR family protein |
| Sobic.006G007600 | 16.78 | 0.04569 | 0 |
| Sobic.007G215700 | 16.36 | 0.00395 | Wall-associated kinase family protein |
| Sobic.010G054400 | 16.33 | 0.01672 | Leucine-rich repeat protein kinase family protein |
| Sobic.003G312300 | 16.08 | 0.0155 | homocysteine methyltransferase 2 |
| Sobic.009G171600 | 15.87 | 0.01132 | WRKY DNA-binding protein 33 |
| Sobic.001G339300 | 15.6 | 0.00446 | 0 |
| Sobic.002G249600 | 15.45 | 0.03777 | wall-associated kinase 2 |
| Sobic.001G331000 | 15.23 | 0.02097 | 0 |
| Sobic.007G215500 | 15.12 | 0.01828 | wall associated kinase 5 |
| Sobic.003G325000 | 14.94 | 0.01672 | Protein of unknown function (DUF594) |
| Sobic.006G211300 | 14.72 | 0.01846 | VACUOLAR SORTING RECEPTOR 7 |
| Sobic.003G258700 | 14.66 | 0.0355 | 0 |
| Sobic.004G102200 | 14.39 | 0.01411 | NOD26-like intrinsic protein 1;2 |
| Sobic.005G193500 | 14.19 | 0.00433 | receptor kinase 3 |
| Sobic.002G327300 | 13.36 | 0.01283 | cysteine-rich RLK (RECEPTOR-like protein kinase) 10 |
| Sobic.003G095400 | 13.32 | 0.03298 | Glycosyltransferase family 61 protein |
| Sobic.003G082401 | 13.2 | 0.01768 | Protein kinase superfamily protein |
| Sobic.003G388500 | 13.19 | 0.03172 | 0 |
| Sobic.005G193600 | 12.74 | 0.01768 | 3-ketoacyl-CoA synthase 2 |
| Sobic.003G031600 | 12.67 | 0.01599 | Protein of unknown function (DUF594) |
| Sobic.007G032900 | 12.41 | 0.01208 | PR5-like receptor kinase |
| Sobic.009G064400 | 12.12 | 0.00123 | cytochrome P450, family 71, subfamily B, polypeptide 37 |
| Sobic.010G247200 | 12.09 | 0.03159 | P-loop containing nucleoside triphosphate hydrolases superfamily protein |
| Sobic.006G177800 | 11.91 | 0.02174 | Prolyl oligopeptidase family protein |
| Sobic.005G230000 | 11.83 | 0.00809 | SCARECROW-like 14 |
| Sobic.001G350300 | 11.8 | 0.02237 | basic helix-loop-helix (bHLH) DNA-binding superfamily protein |
| Sobic.003G264400 | 11.49 | 0.00927 | glutathione S-transferase TAU 18 |
| Sobic.008G046300 | 11.43 | 0.00721 | Protein kinase superfamily protein |
| Sobic.001G314300 | 11.39 | 0.00996 | 2-oxoglutarate (2OG) and Fe(II)-dependent oxygenase superfamily protein |
| Sobic.001G235500 | 11.38 | 0.00752 | cytochrome P450, family 71, subfamily B, polypeptide 37 |
| Sobic.005G216200 | 11.36 | 0.03335 | O-methyltransferase family protein |
| Sobic.007G092400 | 11.34 | 0.0223 | lysine histidine transporter 1 |
| Sobic.003G047500 | 11.13 | 0.04488 | UDP-Glycosyltransferase superfamily protein |
| Sobic.003G323100 | 11.11 | 0.00972 | 0 |
| Sobic.010G045700 | 11 | 0.02546 | WRKY family transcription factor |
| Sobic.002G110200 | 10.72 | 0.02943 | cytochrome P450, family 71, subfamily B, polypeptide 34 |
| Sobic.003G111100 | 10.56 | 0.02534 | pathogenesis-related family protein |
| Sobic.006G098600 | 10.52 | 0.02237 | 0 |
| Sobic.005G082700 | 10.48 | 0.04151 | Zinc-binding dehydrogenase family protein |
| Sobic.002G328000 | 10.4 | 0.03104 | cysteine-rich RLK (RECEPTOR-like protein kinase) 8 |
| Sobic.001G261600 | 10.35 | 0.00486 | 0 |
| Sobic.003G430400 | 10.22 | 0.04607 | Calcium-binding EF-hand family protein |
| Sobic.002G040400 | 10.2 | 0.04812 | Cytochrome P450 superfamily protein |
| Sobic.008G191200 | 10.11 | 0.03216 | AAA-ATPase 1 |
| Sobic.005G216100 | 10.07 | 0.02473 | O-methyltransferase family protein |
| Sobic.003G144800 | 10.04 | 0.02419 | 0 |
| Sobic.005G076301 | 9.93 | 0.00328 | NB-ARC domain-containing disease resistance protein |
| Sobic.003G341100 | 9.87 | 0.02132 | WRKY DNA-binding protein 33 |
| Sobic.010G066700 | 9.83 | 0.01719 | hydroxycinnamoyl-CoA shikimate/quinate hydroxycinnamoyl transferase |
| Sobic.003G079900 | 9.73 | 0.00447 | Protein kinase superfamily protein |
| Sobic.003G096750 | 9.68 | 0.04408 | Protein kinase superfamily protein |
| Sobic.003G431100 | 9.62 | 0.02619 | Glycosyltransferase family 61 protein |
| Sobic.005G127800 | 9.62 | 0.03699 | disease resistance family protein / LRR family protein |
| Sobic.001G339200 | 9.61 | 0.03292 | 0 |
| Sobic.009G150000 | 9.59 | 0.03709 | Protein of unknown function (DUF1218) |
| Sobic.004G176900 | 9.48 | 0.01385 | mitogen-activated protein kinase kinase kinase 3 |
| Sobic.006G054900 | 9.24 | 0.04966 | Protein of unknown function, DUF538 |
| Sobic.001G353900 | 9.08 | 0.01774 | Malectin/receptor-like protein kinase family protein |
| Sobic.003G192400 | 9.04 | 0.01481 | receptor serine/threonine kinase, putative |
| Sobic.003G068200 | 8.98 | 0.03474 | Leucine-rich repeat protein kinase family protein |
| Sobic.010G193200 | 8.96 | 0.03733 | 0 |
| Sobic.005G222700 | 8.94 | 0.03447 | wall-associated kinase 2 |
| Sobic.003G413201 | 8.89 | 0.00245 | Protein kinase family protein with leucine-rich repeat domain |
| Sobic.001G050100 | 8.88 | 0.03179 | germin-like protein 4 |
| Sobic.007G227300 | 8.87 | 0.02473 | uclacyanin 1 |
| Sobic.009G107500 | 8.82 | 0.02678 | PAR1 protein |
| Sobic.009G073800 | 8.8 | 0.01953 | basic helix-loop-helix (bHLH) DNA-binding family protein |
| Sobic.009G180900 | 8.7 | 0.02419 | NB-ARC domain-containing disease resistance protein |
| Sobic.005G181200 | 8.67 | 0.04209 | Concanavalin A-like lectin protein kinase family protein |
| Sobic.002G020900 | 8.51 | 0.00964 | 0 |
| Sobic.002G416700 | 8.45 | 0.04594 | Peroxidase superfamily protein |
| Sobic.004G338000 | 8.3 | 0.04048 | phosphoenolpyruvate carboxylase kinase 1 |
| Sobic.003G413700 | 8.22 | 0.00164 | high affinity K+ transporter 5 |
| Sobic.006G185900 | 8.19 | 0.01882 | cytochrome P450, family 704, subfamily A, polypeptide 2 |
| Sobic.008G013800 | 8 | 0.01274 | Concanavalin A-like lectin protein kinase family protein |
| Sobic.002G193900 | 7.98 | 0.03807 | DNAse I-like superfamily protein |
| Sobic.002G018300 | 7.63 | 0.03104 | 0 |
| Sobic.009G210300 | 7.58 | 0.00428 | Calcium-binding EF-hand family protein |
| Sobic.003G065501 | 7.55 | 0.01879 | alpha/beta-Hydrolases superfamily protein |
| Sobic.009G230600 | 7.52 | 0.01621 | 0 |
| Sobic.005G171200 | 7.43 | 0.04119 | Major facilitator superfamily protein |
| Sobic.004G169600 | 7.4 | 0.01883 | Transducin/WD40 repeat-like superfamily protein |
| Sobic.002G185300 | 7.38 | 0.01727 | Major facilitator superfamily protein |
| Sobic.002G368100 | 7.38 | 0.00573 | COBRA-like protein 1 precursor |
| Sobic.010G272501 | 7.33 | 0.04656 | exocyst subunit exo70 family protein A2 |
| Sobic.003G233000 | 7.31 | 0.04956 | UDP-glucosyl transferase 73B5 |
| Sobic.009G034300 | 7.29 | 0.02141 | HXXXD-type acyl-transferase family protein |
| Sobic.010G035900 | 7.16 | 0.02615 | 0 |
| Sobic.006G201700 | 7.09 | 0.003 | Wall-associated kinase family protein |
| Sobic.006G152600 | 7.02 | 0.02766 | YELLOW STRIPE like 7 |
| Sobic.002G232800 | 7 | 0.02473 | RING/U-box superfamily protein |
| Sobic.005G075600 | 6.96 | 0.03525 | NB-ARC domain-containing disease resistance protein |
| Sobic.007G076000 | 6.93 | 0.02585 | cinnamyl alcohol dehydrogenase 9 |
| Sobic.005G122300 | 6.75 | 0.00728 | Beta-1,3-N-Acetylglucosaminyltransferase family protein |
| Sobic.009G036000 | 6.73 | 0.04028 | Low temperature and salt responsive protein family |
| Sobic.003G373000 | 6.59 | 0.04913 | myb domain protein 6 |
| Sobic.005G136200 | 6.57 | 0.02458 | Chalcone and stilbene synthase family protein |
| Sobic.002G377400 | 6.56 | 0.0271 | Concanavalin A-like lectin protein kinase family protein |
| Sobic.008G191300 | 6.54 | 0.04792 | AAA-ATPase 1 |
| Sobic.003G019700 | 6.48 | 0.03595 | GDSL-like Lipase/Acylhydrolase superfamily protein |
| Sobic.004G351200 | 6.46 | 0.04587 | plasma membrane intrinsic protein 1;4 |
| Sobic.004G219700 | 6.46 | 0.01523 | wall associated kinase 5 |
| Sobic.002G067300 | 6.27 | 0.02615 | P-loop containing nucleoside triphosphate hydrolases superfamily protein |
| Sobic.002G302300 | 6.17 | 0.04929 | Papain family cysteine protease |
| Sobic.003G340800 | 6 | 0.03104 | Transmembrane amino acid transporter family protein |
| Sobic.002G393900 | 5.98 | 0.04701 | NB-ARC domain-containing disease resistance protein |
| Sobic.005G207000 | 5.97 | 0.03943 | cysteine-rich RLK (RECEPTOR-like protein kinase) 39 |
| Sobic.007G035700 | 5.97 | 0.04604 | NAD(P)H dehydrogenase B2 |
| Sobic.008G185600 | 5.96 | 0.0357 | 0 |
| Sobic.004G099700 | 5.9 | 0.00573 | Protein kinase family protein with leucine-rich repeat domain |
| Sobic.010G052200 | 5.89 | 0.02509 | S-adenosyl-L-methionine-dependent methyltransferases superfamily protein |
| Sobic.001G215900 | 5.86 | 0.02712 | cysteine-rich RLK (RECEPTOR-like protein kinase) 2 |
| Sobic.002G024100 | 5.79 | 0.01782 | Concanavalin A-like lectin protein kinase family protein |
| Sobic.001G483600 | 5.72 | 0.01467 | Protein kinase superfamily protein |
| Sobic.004G061400 | 5.65 | 0.0007 | autoinhibited Ca2+ -ATPase, isoform 8 |
| Sobic.003G002900 | 5.47 | 0.02671 | YELLOW STRIPE like 7 |
| Sobic.008G006300 | 5.41 | 0.01668 | prenylated RAB acceptor 1.B5 |
| Sobic.010G122400 | 5.33 | 0.01652 | Ankyrin repeat family protein |
| Sobic.004G192100 | 5.32 | 0.01993 | PLANT CADMIUM RESISTANCE 2 |
| Sobic.003G143100 | 5.27 | 0.02812 | senescence-related gene 1 |
| Sobic.001G476300 | 5.15 | 0.01666 | homeobox protein 16 |
| Sobic.003G291600 | 5.1 | 0.01047 | Protein kinase family protein with leucine-rich repeat domain |
| Sobic.002G371900 | 5.09 | 0.04389 | Cysteine/Histidine-rich C1 domain family protein |
| Sobic.003G384700 | 5.05 | 0.03872 | Pectinacetylesterase family protein |
| Sobic.001G071300 | 5.05 | 0.01188 | 0 |
| Sobic.001G389700 | 5.02 | 0.03078 | calmodulin (CAM)-binding protein of 25 kDa |
| Sobic.010G015800 | 5 | 0.01333 | RING/U-box superfamily protein |
| Sobic.009G181300 | 4.97 | 0.02438 | cysteine-rich RLK (RECEPTOR-like protein kinase) 25 |
| Sobic.004G187300 | 4.94 | 0.03609 | 0 |
| Sobic.006G068800 | 4.93 | 0.02652 | alpha/beta-Hydrolases superfamily protein |
| Sobic.003G325300 | 4.85 | 0.04462 | Disease resistance protein (CC-NBS-LRR class) family |
| Sobic.001G516000 | 4.83 | 0.00127 | basic chitinase |
| Sobic.003G440900 | 4.83 | 0.02739 | beta-fructofuranosidase 5 |
| Sobic.002G321300 | 4.78 | 0.02381 | pleiotropic drug resistance 6 |
| Sobic.002G131800 | 4.76 | 0.03173 | RING domain ligase1 |
| Sobic.002G024000 | 4.59 | 0.02787 | receptor lectin kinase |
| Sobic.006G084600 | 4.58 | 0.00973 | bidirectional amino acid transporter 1 |
| Sobic.006G250200 | 4.52 | 0.02434 | amino acid permease 7 |
| Sobic.005G039100 | 4.47 | 0.00896 | 0 |
| Sobic.001G158700 | 4.46 | 0.02838 | ankyrin repeat family protein |
| Sobic.004G106500 | 4.44 | 0.04878 | UDP-Glycosyltransferase superfamily protein |
| Sobic.004G129900 | 4.44 | 0.02993 | MATE efflux family protein |
| Sobic.008G042900 | 4.43 | 0.04581 | NDR1/HIN1-like 3 |
| Sobic.006G200000 | 4.37 | 0.02726 | RNA-binding (RRM/RBD/RNP motifs) family protein |
| Sobic.010G080500 | 4.31 | 0.02331 | S-adenosyl-L-methionine-dependent methyltransferases superfamily protein |
| Sobic.001G365700 | 4.29 | 0.00986 | rhomboid protein-related |
| Sobic.009G043600 | 4.21 | 0.0278 | Glutathione S-transferase family protein |
| Sobic.005G061200 | 4.17 | 0.01006 | Disease resistance-responsive (dirigent-like protein) family protein |
| Sobic.007G033100 | 4.13 | 0.02505 | receptor like protein 26 |
| Sobic.010G084900 | 4.1 | 0.0224 | Protein kinase superfamily protein |
| Sobic.001G474200 | 4.06 | 0.0345 | Ras-related small GTP-binding family protein |
| Sobic.006G104200 | 4.02 | 0.01208 | 0 |
| Sobic.007G194400 | 4.02 | 0.0215 | exocyst subunit exo70 family protein F1 |
| Sobic.007G210500 | 3.98 | 0.03526 | multidrug resistance-associated protein 14 |
| Sobic.001G459800 | 3.98 | 0.02473 | UDP-glucose 6-dehydrogenase family protein |
| Sobic.001G295100 | 3.97 | 0.02438 | PRA1 (Prenylated rab acceptor) family protein |
| Sobic.001G084100 | 3.93 | 0.01578 | UDP-glucose 6-dehydrogenase family protein |
| Sobic.004G111200 | 3.91 | 0.04486 | Protein kinase family protein with leucine-rich repeat domain |
| Sobic.010G023200 | 3.9 | 0.03585 | 0 |
| Sobic.001G505600 | 3.9 | 0.04035 | DENN (AEX-3) domain-containing protein |
| Sobic.005G094800 | 3.89 | 0.01006 | cysteine-rich RLK (RECEPTOR-like protein kinase) 40 |
| Sobic.001G314700 | 3.83 | 0.04141 | PLAC8 family protein |
| Sobic.001G326200 | 3.79 | 0.03134 | ZCF37 |
| Sobic.003G401200 | 3.79 | 0.02374 | selenium-binding protein 1 |
| Sobic.010G040100 | 3.76 | 0.01014 | 0 |
| Sobic.001G491800 | 3.76 | 0.03377 | Protein of unknown function (DUF1997) |
| Sobic.004G011700 | 3.73 | 0.00558 | Heat shock protein 70 (Hsp 70) family protein |
| Sobic.001G159200 | 3.71 | 0.02224 | S-domain-2 5 |
| Sobic.006G062900 | 3.7 | 0.04619 | SBP (S-ribonuclease binding protein) family protein |
| Sobic.006G038800 | 3.69 | 0.0155 | purple acid phosphatase 27 |
| Sobic.001G173300 | 3.66 | 0.04851 | reversibly glycosylated polypeptide 2 |
| Sobic.002G041100 | 3.61 | 0.02956 | Tyrosine transaminase family protein |
| Sobic.002G300000 | 3.6 | 0.0264 | expansin-like A1 |
| Sobic.001G523866 | 3.6 | 0.03242 | 0 |
| Sobic.004G220000 | 3.55 | 0.01953 | adenosine kinase 2 |
| Sobic.006G146000 | 3.49 | 0.04994 | beta-glucosidase 47 |
| Sobic.004G323900 | 3.47 | 0.01378 | stress-inducible protein, putative |
| Sobic.005G092900 | 3.43 | 0.03194 | 0 |
| Sobic.005G210200 | 3.42 | 0.03404 | cysteine-rich RLK (RECEPTOR-like protein kinase) 15 |
| Sobic.003G018800 | 3.4 | 0.04656 | SGNH hydrolase-type esterase superfamily protein |
| Sobic.001G148900 | 3.4 | 0.02883 | Cobalamin-independent synthase family protein |
| Sobic.003G316300 | 3.39 | 0.00558 | Insulinase (Peptidase family M16) family protein |
| Sobic.010G267400 | 3.39 | 0.02458 | Chaperone protein htpG family protein |
| Sobic.007G146600 | 3.35 | 0.02805 | sec34-like family protein |
| Sobic.003G277500 | 3.35 | 0.02437 | S-adenosyl-L-methionine-dependent methyltransferases superfamily protein |
| Sobic.001G234900 | 3.29 | 0.02438 | phosphate transporter 1;7 |
| Sobic.003G112600 | 3.28 | 0.02057 | endoplasmic reticulum oxidoreductins 1 |
| Sobic.003G327600 | 3.27 | 0.02232 | Nucleotide-sugar transporter family protein |
| Sobic.002G201900 | 3.25 | 0.03558 | sugar transporter protein 7 |
| Sobic.009G157700 | 3.25 | 0.03088 | phosphate transporter 4;2 |
| Sobic.009G014000 | 3.25 | 0.03991 | ankyrin repeat family protein |
| Sobic.K043400 | 3.24 | 0.00245 | homolog of mamallian P58IPK |
| Sobic.007G111300 | 3.23 | 0.03912 | Endomembrane protein 70 protein family |
| Sobic.006G185300 | 3.23 | 0.04752 | peptidoglycan-binding LysM domain-containing protein |
| Sobic.010G268400 | 3.23 | 0.02046 | Eukaryotic aspartyl protease family protein |
| Sobic.003G037800 | 3.22 | 0.01675 | HXXXD-type acyl-transferase family protein |
| Sobic.004G257000 | 3.21 | 0.02549 | Endomembrane protein 70 protein family |
| Sobic.010G219800 | 3.21 | 0.00425 | VACUOLAR SORTING RECEPTOR 6 |
| Sobic.010G236500 | 3.2 | 0.01652 | Bifunctional inhibitor/lipid-transfer protein/seed storage 2S albumin superfamily protein |
| Sobic.001G469100 | 3.2 | 0.02598 | C2H2-like zinc finger protein |
| Sobic.005G116300 | 3.12 | 0.01398 | Pyridoxal phosphate (PLP)-dependent transferases superfamily protein |
| Sobic.002G104900 | 3.11 | 0.03628 | B12D protein |
| Sobic.005G093400 | 3.1 | 0.02419 | 0 |
| Sobic.001G123100 | 3.08 | 0.00927 | B-S glucosidase 44 |
| Sobic.005G095900 | 3.08 | 0.02389 | cysteine-rich RLK (RECEPTOR-like protein kinase) 20 |
| Sobic.003G413600 | 3.07 | 0.03313 | high affinity K+ transporter 5 |
| Sobic.002G248100 | 3.05 | 0.02883 | 0 |
| Sobic.001G012800 | 3.03 | 0.02541 | Nucleotide-diphospho-sugar transferase family protein |
| Sobic.001G504500 | 3.03 | 0.02225 | 0 |
| Sobic.004G329100 | 2.99 | 0.04097 | 2-oxoglutarate (2OG) and Fe(II)-dependent oxygenase superfamily protein |
| Sobic.003G235300 | 2.98 | 0.02379 | P-loop containing nucleoside triphosphate hydrolases superfamily protein |
| Sobic.002G153000 | 2.98 | 0.01705 | NAD(P)-binding Rossmann-fold superfamily protein |
| Sobic.003G191500 | 2.96 | 0.00893 | Preprotein translocase Sec, Sec61-beta subunit protein |
| Sobic.001G418100 | 2.94 | 0.03488 | UDP-XYL synthase 6 |
| Sobic.003G036300 | 2.93 | 0.01869 | 2-oxoglutarate (2OG) and Fe(II)-dependent oxygenase superfamily protein |
| Sobic.008G060500 | 2.92 | 0.01928 | Protein kinase family protein with leucine-rich repeat domain |
| Sobic.007G198100 | 2.91 | 0.01208 | Auxin-responsive family protein |
| Sobic.001G040300 | 2.89 | 0.00262 | Sphingomyelin synthetase family protein |
| Sobic.002G262800 | 2.83 | 0.0178 | FAD-dependent oxidoreductase family protein |
| Sobic.007G026500 | 2.81 | 0.01511 | Galactose oxidase/kelch repeat superfamily protein |
| Sobic.005G026600 | 2.78 | 0.01928 | Phosphoglycerate mutase family protein |
| Sobic.004G308100 | 2.77 | 0.01006 | PLAC8 family protein |
| Sobic.002G393800 | 2.75 | 0.02352 | NB-ARC domain-containing disease resistance protein |
| Sobic.004G185900 | 2.72 | 0.02381 | Galactosyltransferase family protein |
| Sobic.005G154300 | 2.72 | 0.01928 | Disease resistance protein (CC-NBS-LRR class) family |
| Sobic.003G346200 | 2.71 | 0.03803 | ROP interactive partner 3 |
| Sobic.009G247300 | 2.7 | 0.02946 | WRKY DNA-binding protein 28 |
| Sobic.001G185500 | 2.7 | 0.02726 | MATE efflux family protein |
| Sobic.008G187300 | 2.69 | 0.03608 | 0 |
| Sobic.006G159800 | 2.68 | 0.01774 | Arabidopsis thaliana protein of unknown function (DUF821) |
| Sobic.001G205900 | 2.66 | 0.03969 | 2-oxoglutarate (2OG) and Fe(II)-dependent oxygenase superfamily protein |
| Sobic.009G082200 | 2.65 | 0.01124 | cysteine-rich RLK (RECEPTOR-like protein kinase) 10 |
| Sobic.007G227500 | 2.65 | 0.04035 | 0 |
| Sobic.008G079300 | 2.65 | 0.03972 | Glutathione S-transferase family protein |
| Sobic.003G138400 | 2.65 | 0.0248 | WRKY family transcription factor |
| Sobic.003G350200 | 2.6 | 0.03323 | 0 |
| Sobic.002G373900 | 2.57 | 0.03242 | Lung seven transmembrane receptor family protein |
| Sobic.009G220200 | 2.56 | 0.01719 | Exostosin family protein |
| Sobic.001G231200 | 2.55 | 0.0472 | 0 |
| Sobic.002G317800 | 2.53 | 0.01978 | BNR/Asp-box repeat family protein |
| Sobic.001G329100 | 2.44 | 0.01778 | Leucine-rich repeat (LRR) family protein |
| Sobic.005G192100 | 2.44 | 0.03922 | NB-ARC domain-containing disease resistance protein |
| Sobic.007G183400 | 2.44 | 0.01513 | LRR and NB-ARC domains-containing disease resistance protein |
| Sobic.005G005600 | 2.43 | 0.03171 | prenylated RAB acceptor 1.B4 |
| Sobic.004G000400 | 2.41 | 0.03526 | PDI-like 1-4 |
| Sobic.001G516600 | 2.41 | 0.04253 | OPC-8:0 CoA ligase1 |
| Sobic.007G003100 | 2.4 | 0.01168 | 0 |
| Sobic.001G499000 | 2.37 | 0.01588 | potassium channel tetramerisation domain-containing protein / pentapeptide repeat-containing protein |
| Sobic.003G348200 | 2.37 | 0.02387 | Lung seven transmembrane receptor family protein |
| Sobic.004G230300 | 2.36 | 0.035 | Nucleic acid-binding, OB-fold-like protein |
| Sobic.007G164300 | 2.35 | 0.04708 | rotamase cyclophilin 5 |
| Sobic.001G536200 | 2.32 | 0.01928 | 0 |
| Sobic.002G085700 | 2.32 | 0.03542 | UDP-Glycosyltransferase superfamily protein |
| Sobic.008G133500 | 2.3 | 0.04805 | diaminopimelate epimerase family protein |
| Sobic.006G063700 | 2.28 | 0.03293 | F-box/RNI-like/FBD-like domains-containing protein |
| Sobic.002G138500 | 2.26 | 0.0355 | 0 |
| Sobic.002G254650 | 2.24 | 0.02549 | 0 |
| Sobic.004G062500 | 2.24 | 0.03293 | 4-coumarate:CoA ligase 2 |
| Sobic.004G024400 | 2.23 | 0.03061 | RNA-binding KH domain-containing protein |
| Sobic.005G022200 | 2.22 | 0.0306 | exoribonuclease 2 |
| Sobic.003G093400 | 2.21 | 0.02046 | 0 |
| Sobic.006G271300 | 2.18 | 0.04003 | Calcium-dependent lipid-binding (CaLB domain) family protein |
| Sobic.003G445000 | 2.18 | 0.01124 | Pectinacetylesterase family protein |
| Sobic.009G152600 | 2.18 | 0.02695 | pyrophosphorylase 3 |
| Sobic.001G445500 | 2.13 | 0.04664 | coatomer gamma-2 subunit, putative / gamma-2 coat protein, putative / gamma-2 COP, putative |
| Sobic.004G330600 | 2.13 | 0.01787 | GHMP kinase family protein |
| Sobic.010G167500 | 2.12 | 0.04558 | Protein kinase family protein with leucine-rich repeat domain |
| Sobic.007G161400 | 2.11 | 0.01871 | Peroxisomal membrane 22 kDa (Mpv17/PMP22) family protein |
| Sobic.002G407300 | 2.07 | 0.00595 | NAD(P)-binding Rossmann-fold superfamily protein |
| Sobic.005G087100 | 2.07 | 0.04187 | glycine-rich protein |
| Sobic.001G026900 | 2.06 | 0.04008 | Cytidine/deoxycytidylate deaminase family protein |
| Sobic.004G341200 | 2.05 | 0.01767 | glutathione-disulfide reductase |
| Sobic.004G267500 | 2.04 | 0.00853 | Adenine nucleotide alpha hydrolases-like superfamily protein |
| Sobic.005G048700 | 2.01 | 0.04452 | NAD(P)-binding Rossmann-fold superfamily protein |
| Sobic.010G050800 | -2 | 0.01063 | alpha/beta-Hydrolases superfamily protein |
| Sobic.004G055500 | -2.01 | 0.03376 | myo-inositol monophosphatase like 1 |
| Sobic.006G086900 | -2.03 | 0.04701 | Plant invertase/pectin methylesterase inhibitor superfamily |
| Sobic.004G030100 | -2.03 | 0.0386 | anthranilate phosphoribosyltransferase, putative |
| Sobic.001G308000 | -2.04 | 0.02849 | 0 |
| Sobic.002G065200 | -2.05 | 0.04726 | Tetratricopeptide repeat (TPR)-like superfamily protein |
| Sobic.004G055700 | -2.06 | 0.01237 | Single hybrid motif superfamily protein |
| Sobic.010G195200 | -2.06 | 0.02094 | DCD (Development and Cell Death) domain protein |
| Sobic.001G410700 | -2.07 | 0.03179 | Tetratricopeptide repeat (TPR)-like superfamily protein |
| Sobic.007G163001 | -2.07 | 0.04172 | 0 |
| Sobic.006G273400 | -2.09 | 0.04851 | pentatricopeptide (PPR) repeat-containing protein |
| Sobic.006G177600 | -2.09 | 0.0421 | 0 |
| Sobic.003G205100 | -2.12 | 0.01851 | RAD3-like DNA-binding helicase protein |
| Sobic.010G242100 | -2.16 | 0.03336 | cytochrome P450, family 716, subfamily A, polypeptide 1 |
| Sobic.003G115000 | -2.17 | 0.046 | 0 |
| Sobic.008G102000 | -2.2 | 0.04292 | Chaperone DnaJ-domain superfamily protein |
| Sobic.005G030700 | -2.2 | 0.03292 | TRAF-like family protein |
| Sobic.001G425100 | -2.21 | 0.04755 | DA1-related protein 2 |
| Sobic.006G016700 | -2.22 | 0.03773 | RNA-dependent RNA polymerase family protein |
| Sobic.002G000700 | -2.24 | 0.0418 | 0 |
| Sobic.004G132600 | -2.25 | 0.01684 | chloroplastic NIFS-like cysteine desulfurase |
| Sobic.001G403800 | -2.25 | 0.03741 | 0 |
| Sobic.001G090200 | -2.25 | 0.04039 | NDH-dependent cyclic electron flow 5 |
| Sobic.008G186400 | -2.28 | 0.03741 | Leucine-rich repeat transmembrane protein kinase family protein |
| Sobic.001G067850 | -2.28 | 0.04851 | 0 |
| Sobic.010G199600 | -2.29 | 0.0464 | nudix hydrolase homolog 2 |
| Sobic.004G193200 | -2.3 | 0.03311 | Pseudouridine synthase family protein |
| Sobic.004G107550 | -2.35 | 0.04913 | 0 |
| Sobic.005G007800 | -2.35 | 0.00709 | RNI-like superfamily protein |
| Sobic.010G219700 | -2.37 | 0.01719 | Inositol monophosphatase family protein |
| Sobic.007G223500 | -2.37 | 0.04003 | 0 |
| Sobic.002G289400 | -2.4 | 0.04878 | S-locus lectin protein kinase family protein |
| Sobic.003G222500 | -2.4 | 0.03304 | phosphoglucomutase |
| Sobic.002G305700 | -2.43 | 0.04966 | NDH-dependent cyclic electron flow 1 |
| Sobic.001G495300 | -2.44 | 0.01676 | Protein of unknown function (DUF1012) |
| Sobic.001G041800 | -2.45 | 0.01239 | ALBINA 1 |
| Sobic.002G215000 | -2.45 | 0.0436 | photosystem I light harvesting complex gene 6 |
| Sobic.004G251500 | -2.45 | 0.03768 | calcium sensing receptor |
| Sobic.004G114800 | -2.46 | 0.02721 | thylakoid rhodanese-like |
| Sobic.009G034500 | -2.46 | 0.02989 | ATP binding cassette subfamily B4 |
| Sobic.001G106600 | -2.48 | 0.04095 | Sulfite exporter TauE/SafE family protein |
| Sobic.005G042000 | -2.49 | 0.03595 | ribulose bisphosphate carboxylase small chain 1A |
| Sobic.001G531800 | -2.5 | 0.04877 | Dihydrodipicolinate reductase, bacterial/plant |
| Sobic.001G100000 | -2.5 | 0.04664 | ADP glucose pyrophosphorylase large subunit 1 |
| Sobic.003G013100 | -2.5 | 0.02588 | GDSL-like Lipase/Acylhydrolase superfamily protein |
| Sobic.003G137100 | -2.5 | 0.02818 | Flavin containing amine oxidoreductase family |
| Sobic.003G255100 | -2.51 | 0.0113 | 0 |
| Sobic.004G326200 | -2.53 | 0.04544 | RNA polymerase II large subunit |
| Sobic.006G248700 | -2.53 | 0.02696 | P-loop containing nucleoside triphosphate hydrolases superfamily protein |
| Sobic.004G016700 | -2.54 | 0.03627 | 0 |
| Sobic.010G092100 | -2.55 | 0.0446 | TEOSINTE BRANCHED, cycloidea and PCF (TCP) 14 |
| Sobic.006G271800 | -2.55 | 0.01814 | K+ efflux antiporter 2 |
| Sobic.010G249600 | -2.55 | 0.04515 | Tautomerase/MIF superfamily protein |
| Sobic.010G143500 | -2.59 | 0.01014 | Pyruvate phosphate dikinase, PEP/pyruvate binding domain |
| Sobic.005G213100 | -2.59 | 0.0459 | 0 |
| Sobic.001G138800 | -2.61 | 0.03178 | glycine-rich protein |
| Sobic.010G038600 | -2.64 | 0.01879 | 0 |
| Sobic.001G425400 | -2.69 | 0.03749 | high cyclic electron flow 1 |
| Sobic.004G310100 | -2.75 | 0.03171 | flavonol synthase 1 |
| Sobic.005G068200 | -2.76 | 0.04405 | 0 |
| Sobic.009G020100 | -2.77 | 0.01684 | nudix hydrolase homolog 9 |
| Sobic.002G145200 | -2.78 | 0.03469 | 0 |
| Sobic.003G299200 | -2.8 | 0.03948 | Pentatricopeptide repeat (PPR) superfamily protein |
| Sobic.006G269400 | -2.8 | 0.02867 | 0 |
| Sobic.006G166000 | -2.81 | 0.03872 | Tetratricopeptide repeat (TPR)-like superfamily protein |
| Sobic.001G236800 | -2.82 | 0.02683 | phosphoglycerate kinase 1 |
| Sobic.002G020500 | -2.85 | 0.03601 | Protein of unknown function, DUF547 |
| Sobic.001G316100 | -2.87 | 0.0453 | Protein of unknown function (DUF3464) |
| Sobic.003G044900 | -2.92 | 0.02051 | AUX/IAA transcriptional regulator family protein |
| Sobic.009G253000 | -2.94 | 0.0325 | RNApolymerase sigma-subunit C |
| Sobic.009G070600 | -2.94 | 0.0303 | HAD superfamily, subfamily IIIB acid phosphatase |
| Sobic.002G031600 | -2.94 | 0.03104 | Putative membrane lipoprotein |
| Sobic.009G085100 | -2.96 | 0.039 | indoleacetic acid-induced protein 16 |
| Sobic.007G190100 | -3.14 | 0.03967 | Transketolase family protein |
| Sobic.007G090442 | -3.26 | 0.02949 | 0 |
| Sobic.001G019700 | -3.39 | 0.04078 | MATE efflux family protein |
| Sobic.K028200 | -3.39 | 0.03976 | cytochrome p450 78a9 |
| Sobic.003G281200 | -3.46 | 0.01777 | phosphoribosyl pyrophosphate (PRPP) synthase 3 |
| Sobic.004G170600 | -3.49 | 0.04137 | GDSL-like Lipase/Acylhydrolase superfamily protein |
| Sobic.001G225500 | -3.52 | 0.04913 | 0 |
| Sobic.008G157000 | -3.52 | 0.04347 | AUX/IAA transcriptional regulator family protein |
| Sobic.004G060900 | -3.59 | 0.03194 | Leucine-rich repeat protein kinase family protein |
| Sobic.002G430400 | -3.63 | 0.03706 | 0 |
| Sobic.003G131000 | -3.69 | 0.04143 | 0 |
| Sobic.001G465000 | -3.72 | 0.03872 | 0 |
| Sobic.002G356800 | -3.83 | 0.02967 | GDSL-like Lipase/Acylhydrolase superfamily protein |
| Sobic.002G192700 | -3.89 | 0.01465 | alpha/beta-Hydrolases superfamily protein |
| Sobic.005G096101 | -3.93 | 0.00393 | Bifunctional inhibitor/lipid-transfer protein/seed storage 2S albumin superfamily protein |
| Sobic.002G072700 | -3.94 | 0.04664 | Protein of Unknown Function (DUF239) |
| Sobic.002G218100 | -3.97 | 0.027 | S-adenosyl-L-methionine-dependent methyltransferases superfamily protein |
| Sobic.004G165600 | -4.01 | 0.04108 | Glycosyl hydrolase superfamily protein |
| Sobic.005G155200 | -4.09 | 0.04292 | phytosulfokine 2 precursor |
| Sobic.002G175500 | -4.24 | 0.03242 | serine carboxypeptidase-like 17 |
| Sobic.009G070700 | -4.25 | 0.04763 | HAD superfamily, subfamily IIIB acid phosphatase |
| Sobic.009G002600 | -4.28 | 0.04686 | polygalacturonase inhibiting protein 1 |
| Sobic.007G090439 | -4.45 | 0.03274 | 0 |
| Sobic.001G383400 | -4.65 | 0.01375 | cytochrome P450, family 714, subfamily A, polypeptide 1 |
| Sobic.004G233500 | -4.67 | 0.0382 | Polymerase/histidinol phosphatase-like |
| Sobic.003G363600 | -5.13 | 0.03494 | bZIP transcription factor family protein |
| Sobic.001G258100 | -5.46 | 0.01133 | Subtilase family protein |
| Sobic.002G093500 | -5.63 | 0.03159 | Peptidase M20/M25/M40 family protein |
| Sobic.006G025500 | -5.64 | 0.00752 | 0 |
| Sobic.010G070200 | -5.65 | 0.04754 | UDP-glucosyl transferase 78D2 |
| Sobic.010G146100 | -5.89 | 0.03105 | delta tonoplast integral protein |
| Sobic.002G221900 | -5.92 | 0.029 | ethylene-forming enzyme |
| Sobic.003G291000 | -6.6 | 0.01985 | protein kinase family protein / peptidoglycan-binding LysM domain-containing protein |
| Sobic.004G232900 | -7.18 | 0.02549 | Haloacid dehalogenase-like hydrolase (HAD) superfamily protein |
| Sobic.006G025600 | -7.31 | 0.00407 | 0 |
| Sobic.003G118800 | -7.54 | 0.04019 | 0 |
| Sobic.006G151500 | -9.54 | 0.01725 | cytokinin oxidase/dehydrogenase 6 |
| Sobic.003G397300 | -9.58 | 0.03856 | 0 |
| Sobic.008G012150 | -9.76 | 0.03977 | 0 |
| Sobic.006G025400 | -9.84 | 0.00046 | 0 |
| Sobic.010G217466 | -9.95 | 0.02649 | 0 |
| Sobic.004G121900 | -10.36 | 0.02224 | expansin 11 |
| Sobic.005G041400 | -11.06 | 0.04292 | AP2/B3-like transcriptional factor family protein |
| Sobic.001G464700 | -13.91 | 0.01204 | 0 |

## Supplementary Table 12. Number of differentially expressed modules identified by weighted correlation network analysis (WGCNA) in maize and sorghum in response to *S. turcica.*

| **Crop** | **Interaction** | **Time** | **Total modules** | **Number of differentially expressed modules** | |
| --- | --- | --- | --- | --- | --- |
|  |  |  |  | **Down** | **Up** |
| Maize | Compatible | 24 hai | 58 | 0 | 1 |
| Maize | Incompatible | 24 hai | 58 | 0 | 0 |
| Maize | Compatible | 72 hai | 58 | 0 | 1 |
| Maize | Incompatible | 72 hai | 58 | 6 | 3 |
| Sorghum | Compatible | 24 hai | 33 | 5 | 6 |
| Sorghum | Incompatible | 24 hai | 33 | 0 | 0 |
| Sorghum | Compatible | 72 hai | 33 | 0 | 0 |
| Sorghum | Incompatible | 72 hai | 33 | 6 | 6 |

# Supplementary Table 13. Summary of modules that are significantly associated (FDR <0.05) with at least one of the four interaction-time combinations in each maize and sorghum.

| **Crop** | **Modules** | **No. of genes** | **Expression** | **Significant Interaction** |
| --- | --- | --- | --- | --- |
| **Maize** | Module 6 | 1277 | Upregulated | Comp 24, Comp 72, Incomp 72 |
| **Maize** | Module 40 | 80 | Upregulated | Incomp 72 |
| **Maize** | Module 50 | 53 | Upregulated | Incomp 72 |
| **Maize** | Module 18 | 251 | Downregulated | Incomp 72 |
| **Maize** | Module 34 | 86 | Downregulated | Incomp 72 |
| **Maize** | Module 38 | 83 | Downregulated | Incomp 72 |
| **Maize** | Module 45 | 68 | Downregulated | Incomp 72 |
| **Maize** | Module 48 | 58 | Downregulated | Incomp 72 |
| **Maize** | Module 55 | 40 | Downregulated | Incomp 72 |
| **Sorghum** | Module 8 | 828 | Upregulated | Comp 24, Incomp 72 |
| **Sorghum** | Module 11 | 676 | Upregulated | Comp 24, Incomp 72 |
| **Sorghum** | Module 19 | 200 | Upregulated | Comp 24, Incomp 72 |
| **Sorghum** | Module 15 | 374 | Upregulated | Comp 24 |
| **Sorghum** | Module 23 | 102 | Upregulated | Comp 24 |
| **Sorghum** | Module 25 | 85 | Upregulated | Comp 24 |
| **Sorghum** | Module 12 | 513 | Upregulated | Incomp 72 |
| **Sorghum** | Module 31 | 26 | Upregulated | Incomp 72 |
| **Sorghum** | Module 32 | 22 | Upregulated | Incomp 72 |
| **Sorghum** | Module 1 | 1949 | Downregulated | Comp 24, Incomp 72 |
| **Sorghum** | Module 24 | 88 | Downregulated | Comp 24, Incomp 72 |
| **Sorghum** | Module 14 | 392 | Downregulated | Comp 24, Incomp 72 |
| **Sorghum** | Module 2 | 1791 | Downregulated | Comp 24 |
| **Sorghum** | Module 28 | 63 | Downregulated | Comp 24 |
| **Sorghum** | Module 20 | 130 | Downregulated | Incomp 72 |
| **Sorghum** | Module 29 | 43 | Downregulated | Incomp 72 |
| **Sorghum** | Module 3 | 1557 | Downregulated | Incomp 72 |

Comp 24: Compatible interaction at 24 hai

Comp 72: Compatible interaction at 72hai

Incomp 24: Incompatible interaction at 24 hai

Incomp 72: Incompatible interaction at 72 hai
